# Supplementary material for: Enamel Matrix Derivative and Autogenous Bone Graft for Periodontal Regeneration of Intrabony Defects in Humans: A Systematic Review and Meta-Analysis
Source: Materials (Basel). 2019 Aug 19;12(16):2634. doi: 10.3390/ma12162634 (PMC6719005; doi:10.3390/ma12162634)
Supplement: Supplementary file 1 [file materials-12-02634-s001.pdf]

Supplementary material

List of excluded studies with the reason for exclusion

Abukawa H, Zhang W, Young CS, Asrican R, Vacanti JP, Kaban LB, Troulis MJ, Yelick PC. Reconstructing mandibular defects using autologous tissue-engineered tooth and bone constructs. J Oral Maxillofac Surg. 2009 Feb;67(2):335-47. doi: 10.1016/j.joms.2008.09.002. PubMed PMID: 19138608.
Reason: Not related to the clinical question

Abu-Ta'a M. Adjunctive Systemic Antimicrobial Therapy vs Asepsis in Conjunction with Guided Tissue Regeneration: A Randomized, Controlled Clinical Trial. J Contemp Dent Pract. 2016 Jan 1;17(1):3-6. PubMed PMID: 27084855.
Reason: Not related to the clinical question

Afrashtehfar KI, Moshaverinia A. Five Things to Know About Regenerative Periodontal Therapies in Dental Medicine. J N J Dent Assoc. 2015 Spring;86(2):12-3. PubMed PMID: 26242103.
Reason: Not related to the clinical question

Agrali OB, Kuru BE. Periodontal treatment in a generalized severe chronic periodontitis patient: A case report with 7-year follow-up. Eur J Dent. 2015 Apr-Jun;9(2):288-92. doi: 10.4103/1305-7456.156844. PubMed PMID: 26038666; PubMed Central PMCID: PMC4439862.
Reason: Not related to the clinical question

Aimetti M, Ferrarotti F, Mariani G, Fratini A, Giraudi M, Romano F. Enamel Matrix Derivative Proteins in Combination with a Flapless Approach for Periodontal Regeneration of Intrabony Defects: A 2-Year Prospective Case Series. Int J Periodontics Restorative Dent. 2016 Nov/Dec;36(6):797-805. doi: 10.11607/prd.2842. PubMed PMID: 27740640.
Reason: Autologous bone wasn't used

Aimetti M, Pigella E, Romano F, Debernardi C. Treatment of mandibular class II furcation defects by the use of amelogenins and autologous bone. Two case reports. Minerva Stomatol. 2005 Oct;54(10):583-91. English, Italian. PubMed PMID: 16224378.
Reason: Not a RCT

Aimetti M, Romano F, Pigella E, Piemontese M. Clinical evaluation of the effectiveness of enamel matrix proteins and autologous bone graft in the treatment of mandibular Class II furcation defects: a series of 11 patients. Int J Periodontics Restorative Dent. 2007 Oct;27(5):441-7. PubMed PMID: 17990440.
Reason: Not a RCT

Ajwani H, Shetty S, Gopalakrishnan D, Kathariya R, Kulloli A, Dolas RS, Pradeep AR. Comparative evaluation of platelet-rich fibrin biomaterial and open flap debridement in the treatment of two and three wall intrabony defects. J Int Oral Health. 2015 Apr;7(4):32-7. PubMed PMID: 25954068; PubMed Central PMCID: PMC4409793.
Reason: Not related to the clinical question

Akdemir O, Lineaweaver WC, Celik S, Cınar C, Zhang F. Submandibular artery: bilobed platysma myocutaneous flap for total lower lip reconstruction. J Craniomaxillofac Surg. 2014 Dec;42(8):1861-7. doi: 10.1016/j.jcms.2014.07.004. Epub 2014 Aug 14. PubMed PMID: 25209383
Al-Dabbagh NN, Zahid TM. The impact of fatwas on patients' acceptance of enamel matrix derivatives for periodontal regeneration in Saudi Arabia. Patient Prefer Adherence. 2018 Nov 12;12:2405-2411. doi: 10.2147/PPA.S179629. eCollection 2018. PubMed PMID: 30519006; PubMed Central PMCID: PMC6239119.
Reason: Not related to the clinical question

Alexiou A, Vouros I, Menexes G, Konstantinidis A. Comparison of enamel matrix derivative (Emdogain) and subepithelial connective tissue graft for root coverage in patients with multiple gingival recession defects: A randomized controlled clinical study. Quintessence Int. 2017;48(5):381-389. doi: 10.3290/j.qi.a38058. PubMed PMID: 28396887.
Reason: Autologous bone wasn't used

Al-Hezaimi K, Al-Askar M, Al-Rasheed A. Characteristics of newly-formed cementum following Emdogain application. Int J Oral Sci. 2011 Jan;3(1):21-6. doi: 10.4248/IJOS11009. PubMed PMID: 21449212; PubMed Central PMCID: PMC3469871.

Reason: Autologous bone wasn't used

Alhezaimi K, Al-Shalan T, O'Neill R, Shapurian T, Naghshbandi J, Levi P Jr, Griffin T. Connective tissue-cementum regeneration: a new histologic regeneration following the use of enamel matrix derivative in dehiscence-type defects. A dog model. Int J Periodontics Restorative Dent. 2009 Aug;29(4):425-33. PubMed PMID: 19639063.
Reason: Animal study

Al-Hezaimi K, Al-Askar M, Al-Fahad H, Al-Rasheed A, Al-Sourani N, Griffin T, O'Neill R, Javed F. Effect of enamel matrix derivative protein on the healing of standardized epithelial wounds: a histomorphometric analysis in vivo. Int Wound J. 2012 Aug;9(4):436-41. doi: 10.1111/j.1742-481X.2011.00904.x. Epub 2011 Dec 19. PubMed PMID: 22182231.
Reason: Autologous bone wasn't used

Alkan EA, Tüter G, Parlar A, Yücel A, Kurtiş B. Evaluation of peri-implant crevicular fluid prostaglandin E(2) levels in augmented extraction sockets by different biomaterials. Acta Odontol Scand. 2016 Oct;74(7):532-538. Epub 2016 Aug 22. PubMed PMID: 27546095.
Reason: Not related to the clinical question

Al Machot E, Hoffmann T, Lorenz K, Khalili I, Noack B. Clinical outcomes after treatment of periodontal intrabony defects with nanocrystalline hydroxyapatite (Ostim) or enamel matrix derivatives (Emdogain): a randomized controlled clinical trial. Biomed Res Int. 2014;2014:786353. doi: 10.1155/2014/786353. Epub 2014 Feb 9. PubMed PMID: 24689056; PubMed Central PMCID: PMC3932837.
Reason: Biomaterial different from Autologous bone

Amin HD, Olsen I, Knowles JC, Dard M, Donos N. Effects of enamel matrix proteins on multi-lineage differentiation of periodontal ligament cells in vitro. Acta Biomater. 2013 Jan;9(1):4796-805. doi: 10.1016/j.actbio.2012.09.008. Epub 2012 Sep 14. PubMed PMID: 22985741.
Reason: Autologous bone wasn't used

Amin HD, Olsen I, Knowles JC, Donos N. Differential effect of amelogenin peptides on osteogenic differentiation in vitro: identification of possible new drugs for bone repair and regeneration. Tissue Eng Part A. 2012 Jun;18(11-12):1193-202. doi: 10.1089/ten.TEA.2011.0375. Epub 2012 Mar 28. PubMed PMID: 22320389.
Reason: Study in vitro without Autologous bone

Apicella A, Heunemann P, Dejace L, Marascio M, Plummer CJG, Fischer P. Scaffold requirements for periodontal regeneration with enamel matrix derivative proteins. Colloids Surf B Biointerfaces. 2017 Aug 1;156:221-226. doi: 10.1016/j.colsurfb.2017.05.013. Epub 2017 May 9. PubMed PMID: 28531879.
Reason: Autologous bone wasn't used

Araújo MG, Lindhe J. GTR treatment of degree III furcation defects following application of enamel matrix proteins. An experimental study in dogs. J Clin Periodontol. 1998 Jun;25(6):524-30. PubMed PMID: 9667487.
Reason: Animal study

Artzi Z, Sudri S, Platner O, Kozlovsky A. Regeneration of the Periodontal Apparatus in Aggressive Periodontitis Patients. Dent J (Basel). 2019 Mar 8;7(1). pii: E29. doi: 10.3390/dj7010029. Review. PubMed PMID: 30857253.
Reason: Autologous bone wasn't used

Artzi Z, Tal H, Platner O, Wasersprung N, Weinberg E, Slutzkey S, Gozali N, Carmeli G, Herzberg R, Kozlovsky A. Deproteinized bovine bone in association with guided tissue regeneration or enamel matrix derivatives procedures in aggressive periodontitis patients: a 1-year retrospective study. J Clin Periodontol. 2015 Jun;42(6):547-56. doi: 10.1111/jcpe.12413. Epub 2015 May 30. PubMed PMID: 25950086.
Reason: Biomaterial different from Autologous bone

Aslan S, Buduneli N, Cortellini P. Entire Papilla Preservation Technique: A Novel Surgical Approach for Regenerative Treatment of Deep and Wide Intrabony Defects. Int J Periodontics Restorative Dent. 2017 Mar/Apr;37(2):227-233. doi: 10.11607/prd.2584. PubMed PMID: 28196163.
Reason: Autologous bone wasn't used

Aslan S, Buduneli N, Cortellini P. Entire papilla preservation technique in the regenerative treatment of deep intrabony defects: 1-Year results. J Clin Periodontol. 2017 Sep;44(9):926-932. doi: 10.1111/jcpe.12780. Epub 2017 Aug 23. PubMed PMID: 28727170.
Reason: Not related to the clinical question

Aspriello SD, Ferrante L, Rubini C, Piemontese M. Comparative study of DFDBA in combination with enamel matrix derivative versus DFDBA alone for treatment of periodontal intrabony defects at 12 months post-surgery. Clin Oral Investig. 2011 Apr;15(2):225-32. doi: 10.1007/s00784-009-0369-y. Epub 2010 Jan 7. PubMed PMID: 20054593.
Reason: Biomaterial used different from Autologous bone

Athanassiou-Papaefthymiou M, Papagerakis P, Papagerakis S. Isolation and Characterization of Human Adult Epithelial Stem Cells from the Periodontal Ligament. J Dent Res. 2015 Nov;94(11):1591-600. doi: 10.1177/0022034515606401. Epub 2015 Sep 21. PubMed PMID: 26392003.
Reason: Not related to the clinical question

Aydemir Turkal H, Demirer S, Dolgun A, Keceli HG. Evaluation of the adjunctive effect of platelet-rich fibrin to enamel matrix derivative in the treatment of intrabony defects. Six-month results of a randomized, split-mouth, controlled clinical study. J Clin Periodontol. 2016 Nov;43(11):955-964. doi: 10.1111/jcpe.12598. Epub 2016 Sep 13. PubMed PMID: 27396428.
Reason: Autologous bone wasn't used

Azaripour A, Willershausen I, Kämmerer P, Willershausen B. Post-endodontic treatment periodontal surgery: a case report. Quintessence Int. 2013 Feb;44(2):123-6. doi: 10.3290/j.qi.a28931. PubMed PMID: 23444178.
Reason: Not related to the clinical question

Azim AA, Lloyd A, Huang GT. Management of longstanding furcation perforation using a novel approach. J Endod. 2014 Aug;40(8):1255-9. doi: 10.1016/j.joen.2013.12.013. Epub 2014 Jan 17. PubMed PMID: 25069944.
Reason: Autologous bone wasn't used

Azzi R, Etienne D, Takei H, Carranza F. Bone regeneration using the pouch-and-tunnel technique. Int J Periodontics Restorative Dent. 2009 Oct;29(5):515-21. PubMed PMID: 19888495.
Reason: Not related to the clinical question

Behdin S, Monje A, Lin GH, Edwards B, Othman A, Wang HL. Effectiveness of Laser Application for Periodontal Surgical Therapy: Systematic Review and Meta-Analysis. J Periodontol. 2015 Dec;86(12):1352-63. doi: 10.1902/jop.2015.150212. Epub 2015 Aug 13. Review. PubMed PMID: 26269936.
Reason: Not related to the clinical question

Baltacioglu E, Tasdemir T, Yuva P, Celik D, Sukuroglu E. Intentional replantation of periodontally hopeless teeth using a combination of enamel matrix derivative and demineralized freeze-dried bone allograft. Int J Periodontics Restorative Dent. 2011 Feb;31(1):75-81. PubMed PMID: 21365029.
Reason: Biomaterial different from autologous bone

Barrett EJ, Kenny DJ. Optimization of post-replantation healing for avulsed permanent teeth in children. Ont Dent. 1999 Oct;76(8):23-7. PubMed PMID: 10850271.
Reason: Not related to the clinical question

Barrett EJ, Kenny DJ, Tenenbaum HC, Sigal MJ, Johnston DH. Replantation of permanent incisors in children using Emdogain. Dent Traumatol. 2005 Oct;21(5):269-75. PubMed PMID: 16149922.
Reason: Not related to the clinical question

Berlucchi I, Francetti L, Del Fabbro M, Basso M, Weinstein RL. The influence of anatomical features on the outcome of gingival recessions treated with coronally advanced flap and enamel matrix derivative: a 1-year prospective study. J Periodontol. 2005 Jun;76(6):899-907. PubMed PMID: 15948683.
Reason: Autologous Bone wasn't used

Bertoldi C, Ferrari M, Giannetti L. The use of only enamel matrix derivative allows outstanding regeneration results in periodontal intrabony defect treatment: a retrospective study. J Biol Regul Homeost Agents. 2019 Mar-Apr,;33(2):633-636. PubMed PMID: 30919609.
Reason: Autologous Bone wasn't used

Bertoldi C, Pellacani C, Lalla M, Consolo U, Pinti M, Cortellini P, Cossarizza A. Herpes Simplex I virus impairs regenerative outcomes of periodontal regenerative therapy in intrabony defects: a pilot study. J Clin Periodontol. 2012 Apr;39(4):385-92. doi: 10.1111/j.1600-051X.2012.01850.x. Epub 2012 Feb 1. PubMed PMID: 22292785.
Reason: Not related to the clinical question

Bhatavadekar NB, Paquette DW. Long-term follow-up and tomographic assessment of an intrabony defect treated with enamel matrix derivative. J Periodontol. 2008 Sep;79(9):1802-8. doi: 10.1902/jop.2008.070636 . PubMed PMID: 18771385.
Reason: Autologous Bone wasn't used

Bhutda G, Deo V. Five years clinical results following treatment of human intra-bony defects with an enamel matrix derivative: a randomized controlled trial. Acta Odontol Scand. 2013 May-Jul;71(3-4):764-70. doi: 10.3109/00016357.2012.728245. Epub 2012 Oct 19. PubMed PMID: 23078573.
Reason: Autologous Bone wasn't used

Bizenjima T, Osuka Y, Tomita S, Saito A. Periodontal Regenerative Therapy with Enamel Matrix Derivative in Patient with Chronic Periodontitis: A 3.5-year Follow-up Report. Bull Tokyo Dent Coll. 2019 Mar 15. doi: 10.2209/tdcpublication.2018-0048. [Epub ahead of print] PubMed PMID: 30880299.
Bokan I, Bill JS, Schlagenhauf U. Primary flap closure combined with Emdogain alone or Emdogain and Cerasorb in the treatment of intra-bony defects. J Clin Periodontol. 2006 Dec;33(12):885-93. PubMed PMID: 17092241.
Reason: Autologous Bone wasn't used

Bonnet N, Lesclous P, Saffar JL, Ferrari S. Zoledronate effects on systemic and jaw osteopenias in ovariectomized periostin-deficient mice. PLoS One. 2013;8(3):e58726. doi: 10.1371/journal.pone.0058726. Epub 2013 Mar 7. PubMed PMID: 23505553; PubMed Central PMCID: PMC3591374.
Reason: Not related to the clinical question

Bonta H, Llambes F, Moretti AJ, Mathur H, Bouwsma OJ. The use of enamel matrix protein in the treatment of localized aggressive periodontitis: a case report. Quintessence Int. 2003 Apr;34(4):247-52. PubMed PMID: 12731609.
Reason: Autologous Bone wasn't used

Bosshardt DD. Biological mediators and periodontal regeneration: a review of enamel matrix proteins at the cellular and molecular levels. J Clin Periodontol. 2008 Sep;35(8 Suppl):87-105. doi: 10.1111/j.1600-051X.2008.01264.x. Review. PubMed PMID: 18724844.
Reason: Autologous bone wasn't used

Bosshardt DD. Are cementoblasts a subpopulation of osteoblasts or a unique phenotype? J Dent Res. 2005 May;84(5):390-406. Review. PubMed PMID: 15840773.
Reason: Not related to the clinical question

Bosshardt DD, Sculean A, Donos N, Lang NP. Pattern of mineralization after regenerative periodontal therapy with enamel matrix proteins. Eur J Oral Sci. 2006 May;114 Suppl 1:225-31; discussion 254-6, 381-2. PubMed PMID: 16674690.
Reason: Autologous Bone wasn't used

Bosshardt DD, Sculean A, Windisch P, Pjetursson BE, Lang NP. Effects of enamel matrix proteins on tissue formation along the roots of human teeth. J Periodontal Res. 2005 Apr;40(2):158-67. PubMed PMID: 15733151.
Reason: Autologous Bone wasn't used

Boyan BD, Weesner TC, Lohmann CH, Andreacchio D, Carnes DL, Dean DD, Cochran DL, Schwartz Z. Porcine fetal enamel matrix derivative enhances bone formation induced by demineralized freeze dried bone allograft in vivo. J Periodontol. 2000 Aug;71(8):1278-86. PubMed PMID: 10972643.
Reason: Biomaterial different from Autologous Bone

Bratthall G, Lindberg P, Havemose-Poulsen A, Holmstrup P, Bay L, Söderholm G, Norderyd O, Andersson B, Rickardsson B, Hallström H, Kullendorff B, Sköld Bell H. Comparison of ready-to-use EMDOGAIN-gel and EMDOGAIN in patients with chronic adult periodontitis. J Clin Periodontol. 2001 Oct;28(10):923-9. PubMed PMID: 11686810.
Reason: Autologous Bone wasn't used

Bröseler F, Tietmann C, Hinz AK, Jepsen S. Long-term results of periodontal regenerative therapy: A retrospective practice-based cohort study. J Clin Periodontol. 2017 May;44(5):520-529. doi: 10.1111/jcpe.12723. Epub 2017 Apr 27.

PubMed PMID: 28303584.
Reason: Autologous Bone wasn't used

Caffesse RG, de la Rosa M, Mota LF. Regeneration of soft and hard tissue periodontal defects. Am J Dent. 2002 Oct;15(5):339-45. Review. PubMed PMID: 12537347.
Reason: Not related to the clinical question

Caffesse RG, Nasjleti CE, Morrison EC, Sanchez R. Guided tissue regeneration: comparison of bioabsorbable and non-bioabsorbable membranes. Histologic and histometric study in dogs. J Periodontol. 1994 Jun;65(6):583-91. PubMed PMID: 8083790.
Reason: Not related to the clinical question

Caglar E, Tanboga I, Süsal S. Treatment of avulsed teeth with Emdogain--a case report. Dent Traumatol. 2005 Feb;21(1):51-3. PubMed PMID: 15660758.
Reason: Autologous Bone wasn't used

Camargo PM, Lekovic V, Weinlaender M, Vasilic N, Kenney EB, Madzarevic M. The effectiveness of enamel matrix proteins used in combination with bovine porous bone mineral in the treatment of intrabony defects in humans. J Clin Periodontol. 2001 Nov;28(11):1016-22. PubMed PMID: 11686822.
Reason: Biomaterial different from Autologous Bone

Camelo M, Nevins ML, Schenk RK, Lynch SE, Nevins M. Periodontal regeneration in human Class II furcations using purified recombinant human platelet-derived growth factor-BB (rhPDGF-BB) with bone allograft. Int J Periodontics Restorative Dent. 2003 Jun;23(3):213-25. PubMed PMID: 12854772.
Reason: Not related to the clinical question

Cardaropoli G, Leonhardt AS. Enamel matrix proteins in the treatment of deep intrabony defects. J Periodontol. 2002 May;73(5):501-4. PubMed PMID: 12027251.
Reason: Autologous Bone wasn't used

Carinci F, Piattelli A, Guida L, Perrotti V, Laino G, Oliva A, Annunziata M, Palmieri A, Pezzetti F. Effects of Emdogain on osteoblast gene expression. Oral Dis. 2006 May;12(3):329-42. PubMed PMID: 16700745.
Reason: Autologous Bone wasn't used

Carnio J, Camargo PM, Kenney EB, Schenk RK. Histological evaluation of 4 cases of root coverage following a connective tissue graft combined with an enamel matrix derivative preparation. J Periodontol. 2002 Dec;73(12):1534-43. PubMed PMID: 12546106.
Reason: Autologous Bone wasn't used

Casarin RC, Ribeiro Edel P, Nociti FH Jr, Sallum AW, Ambrosano GM, Sallum EA, Casati MZ. Enamel matrix derivative proteins for the treatment of proximal class II furcation involvements: a prospective 24-month randomized clinical trial. J Clin Periodontol. 2010 Dec;37(12):1100-9. doi: 10.1111/j.1600-051X.2010.01614.x. Epub 2010 Aug 24. PubMed PMID: 20735795.
Reason: Autologous Bone wasn't used

Casarin RC, Ribeiro Edel P, Ribeiro FV, Nociti FH Jr, Sallum AW, Sallum EA, Casati MZ. Influence of anatomic features on the effectiveness of enamel matrix derivative proteins in the treatment of proximal Class II furcation involvements. Quintessence Int. 2009 Oct;40(9):753-61. PubMed PMID: 19862402.
Reason: Autologous Bone wasn't used

Casati MZ, Sallum EA, Nociti FH Jr, Caffesse RG, Sallum AW. Enamel matrix derivative and bone healing after guided bone regeneration in dehiscence-type defects around implants. A histomorphometric study in dogs. J Periodontol. 2002 Jul;73(7):789-96. PubMed PMID: 12146539.
Reason: Animal study

Chambrone D, Pasin IM, Chambrone L, Pannuti CM, Conde MC, Lima LA. Treatment of infrabony defects with or without enamel matrix proteins: a 24-month follow-up randomized pilot study. Quintessence Int. 2010 Feb;41(2):125-34. PubMed PMID: 20165744.
Reason: Autologous bone wasn't used

Chambrone D, Pasin IM, Conde MC, Panutti C, Carneiro S, Lima LA. Effect of enamel matrix proteins on the treatment of intrabony defects: a split-mouth randomized controlled trial study. Braz Oral Res. 2007 Jul-Sep;21(3):241-6.

PubMed PMID: 17710290.
Reason: Autologous bone wasn't used
Chambrone L, Sukekava F, Araújo MG, Pustiglioni FE, Chambrone LA, Lima LA. Root coverage procedures for the treatment of localised recession-type defects. Cochrane Database Syst Rev. 2009 Apr 15;(2):CD007161. doi: 10.1002/14651858.CD007161.pub2. Review. Update in: Cochrane Database Syst Rev. 2018 Oct 02;10:CD007161. PubMed PMID: 19370675.
Reason: not related to the clinical question

Chambrone L, Sukekava F, Araújo MG, Pustiglioni FE, Chambrone LA, Lima LA. Root-coverage procedures for the treatment of localized recession-type defects: a Cochrane systematic review. J Periodontol. 2010 Apr;81(4):452-78. doi: 10.1902/jop.2010.090540. Review. PubMed PMID: 20367089.
Reason: not related to the clinical question

Chano L, Tenenbaum HC, Lekic PC, Sodek J, McCulloch CA. Emdogain regulation of cellular differentiation in wounded rat periodontium. J Periodontal Res. 2003 Apr;38(2):164-74. PubMed PMID: 12608911.
Reason: Animal study

Chen L, Cha J, Ho CH. A three-point-translation technique for root coverage with 4-year follow-up. Dent Today. 2002 Oct;21(10):112-5. PubMed PMID: 12382500.
Reason: not related to the clinical question

Chen FM, Zhang J, Zhang M, An Y, Chen F, Wu ZF. A review on endogenous regenerative technology in periodontal regenerative medicine. Biomaterials. 2010 Nov;31(31):7892-927. doi: 10.1016/j.biomaterials.2010.07.019. Epub 2010 Aug 4. Review. PubMed PMID: 20684986.
Reason: not related to the clinical question

Chitsazi MT, Mostofi Zadeh Farahani R, Pourabbas M, Bahaeddin N. Efficacy of open flap debridement with and without enamel matrix derivatives in the treatment of mandibular degree II furcation involvement. Clin Oral Investig. 2007 Dec;11(4):385-9. Epub 2007 Jul 11. PubMed PMID: 17623113.
Reason: Autologous bone wasn't used

Cho AR, Kim JH, Lee DE, Lee JS, Jung UW, Bak EJ, Yoo YJ, Chung WG, Choi SH. The effect of orally administered epigallocatechin-3-gallate on ligature-induced periodontitis in rats. J Periodontal Res. 2013 Dec;48(6):781-9. doi: 10.1111/jre.12071. Epub 2013 Apr 15. PubMed PMID: 23581513.
Reason: not related to the clinical question

Chong CH, Carnes DL, Moritz AJ, Oates T, Ryu OH, Simmer J, Cochran DL. Human periodontal fibroblast response to enamel matrix derivative, amelogenin, and platelet-derived growth factor-BB. J Periodontol. 2006 Jul;77(7):1242-52. PubMed PMID: 16805689.
Reason: Autologous bone wasn't used

Cochran DL, Jones A, Heijl L, Mellonig JT, Schoolfield J, King GN. Periodontal regeneration with a combination of enamel matrix proteins and autogenous bone grafting. J Periodontol. 2003 Sep;74(9):1269-81. PubMed PMID: 14584859.
Reason: Animal study

Cochran DL, King GN, Schoolfield J, Velasquez-Plata D, Mellonig JT, Jones A. The effect of enamel matrix proteins on periodontal regeneration as determined by histological analyses. J Periodontol. 2003 Jul;74(7):1043-55. PubMed PMID: 12931768.
Reason: Autologous bone wasn't used

Cochran DL, Wozney JM. Biological mediators for periodontal regeneration. Periodontol 2000. 1999 Feb;19:40-58. Review. PubMed PMID: 10321215.
Reason: Not related to the clinical question

Corbella S, Alberti A, Calciolari E, Taschieri S, Francetti L. Enamel matrix derivative for the treatment of partially contained intrabony defects: 12-month results. Aust Dent J. 2019 Mar;64(1):27-34. doi: 10.1111/adj.12654. Epub 2018 Oct 15. PubMed PMID: 30257036.
Reason: Autologous bone wasn't used

Corrêa MG, Campos ML, Benatti BB, Marques MR, Casati MZ, Nociti FH Jr, Sallum EA. The impact of cigarette smoke inhalation on the outcome of enamel matrix derivative treatment in rats: histometric analysis. J Periodontol. 2010 Dec;81(12):1820-8. doi: 10.1902/jop.2010.100200. Epub 2010 Jul 14. PubMed PMID: 20629543.

Reason: Animal study not related to the clinical question

Corrêa MG, Gomes Campos ML, Marques MR, Ambrosano GM, Casati MZ, Nociti FH Jr, Sallum EA. Alcohol intake may impair bone density and new cementum formation after enamel matrix derivative treatment: histometric study in rats. J Periodontal Res. 2016 Feb;51(1):60-9. doi: 10.1111/jre.12279. Epub 2015 May 9. PubMed PMID: 25959998.
Reason: Animal study not related to the clinical question

Corrêa MG, Gomes Campos ML, Marques MR, Bovi Ambrosano GM, Casati MZ, Nociti FH Jr, Sallum EA. Outcome of enamel matrix derivative treatment in the presence of chronic stress: histometric study in rats. J Periodontol. 2014 Jul;85(7):e259-67. doi: 10.1902/jop.2013.130383. Epub 2013 Nov 28. PubMed PMID: 24283657.
Reason: Autologous bone wasn't used

Corrêa MG, Gomes Campos ML, Marques MR, Casati MZ, Nociti FH Jr, Sallum EA. Histometric analysis of the effect of enamel matrix derivative on the healing of periodontal defects in rats with diabetes. J Periodontol. 2013 Sep;84(9):1309-18. doi: 10.1902/jop.2012.120354. Epub 2012 Nov 3. PubMed PMID: 23121457.
Reason: Animal study not related to the clinical question

Cortellini P. Minimally invasive surgical techniques in periodontal regeneration. J Evid Based Dent Pract. 2012 Sep;12(3 Suppl):89-100. doi: 10.1016/S1532-3382(12)70021-0. Review. Pub Med PMID: 23040341.
Reason: not related to the clinical question

Cortellini P, Nieri M, Prato GP, Tonetti MS. Single minimally invasive surgical technique with an enamel matrix derivative to treat multiple adjacent intra-bony defects: clinical outcomes and patient morbidity. J Clin Periodontol. 2008 Jul;35(7):605-13. doi: 10.1111/j.1600-051X.2008.01242.x. Epub 2008 May 11. PubMed PMID: 18476997.
Reason: Autologous bone wasn't used

Cortellini P, Pini-Prato G, Nieri M, Tonetti MS. Minimally invasive surgical technique and enamel matrix derivative in intrabony defects: 2. Factors associated with healing outcomes. Int J Periodontics Restorative Dent. 2009 Jun;29(3):257-65. PubMed PMID: 19537465.
Reason: Autologous bone wasn't used

Cortellini P, Stalpers G, Mollo A, Tonetti MS. Periodontal regeneration versus extraction and prosthetic replacement of teeth severely compromised by attachment loss to the apex: 5-year results of an ongoing randomized clinical trial. J Clin Periodontol. 2011 Oct;38(10):915-24. doi: 10.1111/j.1600-051X.2011.01768.x. Epub 2011 Jul 21. PubMed PMID: 21777268
Reason: Not related to the clinical question

Cortellini P, Tonetti MS. A minimally invasive surgical technique with an enamel matrix derivative in the regenerative treatment of intra-bony defects: a novel approach to limit morbidity. J Clin Periodontol. 2007 Jan;34(1):87-93. PubMed PMID: 17243998.
Reason: Autologous bone wasn't used

Cortellini P, Tonetti MS. Clinical and radiographic outcomes of the modified minimally invasive surgical technique with and without regenerative materials: a randomized-controlled trial in intra-bony defects. J Clin Periodontol. 2011 Apr;38(4):365-73. doi: 10.1111/j.1600-051X.2011.01705.x. Epub 2011 Feb 8. PubMed PMID: 21303402.
Reason: Not related to the clinical question

Cortellini P, Tonetti MS. Clinical performance of a regenerative strategy for intrabony defects: scientific evidence and clinical experience. J Periodontol. 2005 Mar;76(3):341-50. PubMed PMID: 15857066.
Reason: Not related to the clinical question

Cortellini P, Tonetti MS. Improved wound stability with a modified minimally invasive surgical technique in the regenerative treatment of isolated interdental intrabony defects. J Clin Periodontol. 2009 Feb;36(2):157-63. doi: 10.1111/j.1600-051X.2008.01352.x. PubMed PMID: 19207892.
Reason: Not related to the clinical question

Cortellini P, Tonetti MS. Minimally invasive surgical technique and enamel matrix derivative in intra-bony defects. I: Clinical outcomes and morbidity. J

Clin Periodontol. 2007 Dec;34(12):1082-8. Epub 2007 Oct 22. PubMed PMID: 17953696.
Reason: Autologous bone wasn't used

Corten EM, Schellekens PP, Oey PL, Hage JJ, Kerst A, Kon M. Function of the clavicular part of the pectoralis major muscle after transplantation of its sternocostal part. Ann Plast Surg. 2007 Apr;58(4):392-6. PubMed PMID: 17413881.
Reason: Not related to the clinical question

Costa PP, Alves LB, Souza SL, Grisi MF, Palioto DB, Taba M Jr, Novaes AB Jr. Root Coverage in Smokers with Acellular Dermal Matrix Graft and Enamel Matrix Derivative: A 12-Month Randomized Clinical Trial. Int J Periodontics Restorative Dent. 2016 Jul-Aug;36(4):525-31. doi: 10.11607/prd.2560. PubMed PMID: 27333010.
Reason: Not related to the clinical question

Craig RG, Kallur SP, Inoue M, Rosenberg PA, LeGeros RZ. Effect of enamel matrix proteins on the periodontal connective tissue-material interface after wound healing. J Biomed Mater Res A. 2004 Apr 1;69(1):180-7. PubMed PMID: 14999766.
Reason: Not related to the clinical question

Craig RG, Kamer AR, Kallur SP, Inoue M, Tarnow DP. Effects of periodontal cell grafts and enamel matrix proteins on the implant-connective tissue interface: a pilot study in the minipig. J Oral Implantol. 2006;32(5):228-36. PubMed PMID: 17069167.
Reason: Not related to the clinical question

Crea A, Dassatti L, Hoffmann O, Zafiropoulos GG, Deli G. Treatment of intrabony defects using guided tissue regeneration or enamel matrix derivative: a 3-year prospective randomized clinical study. J Periodontol. 2008 Dec;79(12):2281-9. doi: 10.1902/jop.2008.080135 . PubMed PMID: 19053918.
Reason: Autologous bone wasn't used

Darwish SS, Abd El Meguid SH, Wahba NA, Mohamed AA, Chrzanowski W, Abou Neel EA. Root maturation and dentin-pulp response to enamel matrix derivative in pulpotomized permanent teeth. J Tissue Eng. 2014 Feb 2;5:2041731414521707.doi: 10.1177/2041731414521707. eCollection 2014. PubMed PMID: 24551447; PubMed Central PMCID: PMC3924881.
Reason: Not related to the clinical question

De Leonardis D, Paolantonio M. Enamel matrix derivative, alone or associated with a synthetic bone substitute, in the treatment of 1- to 2-wall periodontal defects. J Periodontol. 2013 Apr;84(4):444-55. doi: 10.1902/jop.2012.110656. Epub 2012 May 21. PubMed PMID: 22612371.
Reason: Biomaterial different from Autologous Bone

Del Fabbro M, Karanxha L, Panda S, Bucchi C, Nadathur Doraiswamy J, Sankari M, Ramamoorthi S, Varghese S, Taschieri S. Autologous platelet concentrates for treating periodontal infrabony defects. Cochrane Database Syst Rev. 2018 Nov 26;11:CD011423. doi: 10.1002/14651858.CD011423.pub2. PubMed PMID: 30484284.
Reason: Not related to the clinical question

de Sanctis M, Goracci C, Zucchelli G. Long-term effect on tooth vitality of regenerative therapy in deep periodontal bony defects: a retrospective study. Int J Periodontics Restorative Dent. 2013 Mar-Apr;33(2):151-7. doi: 10.11607/prd.1461. PubMed PMID: 23484170.
Reason: Not related to the clinical question

Deschner J, Eick S, Damanaki A, Nokhbehsaim M. The role of adipokines in periodontal infection and healing. Mol Oral Microbiol. 2014 Dec;29(6):258-69. doi: 10.1111/omi.12070. Epub 2014 Sep 27. Review. PubMed PMID: 25052571.
Reason: Not related to the clinical question

Deschner J, Nokhbehsaim M. Regulatory effects of inflammatory and biomechanical signals on regenerative periodontal healing. Int J Oral Maxillofac Implants. 2013 Nov-Dec;28(6):e472-7. doi: 10.11607/jomi.te27. PubMed PMID: 24278945.
Reason: Not related to the clinical question

D'Haese J, Dombret B, Verelst K, Wyn I, Matthijs S, Moradi M. [The armamentarium of the periodontologist]. Rev Belge Med Dent (1984). 2003;58(3):145-71. Review. French. PubMed PMID: 14686143.
Reason: Not related to the clinical question

Dickerman RD, Guyer R, Hisey M. Automated intraoperative EMG testing during percutaneous pedicle screw placement. Spine J. 2006 Jul-Aug;6(4):473. PubMed PMID: 16825058.
Reason: Not related to the clinical question

Diedrich P, Fritz U, Kinzinger G, Angelakis J. Movement of periodontally affected teeth after guided tissue regeneration (GTR)--an experimental pilot study in animals. J Orofac Orthop. 2003 May;64(3):214-27. PubMed PMID: 12835893.
Reason: Animal study

Dilsiz A, Canakci V, Aydin T. The combined use of Nd:YAG laser and enamel matrix proteins in the treatment of periodontal infrabony defects. J Periodontol. 2010 Oct;81(10):1411-8. doi: 10.1902/jop.2010.100031. PubMed PMID: 20528697.
Reason: Not related to the clinical question

Ding Y, Cao DS, Huang X, Xie J, Li H. Segmental Latissimus Dorsi Free Flap Attempting to Preserve Function at the Donor Site: Anatomical and Clinical Experiences. J Reconstr Microsurg. 2017 May;33(4):268-274. doi: 10.1055/s-0036-1597835. Epub 2017 Jan 28. PubMed PMID: 28131103.
Reason: Not related to the clinical question

Di Tullio M, Femminella B, Pilloni A, Romano L, D'Arcangelo C, De Ninis P, Paolantonio M. Treatment of supra-alveolar-type defects by a simplified papilla preservation technique for access flap surgery with or without enamel matrix proteins. J Periodontol. 2013 Aug;84(8):1100-10. doi: 10.1902/jop.2012.120075. Epub 2012 Oct 2. PubMed PMID: 23030240.
Reason: Not related to the clinical question

Dobbs WE. EMDOGAIN. Northwest Dent. 1999 Sep-Oct;78(5):27-9. PubMed PMID: 10823061.
Reason: Not related to the clinical question

Donos N, Bosshardt D, Lang N, Graziani F, Tonetti M, Karring T, Kostopoulos L. Bone formation by enamel matrix proteins and xenografts: an experimental study in the rat ramus. Clin Oral Implants Res. 2005 Apr;16(2):140-6. PubMed PMID: 15777322.
Reason: Animal study

Donos N, Glavind L, Karring T, Sculean A. Clinical evaluation of an enamel matrix derivative in the treatment of mandibular degree II furcation involvement: a 36-month case series. Int J Periodontics Restorative Dent. 2003 Oct;23(5):507-12. PubMed PMID: 14620125.
Reason: Autologous bone wasn't used

Donos N, Sculean A, Glavind L, Reich E, Karring T. Wound healing of degree III furcation involvements following guided tissue regeneration and/or Emdogain. A histologic study. J Clin Periodontol. 2003 Dec;30(12):1061-8. PubMed PMID: 15002892.
Reason: Not related to the clinical question

Dori F. [Effect of combined therapeutic methods on healing of periodontal vertical bone defects in regenerative surgery]. Orv Hetil. 2009 Mar 15;150(11):517-22. doi: 10.1556/OH.2009.28500. Hungarian. PubMed PMID: 19258248.
Reason: Not related to the clinical question

Döri F, Arweiler N, Gera I, Sculean A. Clinical evaluation of an enamel matrix protein derivative combined with either a natural bone mineral or beta-tricalcium phosphate. J Periodontol. 2005 Dec;76(12):2236-43. PubMed PMID: 16332235.
Reason: Biomaterials different from autologous bone

Döri F, Arweiler N, Húszár T, Gera I, Miron RJ, Sculean A. Five-year results evaluating the effects of platelet-rich plasma on the healing of intrabony defects treated with enamel matrix derivative and natural bone mineral. J Periodontol. 2013 Nov;84(11):1546-55. doi: 10.1902/jop.2013.120501. Epub 2013 Jan 17. PubMed PMID: 23327604.
Reason: Not related to the clinical question

Döri F, Arweiler NB, Szántó E, Agics A, Gera I, Sculean A. Ten-year results following treatment of intrabony defects with an enamel matrix protein derivative combined with either a natural bone mineral or a β-tricalcium phosphate. J Periodontol. 2013 Jun;84(6):749-57. doi: 10.1902/jop.2012.120238. Epub 2012 Aug

8. PubMed PMID: 22873657.
Reason: Biomaterials different from autologous bone

Döri F, Nikolidakis D, Húszár T, Arweiler NB, Gera I, Sculean A. Effect of platelet-rich plasma on the healing of intrabony defects treated with an enamel matrix protein derivative and a natural bone mineral. J Clin Periodontol. 2008 Jan;35(1):44-50. Epub 2007 Nov 21. PubMed PMID: 18034853.
Reason: Not related to the clinical question

Duailibi SE, Duailibi MT, Zhang W, Asrican R, Vacanti JP, Yelick PC. Bioengineered dental tissues grown in the rat jaw. J Dent Res. 2008 Aug;87(8):745-50. PubMed PMID: 18650546; PubMed Central PMCID: PMC3024580.
Reason: Animal study

Duan X, Tu Q, Zhang J, Ye J, Sommer C, Mostoslavsky G, Kaplan D, Yang P, Chen J. Application of induced pluripotent stem (iPS) cells in periodontal tissue regeneration. J Cell Physiol. 2011 Jan;226(1):150-7. doi: 10.1002/jcp.22316. PubMed PMID: 20658533; PubMed Central PMCID: PMC4137963.
Reason: Not related to the clinical question

Dubernard JM, Henry P, Parmentier H, Vallet B, Vial D, Badet L, Petruzzo P, Lefrançois N, Lanzetta M, Owen E, Hakim N. [First transplantation of two hands: results after 18 months]. Ann Chir. 2002 Jan;127(1):19-25. French. PubMed PMID: 11833300.
Reason: Not related to the clinical question

Eickholz P, Röllke L, Schacher B, Wohlfeil M, Dannewitz B, Kaltschmitt J, Krieger JK, Krigar DM, Reitmeir P, Kim TS. Enamel matrix derivative in propylene glycol alginate for treatment of infrabony defects with or without systemic doxycycline: 12- and 24-month results. J Periodontol. 2014 May;85(5):669-75. doi: 10.1902/jop.2013.130290. Epub 2013 Sep 24. PubMed PMID: 24059744.
Reason: Not related to the clinical question

El Helow K, El Askary Ael S. Regenerative barriers in immediate implant placement: a literature review. Implant Dent. 2008 Sep;17(3):360-71. doi: 10.1097/ID.0b013e3181813406. Review. PubMed PMID: 18784536.
Reason: Not related to the clinical question

Esposito M, Coulthard P, Thomsen P, Worthington HV. Enamel matrix derivative for periodontal tissue regeneration in treatment of intrabony defects: a Cochrane systematic review. J Dent Educ. 2004 Aug;68(8):834-44. Review. PubMed PMID: 15286106.
Reason: Autologous wasn't used

Esposito M, Coulthard P, Worthington HV. Enamel matrix derivative (Emdogain) for periodontal tissue regeneration in intrabony defects. Cochrane Database Syst Rev. 2003;(2):CD003875. Review. Update in: Cochrane Database Syst Rev. 2005;(4):CD003875. PubMed PMID: 12804493.
Reason: EMD evaluated alone

Esposito M, Grusovin MG, Coulthard P, Worthington HV. Enamel matrix derivative (Emdogain) for periodontal tissue regeneration in intrabony defects. Cochrane Database Syst Rev. 2005 Oct 19;(4):CD003875. Review. Update in: Cochrane Database Syst Rev. 2009;(4):CD003875. PubMed PMID: 16235343.
Reason: the effects of Autologous bone weren't evaluated
Reason: EMD evaluated alone

Esposito M, Grusovin MG, Papanikolaou N, Coulthard P, Worthington HV. Enamel matrix derivative (Emdogain) for periodontal tissue regeneration in intrabony defects. A Cochrane systematic review. Eur J Oral Implantol. 2009 Winter;2(4):247-66. Review. PubMed PMID: 20467602.
Reason: EMD used alone

Esposito M, Grusovin MG, Papanikolaou N, Coulthard P, Worthington HV. Enamel matrix derivative (Emdogain(R)) for periodontal tissue regeneration in intrabony defects. Cochrane Database Syst Rev. 2009 Oct 7;(4):CD003875. doi: 10.1002/14651858.CD003875.pub3. Review. PubMed PMID: 19821315.
Reason: EMD used alone

Fansa H, Plogmeier K, Feistner H, Schneider WJ. Plasticity and function--the fate of a free, neurovascular muscle graft ten years post-reconstruction. J Reconstr Microsurg. 1997 Nov;13(8):551-4. PubMed PMID: 9401982.
Reason: Not related to the clinical question

Farina R, Itro A, Ferrieri I, Trombelli L. Disease recurrence following reconstructive procedures: a 6- to 8-year follow-up observational study. Oral Health Prev Dent. 2007;5(4):307-12. PubMed PMID: 18173092.
Reason: Not related to the clinical question

Farina R, Simonelli A, Minenna L, Rasperini G, Schincaglia GP, Tomasi C, Trombelli L. Change in the Gingival Margin Profile After the Single Flap Approach in Periodontal Intraosseous Defects. J Periodontol. 2015 Sep;86(9):1038-46. doi: 10.1902/jop.2015.150040. Epub 2015 Apr 30. PubMed PMID: 25927424.
Reason: Not related to the clinical question

Farina R, Simonelli A, Minenna L, Rasperini G, Trombelli L. Single-flap approach in combination with enamel matrix derivative in the treatment of periodontal intraosseous defects. Int J Periodontics Restorative Dent. 2014 Jul-Aug;34(4):497-506. doi: 10.11607/prd.2050. PubMed PMID: 25006767.
Reason: EMD used alone

Farina R, Simonelli A, Rizzi A, Pramstraller M, Cucchi A, Trombelli L. Early postoperative healing following buccal single flap approach to access intraosseous periodontal defects. Clin Oral Investig. 2013 Jul;17(6):1573-83. doi: 10.1007/s00784-012-0838-6. Epub 2012 Sep 9. PubMed PMID: 22961538.
Fernandes JM, Rego RO, Spolidorio LC, Marcantonio RA, Marcantonio Júnior E, Cirelli JA. Enamel matrix proteins associated with GTR and bioactive glass in the treatment of class III furcation in dogs. Braz Oral Res. 2005 Jul-Sep;19(3):169-75. Epub 2005 Nov 21. PubMed PMID: 16308603.
Reason: Not related to the clinical question

Ferrarotti F, Romano F, Quirico A, Di Bella M, Pallotti S, Aimetti M. Effectiveness of Enamel Matrix Derivative in Conjunction with Particulate Autologous Bone in the Treatment of Noncontained Intrabony Defects: A 2-Year Prospective Case Series. Int J Periodontics Restorative Dent. 2018 September/October;38(5):673–680. doi: 10.11607/prd.3003. Epub 2018 Mar 7. PubMed PMID: 29513774.
Reason: Case series

Ferreira MM, Filomena BM, Lina C, Barbara O, Palmeirao CE. The effect of Emdogain gel on periodontal regeneration in autogenous transplanted dog's teeth. Indian J Dent Res. 2014 Sep-Oct;25(5):589-93. doi: 10.4103/0970-9290.147099. PubMed PMID: 25511057.
Reason: Animal study

Fickl S, Thalmair T, Kebschull M, Böhm S, Wachtel H. Microsurgical access flap in conjunction with enamel matrix derivative for the treatment of intra-bony defects: a controlled clinical trial. J Clin Periodontol. 2009 Sep;36(9):784-90. doi: 10.1111/j.1600-051X.2009.01451.x. Epub 2009 Jul 14. PubMed PMID: 19614721.
Reason: Autologous bone wasn't used

Filippi A, Pohl Y, von Arx T. Treatment of replacement resorption with Emdogain--a prospective clinical study. Dent Traumatol. 2002 Jun;18(3):138-43. PubMed PMID: 12154769.
Reason: Autologous bone wasn't used

Finkelstein T, Shapira Y, Bechor N, Shpack N. Surgical and orthodontic treatment of a fused maxillary central incisor and supernumerary tooth. J Clin Orthod. 2014 Oct;48(10):654-8. PubMed PMID: 25416343.
Reason: Not related to the clinical question

Forabosco A, Spinato S, Diacci S, Grippo A. [Clinical comparison between guided tissue regeneration and induced tissue regeneration]. Minerva Stomatol. 2003 Mar;52(3):105-10. Italian. PubMed PMID: 12783063.
Reason: Not related to the clinical question

Foster BL, Somerman MJ. Regenerating the periodontium: is there a magic formula? Orthod Craniofac Res. 2005 Nov;8(4):285-91. Review. PubMed PMID: 16238609.
Reason: Not related to the clinical question

Francetti L, Del Fabbro M, Basso M, Testori T, Weinstein R. Enamel matrix proteins in the treatment of intra-bony defects. A prospective 24-month clinical trial. J Clin Periodontol. 2004 Jan;31(1):52-9. PubMed PMID: 15058375.
Reason: Autologous bone wasn't used

Francetti L, Trombelli L, Lombardo G, Guida L, Cafiero C, Roccuzzo M, Carusi G, Del Fabbro M. Evaluation of efficacy of enamel matrix derivative in the treatment of intrabony defects: a 24-month multicenter study. Int J Periodontics

Restorative Dent. 2005 Oct;25(5):461-73. PubMed PMID: 16250568.
Reason: Autologous bone wasn't used
Franke Stenport V, Johansson CB. Enamel matrix derivative and titanium implants. J Clin Periodontol. 2003 Apr;30(4):359-63. PubMed PMID: 12694436.
Reason: Not related to the clinical question

Froum S, Lemler J, Horowitz R, Davidson B. The use of enamel matrix derivative in the treatment of periodontal osseous defects: a clinical decision tree based on biologic principles of regeneration. Int J Periodontics Restorative Dent. 2001 Oct;21(5):437-49. Review. PubMed PMID: 11693237.
Reason: Autologous bone wasn't used

Froum SJ, Froum SH, Rosen PS. A Regenerative Approach to the Successful Treatment of Peri-implantitis: A Consecutive Series of 170 Implants in 100 Patients with 2- to 10-Year Follow-up. Int J Periodontics Restorative Dent. 2015 Nov-Dec;35(6):857-63. doi: 10.11607/prd.2571. PubMed PMID: 26509990.
Reason: Not related to the clinical question

Froum SJ, Froum SH, Rosen PS. Successful management of peri-implantitis with a regenerative approach: a consecutive series of 51 treated implants with 3- to 7.5-year follow-up. Int J Periodontics Restorative Dent. 2012 Feb;32(1):11-20. PubMed PMID: 22254219.
Reason: Not related to the clinical question

Froum SJ, Weinberg MA, Rosenberg E, Tarnow D. A comparative study utilizing open flap debridement with and without enamel matrix derivative in the treatment of periodontal intrabony defects: a 12-month re-entry study. J Periodontol. 2001 Jan;72(1):25-34. PubMed PMID: 11210070.
Reason: Autologous bone wasn't used

Fueki K, Roumanas ED, Blackwell KE, Freymiller E, Abemayor E, Wong WK, Kapur KK, Garrett N. Effect of implant support for prostheses on electromyographic activity of masseter muscle and jaw movement in patients after mandibular fibula free flap reconstruction. Int J Oral Maxillofac Implants. 2014 Jan-Feb;29(1):162-70. doi: 10.11607/jomi.3197. PubMed PMID: 24451867
Reason: Not related to the clinical question

Fugazzotto PA. The clinical realities of mucogingival therapy. J Mass Dent Soc. 2005 Summer;54(2):24-7. PubMed PMID: 16149399.
Reason: Not related to the clinical question

Fujinami K, Hayakawa H, Ota K, Ida A, Nikaido M, Makiishi T, Saito A. Two-year follow-up of treatment of intrabony periodontal defect with enamel matrix derivative. Bull Tokyo Dent Coll. 2011;52(4):215-21. PubMed PMID: 22293592.
Reason: Autologous bone wasn't used

Fujishiro N, Anan H, Hamachi T, Maeda K. The role of macrophages in the periodontal regeneration using Emdogain gel. J Periodontal Res. 2008 Apr;43(2):143-55. doi: 10.1111/j.1600-0765.2007.01004.x. PubMed PMID: 18302615.
Reason: Not related to the clinical question

Fujita T, Yamamoto S, Ota M, Shibukawa Y, Yamada S. Coverage of gingival recession defects using guided tissue regeneration with and without adjunctive enamel matrix derivative in a dog model. Int J Periodontics Restorative Dent. 2011 Jun;31(3):247-53. PubMed PMID: 21556381.
Reason: Animal study

Fukae M, Kanazashi M, Nagano T, Tanabe T, Oida S, Gomi K. Porcine sheath proteins show periodontal ligament regeneration activity. Eur J Oral Sci. 2006 May;114 Suppl 1:212-8; discussion 254-6, 381-2. PubMed PMID: 16674688.
Reason: Not related to the clinical question

Fukuda T, Sanui T, Toyoda K, Tanaka U, Taketomi T, Uchiumi T, Nishimura F. Identification of novel amelogenin-binding proteins by proteomics analysis. PLoS One. 2013 Oct 22;8(10):e78129. doi: 10.1371/journal.pone.0078129. eCollection 2013. PubMed PMID: 24167599; PubMed Central PMCID: PMC3805512.
Reason: Not related to the clinical question

Fung K, Chandhoke TK, Uribe F, Schincaglia GP. Periodontal regeneration and orthodontic intrusion of a pathologically migrated central incisor adjacent to an infrabony defect. J Clin Orthod. 2012 Jul;46(7):417-23; quiz 438. PubMed PMID: 23059464.

Reason: Not related to the clinical question

Galli C, Macaluso GM, Guizzardi S, Vescovini R, Passeri M, Passeri G. Osteoprotegerin and receptor activator of nuclear factor-kappa B ligand modulation by enamel matrix derivative in human alveolar osteoblasts. J Periodontol. 2006 Jul;77(7):1223-8. PubMed PMID: 16805686.
Reason: Not related to the clinical question

Ghezzi C, Masiero S, Silvestri M, Zanotti G, Rasperini G. Orthodontic treatment of periodontally involved teeth after tissue regeneration. Int J Periodontics Restorative Dent. 2008 Dec;28(6):559-67. PubMed PMID: 19146051.
Reason: Not related to the clinical question

Gkranias ND, Graziani F, Sculean A, Donos N. Wound healing following regenerative procedures in furcation degree III defects: histomorphometric outcomes. Clin Oral Investig. 2012 Feb;16(1):239-49. doi: 10.1007/s00784-010-0478-7. Epub 2010 Oct 22. PubMed PMID: 20967476.
Grabosch A, Gutjahr L, Gruhl L, Bruck JC. [Electromyographic studies of myocutaneous sliding flaps for the covering of sacral decubitus ulcer]. Handchir Mikrochir Plast Chir. 1991 Nov;23(6):307-11. German. PubMed PMID: 1761246.
Reason: Autologous bone wasn't used

Giannobile WV, Hollister SJ, Ma PX. Future Prospects for Periodontal Bioengineering Using Growth Factors. Clin Adv Periodontics. 2011 Aug 1;1(2):88-94. PubMed PMID: 26500808; PubMed Central PMCID: PMC4617553.
Reason: Not related to the clinical question

Giannobile WV, Somerman MJ. Growth and amelogenin-like factors in periodontal wound healing. A systematic review. Ann Periodontol. 2003 Dec;8(1):193-204. Review. PubMed PMID: 14971254.
Reason: Autologous bone effect weren't evaluated

Gilio DA. Clinical efficacy of the Nd:YAG laser for combination therapy using EMD for periodontal reconstructive surgery: clinical case reports. Dent Today. 2001 Sep;20(9):106-11. PubMed PMID: 11569192.
Reason: Not related to the clinical question

Goda S, Inoue H, Kaneshita Y, Nagano Y, Ikeo T, Iida J, Domae N. Emdogain stimulates matrix degradation by osteoblasts. J Dent Res. 2008 Aug;87(8):782-7. Erratum in: J Dent Res. 2008 Oct;87(10):984.. Ikeo, Y T [corrected to Ikeo, T]. PubMed PMID: 18650553.
Reason: Autologous bone effect weren't evaluated

Goda S, Inoue H, Takeuchi O, Ujii Y, Domae E, Ikeo T. Enamel matrix derivative protein enhances production of matrixmetalloproteinase-2 by osteoblasts. BMC Oral Health. 2014 Jul 10;14:85.doi: 10.1186/1472-6831-14-85. PubMed PMID: 25011999; PubMed Central PMCID: PMC4115475.
Reason: Autologous bone effect weren't evaluated

Graziani F, Gennai S, Cei S, Ducci F, Discepoli N, Carmignani A, Tonetti M. Does enamel matrix derivative application provide additional clinical benefits in residual periodontal pockets associated with suprabony defects? A systematic review and meta-analysis of randomized clinical trials. J Clin Periodontol. 2014 Apr;41(4):377-86. doi: 10.1111/jcpe.12218. Epub 2014 Jan 22. Review. PubMed PMID: 24329867.
Reason: Autologous bone effect weren't evaluated

Greenstein G. Emdogain: evidence of efficacy. Compend Contin Educ Dent. 2000 Apr;21(4):299-305, 308, 310 passim; quiz 314. Review. PubMed PMID: 11199682.
Reason: Autologous bone effect weren't evaluated

Grigoriadis A, Johansson RS, Trulsson M. Adaptability of mastication in people with implant-supported bridges. J Clin Periodontol. 2011 Apr;38(4):395-404. doi: 10.1111/j.1600-051X.2010.01697.x. Epub 2011 Jan 12. PubMed PMID: 21226752.
Reason: Not related to the clinical question

Gruenbaum-Cohen Y, Tucker AS, Haze A, Shilo D, Taylor AL, Shay B, Sharpe PT, Mitsiadis TA, Ornoy A, Blumenfeld A, Deutsch D. Amelogenin in cranio-facial development: the tooth as a model to study the role of amelogenin during embryogenesis. J Exp Zool B Mol Dev Evol. 2009 Jul 15;312B(5):445-57. doi: 10.1002/jez.b.21255. Review. PubMed PMID: 19097165.
Reason: Not related to the clinical question

Grusovin MG, Esposito M. The efficacy of enamel matrix derivative (Emdogain) for the treatment of deep infrabony periodontal defects: a placebo-controlled randomised clinical trial. Eur J Oral Implantol. 2009 Spring;2(1):43-54. PubMed PMID: 20467617.
Reason: Autologous bone wasn't used

Guimarães GF, de Araújo VC, Nery JC, Peruzzo DC, Soares AB. Microvessel Density Evaluation of the Effect of Enamel Matrix Derivative on Soft Tissue After Implant Placement: A Preliminary Study. Int J Periodontics Restorative Dent. 2015 Sep-Oct;35(5):733-8. doi: 10.11607/prd.2044. PubMed PMID: 26357703.
Reason: Not related to the clinical question

Gungormus M, Oren EE, Horst JA, Fong H, Hnilova M, Somerman MJ, Snead ML, Samudrala R, Tamerler C, Sarikaya M. Cementomimetics-constructing a cementum-like biomineralized microlayer via amelogenin-derived peptides. Int J Oral Sci. 2012 Jun;4(2):69-77. doi: 10.1038/ijos.2012.40. Epub 2012 Jun 29. PubMed PMID: 22743342; PubMed Central PMCID: PMC3412665.
Reason: Not related to the clinical question

Guo Y, Guo W, Chen J, Chen G, Tian W, Bai D. Are Hertwig's epithelial root sheath cells necessary for periodontal formation by dental follicle cells? Arch Oral Biol. 2018 Oct;94:1-9. doi: 10.1016/j.archoralbio.2018.06.014. Epub 2018 Jun 18. PubMed PMID: 29929068.
Reason: Not related to the clinical question

Gupta SJ, Jhingran R, Gupta V, Bains VK, Madan R, Rizvi I. Efficacy of platelet-rich fibrin vs. enamel matrix derivative in the treatment of periodontal intrabony defects: a clinical and cone beam computed tomography study. J Int Acad Periodontol. 2014 Jul;16(3):86-96. PubMed PMID: 25654961.
Reason: Autologous bone effect weren't evaluated

Gurinsky BS, Mills MP, Mellonig JT. Clinical evaluation of demineralized freeze-dried bone allograft and enamel matrix derivative versus enamel matrix derivative alone for the treatment of periodontal osseous defects in humans. J Periodontol. 2004 Oct;75(10):1309-18. PubMed PMID: 15562907.
Reason: Biomaterial different from autologous bone

Hägewald S, Spahr A, Rompola E, Haller B, Heijl L, Bernimoulin JP. Comparative study of Emdogain and coronally advanced flap technique in the treatment of human gingival recessions. A prospective controlled clinical study. J Clin Periodontol. 2002 Jan;29(1):35-41. PubMed PMID: 11846847.
Reason: Autologous bone wasn't used

Hakki SS, Bozkurt SB, Türkay E, Dard M, Purali N, Götz W. Recombinant amelogenin regulates the bioactivity of mouse cementoblasts in vitro. Int J Oral Sci. 2018 May 9;10(2):15. doi: 10.1038/s41368-018-0010-5. PubMed PMID: 29748557; PubMed Central PMCID: PMC5966809.
Reason: Animal study

Hamamoto Y, Kawasaki N, Jarnbring F, Hammarström L. Effects and distribution of the enamel matrix derivative Emdogain in the periodontal tissues of rat molars transplanted to the abdominal wall. Dent Traumatol. 2002 Feb;18(1):12-23. PubMed PMID: 11841461.
Reason: Animal study

Hammarström L. The role of enamel matrix proteins in the development of cementum and periodontal tissues. Ciba Found Symp. 1997;205:246-55; discussion 255-60. Review. PubMed PMID: 9189629.
Reason: Autologous bone effects not evaluated

Hammarström L, Heijl L, Gestrelius S. Periodontal regeneration in a buccal dehiscence model in monkeys after application of enamel matrix proteins. J Clin Periodontol. 1997 Sep;24(9 Pt 2):669-77. PubMed PMID: 9310871.
Reason: Animal study

Harahashi H, Odajima T, Yamamoto T, Kawanami M. Immunohistochemical analysis of periodontal reattachment on denuded root dentin after periodontal surgery. Biomed Res. 2010 Oct;31(5):319-28. PubMed PMID: 21079362.
Reason: Not related to the clinical question

Harrel SK, Wilson TG, Nunn ME. Prospective assessment of the use of enamel matrix proteins with minimally invasive surgery. J Periodontol. 2005 Mar;76(3):380-4. PubMed PMID: 15857071.
Reason: Autologous bone effects not evaluated

Harrel SK, Wilson TG Jr, Nunn ME. Prospective assessment of the use of enamel matrix derivative with minimally invasive surgery: 6-year results. J Periodontol. 2010 Mar;81(3):435-41. doi: 10.1902/jop.2009.090393. PubMed PMID: 20192871.
Reason: Autologous bone effects not evaluated

Harris RJ, Harris LE, Harris CR, Harris AJ. Clinical evaluation of a combined regenerative technique with enamel matrix derivative, bone grafts, and guided tissue regeneration. Int J Periodontics Restorative Dent. 2007 Apr;27(2):171-9. PubMed PMID: 17514889.
Reason: Biomaterials different from Autologous bone

Haruyama N, Hatakeyama J, Moriyama K, Kulkarni AB. Amelogenins: Multi-Functional Enamel Matrix Proteins and Their Binding Partners. J Oral Biosci. 2011 Aug 1;53(3):257-266. PubMed PMID: 23914134; PubMed Central PMCID: PMC3732036.
Reason: Not related to the clinical question

Hasegawa N, Kawaguchi H, Ogawa T, Uchida T, Kurihara H. Immunohistochemical characteristics of epithelial cell rests of Malassez during cementum repair. J Periodontal Res. 2003 Feb;38(1):51-6. PubMed PMID: 12558937.
Reason: Not related to the clinical question

Hattar S, Asselin A, Greenspan D, Oboeuf M, Berdal A, Sautier JM. Potential of biomimetic surfaces to promote in vitro osteoblast-like cell differentiation. Biomaterials. 2005 Mar;26(8):839-48. PubMed PMID: 15353195.
Reason: Not related to the clinical question

Hayakawa H, Fujinami K, Ida A, Furusawa M, Nikaido M, Yamashita S, Saito A. Clinical outcome of surgical periodontal therapy: a short-term retrospective study. Bull Tokyo Dent Coll. 2012;53(4):189-95. PubMed PMID: 23318924.
Reason: Not related to the clinical question

Haze A, Taylor AL, Haegewald S, Leiser Y, Shay B, Rosenfeld E, Gruenbaum-Cohen Y, Dafni L, Zimmermann B, Heikinheimo K, Gibson CW, Fisher LW, Young MF, Blumenfeld A, Bernimoulin JP, Deutsch D. Regeneration of bone and periodontal ligament induced by recombinant amelogenin after periodontitis. J Cell Mol Med. 2009 Jun;13(6):1110-24. doi: 10.1111/j.1582-4934.2009.00700.x. Epub 2009 Feb 17. PubMed PMID: 19228267; PubMed Central PMCID: PMC2889159.
Reason: Autologous bone wasn't used

Heard RH, Mellonig JT, Brunsvold MA, Lasho DJ, Meffert RM, Cochran DL. Clinical evaluation of wound healing following multiple exposures to enamel matrix protein derivative in the treatment of intrabony periodontal defects. J Periodontol. 2000 Nov;71(11):1715-21. PubMed PMID: 11128919.
Reason: Autologous bone wasn't used

Heard RH, Mellonig JT. Regenerative materials: an overview. Alpha Omegan. 2000 Dec;93(4):51-8. Review. PubMed PMID: 11212411.
Reason: Autologous bone effects not evaluated

Heden G. A case report study of 72 consecutive Emdogain-treated intrabony periodontal defects: clinical and radiographic findings after 1 year. Int J Periodontics Restorative Dent. 2000 Apr;20(2):127-39. PubMed PMID: 11203555
Reason: Autologous bone wasn't used

Heden G, Wennström JL. Five-year follow-up of regenerative periodontal therapy with enamel matrix derivative at sites with angular bone defects. J Periodontol. 2006 Feb;77(2):295-301. PubMed PMID: 16460257.
Reason: Autologous bone wasn't used

Heden G, Wennström J, Lindhe J. Periodontal tissue alterations following Emdogain treatment of periodontal sites with angular bone defects. A series of case reports. J Clin Periodontol. 1999 Dec;26(12):855-60. PubMed PMID: 10599915.
Reason: Autologous bone wasn't used

Heijl L. Periodontal regeneration with enamel matrix derivative in one human experimental defect. A case report. J Clin Periodontol. 1997 Sep;24(9 Pt 2):693-6. PubMed PMID: 9310874.
Reason: Autologous bone wasn't used

Heijl L, Heden G, Svärdström G, Ostgren A. Enamel matrix derivative (EMDOGAIN) in the treatment of intrabony periodontal defects. J Clin Periodontol.

1997 Sep;24(9 Pt 2):705-14. PubMed PMID: 9310876.
Reason: Autologous bone wasn't used
Hirooka H. The biologic concept for the use of enamel matrix protein: true periodontal regeneration. Quintessence Int. 1998 Oct;29(10):621-30. Review. PubMed PMID: 9922759.
Reason: Autologous bone effects not evaluated

Hisanaga Y, Suzuki E, Aoki H, Sato M, Saito A, Saito A, Azuma T. Effect of the combined use of enamel matrix derivative and atelocollagen sponge scaffold on osteoblastic differentiation of mouse induced pluripotent stem cells in vitro. J Periodontal Res. 2018 Apr;53(2):240-249. doi: 10.1111/jre.12511. Epub 2017 Oct 17. PubMed PMID: 29044527.
Reason: Biomaterial different from Autologous bone

Hoffmann T, Al-Machot E, Meyle J, Jervøe-Storm PM, Jepsen S. Three-year results following regenerative periodontal surgery of advanced intrabony defects with enamel matrix derivative alone or combined with a synthetic bone graft. Clin Oral Investig. 2016 Mar;20(2):357-64. doi: 10.1007/s00784-015-1522-4. Epub 2015 Jul 15. PubMed PMID: 26174080.
Reason: Biomaterial different from Autologous bone

Hoffmann T, Richter S, Meyle J, Gonzales JR, Heinz B, Arjomand M, Sculean A, Reich E, Jepsen K, Jepsen S, Boedeker RH. A randomized clinical multicentre trial comparing enamel matrix derivative and membrane treatment of buccal class II furcation involvement in mandibular molars. Part III: patient factors and treatment outcome. J Clin Periodontol. 2006 Aug;33(8):575-83. PubMed PMID: 16899101.
Reason: Autologous bone effects not evaluated

Hoidal MJ, Grimard BA, Mills MP, Schoolfield JD, Mellonig JT, Mealey BL. Clinical evaluation of demineralized freeze-dried bone allograft with and without enamel matrix derivative for the treatment of periodontal osseous defects in humans. J Periodontol. 2008 Dec;79(12):2273-80. doi: 10.1902/jop.2008.080259 . PubMed PMID: 19053917.
Reason: Biomaterial different from Autologous bone

Horváth A, Gera I. [Salvage of a tooth with necrotised periodontium, caused by endodontic use of radiosurgery. Long-term results. Case report]. Fogorv Sz. 2013 Jun;106(2):71-7. Hungarian. PubMed PMID: 24344563.
Reason: Not related to the clinical question

Houshmand B, Behnia H, Khoshzaban A, Morad G, Behrouzi G, Dashti SG, Khojasteh A. Osteoblastic differentiation of human stem cells derived from bone marrow and periodontal ligament under the effect of enamel matrix derivative and transforming growth factor-beta. Int J Oral Maxillofac Implants. 2013 Nov-Dec;28(6):e440-50. doi: 10.11607/jomi.te24. PubMed PMID: 24278943.
Reason: Not related to the clinical question

Hovey LR, Jones AA, McGuire M, Mellonig JT, Schoolfield J, Cochran DL. Application of periodontal tissue engineering using enamel matrix derivative and a human fibroblast-derived dermal substitute to stimulate periodontal wound healing in Class III furcation defects. J Periodontol. 2006 May;77(5):790-9. PubMed PMID: 16671870.
Reason: Autologous bone wasn't used

Hürzeler MB, Zuhr O, Schupbach P, Rebele SF, Emmanouilidis N, Fickl S. The socket-shield technique: a proof-of-principle report. J Clin Periodontol. 2010 Sep;37(9):855-62. doi: 10.1111/j.1600-051X.2010.01595.x. PubMed PMID: 20712701.
Reason: Not related to the clinical question

Inoue M, LeGeros RZ, Hoffman C, Diamond K, Rosenberg PA, Craig RG. Effect of enamel matrix proteins on the phenotype expression of periodontal ligament cells cultured on dental materials. J Biomed Mater Res A. 2004 Apr 1;69(1):172-9. PubMed PMID: 14999765.
Reason: Not related to the clinical question

Iorio-Siciliano V, Andreuccetti G, Blasi A, Matarasso M, Sculean A, Salvi GE. Clinical outcomes following regenerative therapy of non-contained intrabony defects using a deproteinized bovine bone mineral combined with either enamel matrix derivative or collagen membrane. J Periodontol. 2014 Oct;85(10):1342-50. doi: 10.1902/jop.2014.130420. Epub 2014 May 16. PubMed PMID: 24835417.
Reason: Biomaterial different from autologous bone

Iqbal MK, Bamaas N. Effect of enamel matrix derivative (EMDOGAIN) upon

periodontal healing after replantation of permanent incisors in beagle dogs. Dent Traumatol. 2001 Feb;17(1):36-45. PubMed PMID: 11475769.
Reason: Animal study

Irokawa D, Makino-Oi A, Fujita T, Yamamoto S, Tomita S, Saito A. Adjunct Antimicrobial Therapy and Periodontal Surgery to Treat Generalized Aggressive Periodontitis: A Case Report. Bull Tokyo Dent Coll. 2016;57(2):105-14. doi: 10.2209/tdcpublication.2015-0040. PubMed PMID: 27320300.
Reason: Not related to the clinical question

Isehed C, Holmlund A, Renvert S, Svenson B, Johansson I, Lundberg P. Effectiveness of enamel matrix derivative on the clinical and microbiological outcomes following surgical regenerative treatment of peri-implantitis. A randomized controlled trial. J Clin Periodontol. 2016 Oct;43(10):863-73. doi: 10.1111/jcpe.12583. Epub 2016 Jul 15. PubMed PMID: 27418458.
Reason: Not related to the clinical question

Ishida S, Shibuya Y, Kobayashi M, Komori T. Assessing stomatognathic performance after mandibulectomy according to the method of mandibular reconstruction. Int J Oral Maxillofac Surg. 2015 Aug;44(8):948-55. doi: 10.1016/j.ijom.2015.03.011. Epub 2015 Apr 2. PubMed PMID: 25843536.
Reason: Not related to the clinical question

Ito K, Ito K, Owa M. Connective tissue grafting for root coverage in multiple Class III gingival recessions with enamel matrix derivative: a case report. Pract Periodontics Aesthet Dent. 2000 Jun-Jul;12(5):441-6; quiz 448. PubMed PMID: 11405001.
Reason: Case report without use of autologous bone

Itoh N, Kasai H, Ariyoshi W, Harada E, Yokota M, Nishihara T. Mechanisms involved in the enhancement of osteoclast formation by enamel matrix derivative. J Periodontal Res. 2006 Aug;41(4):273-9. PubMed PMID: 16827720.
Reason: Not related to the clinical question

Ivanovski S. Periodontal regeneration. Aust Dent J. 2009 Sep;54 Suppl 1:S118-28. doi: 10.1111/j.1834-7819.2009.01150.x. Review. PubMed PMID: 19737264.
Reason: Not related to the clinical question

Izumi Y, Aoki A, Yamada Y, Kobayashi H, Iwata T, Akizuki T, Suda T, Nakamura S, Wara-Aswapati N, Ueda M, Ishikawa I. Current and future periodontal tissue engineering. Periodontol 2000. 2011 Jun;56(1):166-87. doi: 10.1111/j.1600-0757.2010.00366.x. Review. PubMed PMID: 21501243.
Reason: Not related to the clinical question

Jacobs R, van Steenberghe D. Qualitative evaluation of the masseteric poststimulus EMG complex following mechanical or acoustic stimulation of osseointegrated oral implants. Int J Oral Maxillofac Implants. 1995 Mar-Apr;10(2):175-82. PubMed PMID: 7744436.
Reason: Not related to the clinical question

Jaiswal R, Deo V. Evaluation of the effectiveness of enamel matrix derivative, bone grafts, and membrane in the treatment of mandibular Class II furcation defects. Int J Periodontics Restorative Dent. 2013 Mar-Apr;33(2):e58-64. doi: 10.11607/prd.1428. PubMed PMID: 23484181.
Reason: Autologous bone effects weren't evaluated

Jankovic S, Aleksic Z, Milinkovic I, Dimitrijevic B. The coronally advanced flap in combination with platelet-rich fibrin (PRF) and enamel matrix derivative in the treatment of gingival recession: a comparative study. Eur J Esthet Dent. 2010 Autumn;5(3):260-73. PubMed PMID: 20820456.
Reason: Not related to the clinical question

Jang I, Lee JK, Song GS, Choi DS, Yozgatian JH, Cha BK. Application of Enamel Matrix Derivative and Intrusive Orthodontic Movement in the Treatment of Vertical Bony Defects: A Case Report. Int J Periodontics Restorative Dent. 2019 Jan/Feb;39(1):73-81. doi: 10.11607/prd.3432. PubMed PMID: 30543730.
Reason: Not related to the clinical question

Jepsen S, Heinz B, Jepsen K, Arjomand M, Hoffmann T, Richter S, Reich E, Sculean A, Gonzales JR, Bödeker RH, Meyle J. A randomized clinical trial comparing enamel matrix derivative and membrane treatment of buccal Class II furcation involvement in mandibular molars. Part I: Study design and results for primary outcomes. J Periodontol. 2004 Aug;75(8):1150-60. PubMed PMID: 15455745.

Reason: Not related to the clinical question

Jepsen S, Topoll H, Rengers H, Heinz B, Teich M, Hoffmann T, Al-Machot E, Meyle J, Jervøe-Storm PM. Clinical outcomes after treatment of intra-bony defects with an EMD/synthetic bone graft or EMD alone: a multicentre randomized-controlled clinical trial. J Clin Periodontol. 2008 May;35(5):420-8. doi: 10.1111/j.1600-051X.2008.01217.x. Epub 2008 Mar 12. PubMed PMID: 18341601.
Reason: Biomaterial different from autologous bone

Jiang S, Guo SJ, Chen JJ. [Research of induced pluripotent stem cells in oral tissue regeneration]. Zhonghua Kou Qiang Yi Xue Za Zhi. 2012 May;47(5):318-20. doi: 10.3760/cma.j.issn.1002-0098.2012.05.017. Review. Chinese. PubMed PMID: 22883832.
Reason: Not related to the clinical question

Jiang J, Safavi KE, Spangberg LS, Zhu Q. Enamel matrix derivative prolongs primary osteoblast growth. J Endod. 2001 Feb;27(2):110-2. PubMed PMID: 11491633.
Reason: Not related to the clinical question

Jiang SY, Shu R, Song ZC, Xie YF. Effects of enamel matrix proteins on proliferation, differentiation and attachment of human alveolar osteoblasts. Cell Prolif. 2011 Aug;44(4):372-9. doi: 10.1111/j.1365-2184.2011.00762.x. PubMed PMID: 21702859.
Reason: Not related to the clinical question

Jingchao H, Rong S, Zhongchen S, Lan C. Human amelogenin up-regulates osteogenic gene expression in human bone marrow stroma cells. Biochem Biophys Res Commun. 2011 May 13;408(3):437-41. doi: 10.1016/j.bbrc.2011.04.042. Epub 2011 Apr 13. PubMed PMID: 21514271.
Reason: Not related to the clinical question

Kakegawa A, Oida S, Gomi K, Nagano T, Yamakoshi Y, Fukui T, Kanazashi M, Arai T, Fukae M. Cytodifferentiation activity of synthetic human enamel sheath protein peptides. J Periodontal Res. 2010 Oct;45(5):643-9. doi: 10.1111/j.1600-0765.2010.01279.x. Epub 2010 Jun 20. PubMed PMID: 20572923.
Reason: Not related to the clinical question

Kalpidis CD, Ruben MP. Treatment of intrabony periodontal defects with enamel matrix derivative: a literature review. J Periodontol. 2002 Nov;73(11):1360-76. Review. PubMed PMID: 12479642.
Reason: Autologous bone effects weren't evaluated

Kamoi K, Iino M, Ishiguro H. Regeneration therapy for oral disease. Hum Cell. 2006 May;19(2):76-82. Review. PubMed PMID: 16879560.
Reason: Not related to the clinical question

Kanazashi M, Gomi K, Nagano T, Tanabe T, Arai T, Fukae M. The 17-kDa sheath protein in enamel proteins induces cementum regeneration in experimental cavities created in a buccal dehiscence model of dogs. J Periodontal Res. 2006 Jun;41(3):193-9. PubMed PMID: 16677288.
Reason: Animal study

Kaner D, Bernimoulin JP, Kleber BM, Friedmann A. Minimally invasive flap surgery and enamel matrix derivative in the treatment of localized aggressive periodontitis: case report. Int J Periodontics Restorative Dent. 2009 Feb;29(1):89-97. PubMed PMID: 19244886.
Reason: Not related to the clinical question

Kao RT, Conte G, Nishimine D, Dault S. Tissue engineering for periodontal regeneration. J Calif Dent Assoc. 2005 Mar;33(3):205-15. Review. PubMed PMID: 15918402.
Reason: Not related to the clinical question

Kao DW, Fiorellini JP. Regenerative periodontal therapy. Front Oral Biol. 2012;15:149-59. doi: 10.1159/000329677. Epub 2011 Nov 11. Review. PubMed PMID: 22142962.
Reason: Not related to the clinical question

Kao RT, Nares S, Reynolds MA. Periodontal regeneration - intrabony defects: a systematic review from the AAP Regeneration Workshop. J Periodontol. 2015 Feb;86(2 Suppl):S77-104. doi: 10.1902/jop.2015.130685. Epub 2014 Sep 12. Review. PubMed PMID: 25216204.
Reason: Not related to the clinical question

Kapferer I, Schmidt S, Gstir R, Durstberger G, Huber LA, Vietor I. Gene-expression profiles of epithelial cells treated with EMD in vitro: analysis using complementary DNA arrays. J Periodontal Res. 2011 Feb;46(1):118-25. doi: 10.1111/j.1600-0765.2010.01321.x. Epub 2010 Nov 26. PubMed PMID: 21108644.
Reason: Not related to the clinical question

Karring T. Regenerative periodontal therapy. J Int Acad Periodontol. 2000 Oct;2(4):101-9. Review. PubMed PMID: 12666967.
Reason: Not related to the clinical question

Kasaj A, Gortan-Kasaj A, Briseno-Marroquin B, Willershausen B. Treatment of severe localized periodontal destruction associated with a cemental tear: a case report and review of the literature. Gen Dent. 2009 Jan-Feb;57(1):e5-9. Review. PubMed PMID: 21466996.
Reason: Not related to the clinical question

Kasaj A, Willershausen B, Reichert C, Röhrig B, Smeets R, Schmidt M. Ability of nanocrystalline hydroxyapatite paste to promote human periodontal ligament cell proliferation. J Oral Sci. 2008 Sep;50(3):279-85. PubMed PMID: 18818463.
Reason: Not related to the clinical question

Kauvar AS, Thoma DS, Carnes DL, Cochran DL. In vivo angiogenic activity of enamel matrix derivative. J Periodontol. 2010 Aug;81(8):1196-201. doi: 10.1902/jop.2010.090441. PubMed PMID: 20370422.
Reason: Not related to the clinical question

Keila S, Nemcovsky CE, Moses O, Artzi Z, Weinreb M. In vitro effects of enamel matrix proteins on rat bone marrow cells and gingival fibroblasts. J Dent Res. 2004 Feb;83(2):134-8. PubMed PMID: 14742651.
Reason: Animal study

Kémoun P, Gronthos S, Snead ML, Rue J, Courtois B, Vaysse F, Salles JP, Brunel G. The role of cell surface markers and enamel matrix derivatives on human periodontal ligament mesenchymal progenitor responses in vitro. Biomaterials. 2011 Oct;32(30):7375-88. doi: 10.1016/j.biomaterials.2011.06.043. Epub 2011 Jul 23. PubMed PMID: 21784516; PubMed Central PMCID: PMC4441221.
Reason: Not related to the clinical question

Kerezoudis NP, Siskos GJ, Tsatsas V. Bilateral buccal radicular groove in maxillary incisors: case report. Int Endod J. 2003 Dec;36(12):898-906. PubMed PMID: 14689959.
Reason: Not related to the clinical question

Kim TH, Bae CH, Lee JC, Ko SO, Yang X, Jiang R, Cho ES. β-catenin is required in odontoblasts for tooth root formation. J Dent Res. 2013 Mar;92(3):215-21. doi: 10.1177/0022034512470137. Epub 2013 Jan 23. PubMed PMID: 23345535.
Reason: Not related to the clinical question

Kinaia BM, Chogle SM, Kinaia AM, Goodis HE. Regenerative therapy: a periodontal-endodontic perspective. Dent Clin North Am. 2012 Jul;56(3):537-47. doi: 10.1016/j.cden.2012.05.002. Epub 2012 Jun 23. Review. PubMed PMID: 22835536.
Reason: Not related to the clinical question

King GN. New regenerative technologies: rationale and potential for periodontal regeneration: 2. Growth factors. Dent Update. 2001 Mar;28(2):60-5. Review. PubMed PMID: 11819959.
Maurer S, Leone CW. Use of a serially layered, double connective tissue graft approach to enhance maxillary anterior esthetics. Int J Periodontics Restorative Dent. 2001 Oct;21(5):497-503. PubMed PMID: 11693242.
Reason: Not related to the clinical question

Kinoshita A, Oda S, Takahashi K, Yokota S, Ishikawa I. Periodontal regeneration by application of recombinant human bone morphogenetic protein-2 to horizontal circumferential defects created by experimental periodontitis in beagle dogs. J Periodontol. 1997 Feb;68(2):103-9. PubMed PMID: 9058326.
Reason: Not related to the clinical question

Kinumatsu T, Umehara K, Nagano K, Saito A. Periodontal therapy for severe chronic periodontitis with periodontal regeneration and different types of prosthesis. Bull Tokyo Dent Coll. 2014;55(4):217-24. PubMed PMID: 25477039.
Reason: Not related to the clinical question

Kirtley GE. The Synesthetic Effect. Dent Today. 2015 Mar;34(3):104, 106, 108-9. PubMed PMID: 26349273.
Reason: Not related to the clinical question

Kitamura M, Akamatsu M, Kawanami M, Furuichi Y, Fujii T, Mori M, Kunimatsu K, Shimauchi H, Ogata Y, Yamamoto M, Nakagawa T, Sato S, Ito K, Ogasawara T, Izumi Y, Gomi K, Yamazaki K, Yoshie H, Fukuda M, Noguchi T, Takashiba S, Kurihara H, Nagata T, Hamachi T, Maeda K, Yokota M, Sakagami R, Hara Y, Noguchi K, Furuuchi T, Sasano T, Imai E, Ohmae M, Koizumi H, Watanuki M, Murakami S. Randomized Placebo-Controlled and Controlled Non-Inferiority Phase III Trials Comparing Trafermin, a Recombinant Human Fibroblast Growth Factor 2, and Enamel Matrix Derivative in Periodontal Regeneration in Intrabony Defects. J Bone Miner Res. 2016 Apr;31(4):806-14. doi: 10.1002/jbmr.2738. Epub 2015 Dec 6. PubMed PMID: 26547659.
Reason: Not related to the clinical question

Koop R, Merheb J, Quirynen M. Periodontal regeneration with enamel matrix derivative in reconstructive periodontal therapy: a systematic review. J Periodontol. 2012 Jun;83(6):707-20. doi: 10.1902/jop.2011.110266. Epub 2011 Nov 3. Review. PubMed PMID: 22050544.
Reason: Not related to the clinical question

Kotschy P, Laky M. Reconstruction of supracrestal alveolar bone lost as a result of severe chronic periodontitis. Five-year outcome: case report. Int J Periodontics Restorative Dent. 2006 Oct;26(5):425-31. PubMed PMID: 17073352.
Reason: Not related to the clinical question

Komiya-Ito A, Tomita S, Kinumatsu T, Fujimoto Y, Tsunoda M, Saito A. Longitudinal supportive periodontal therapy for severe chronic periodontitis with furcation involvement: a 12-year follow-up report. Bull Tokyo Dent Coll. 2013;54(4):243-50. PubMed PMID: 24521550.
Reason: Not related to the clinical question

Kramers-de Quervain IA, Läuffer JM, Käch K, Trentz O, Stüssi E. Functional donor-site morbidity during level and uphill gait after a gastrocnemius or soleus muscle-flap procedure. J Bone Joint Surg Am. 2001 Feb;83-A(2):239-46. PubMed PMID: 11216686.
Reason: Not related to the clinical question

Kunimatsu R, Yoshimi Y, Hirose N, Awada T, Miyauchi M, Takata T, Li W, Zhu L, Denbesten PK, Tanimoto K. The C-terminus of amelogenin enhances osteogenic differentiation of human cementoblast lineage cells. J Periodontal Res. 2017 Apr;52(2):218-224. doi: 10.1111/jre.12384. Epub 2016 May 5. PubMed PMID: 27146486.
Reason: Not related to the clinical question

Kuo TF, Lin HC, Yang KC, Lin FH, Chen MH, Wu CC, Chang HH. Bone marrow combined with dental bud cells promotes tooth regeneration in miniature pig model. Artif Organs. 2011 Feb;35(2):113-21. doi: 10.1111/j.1525-1594.2010.01064.x. Epub 2010 Nov 18. PubMed PMID: 21083830.
Reason: Animal study

Kuru BE. Treatment of localized gingival recessions using enamel matrix derivative as an adjunct to laterally sliding flap: 2 case reports. Quintessence Int. 2009 Jun;40(6):461-9. PubMed PMID: 19587887.
Laurentjoye M, Ricard AS, Caix P, Siberchicot F, Majoufre-Lefebvre C. [Tongue reconstruction with a bilateral infrahyoid flap innervated by Ansa Cervicalis after total glossectomy]. Rev Stomatol Chir Maxillofac. 2011 Dec;112(6):337-41. doi: 10.1016/j.stomax.2011.08.015. Epub 2011 Sep 22. French. PubMed PMID: 21943495.
Reason: Not related to the clinical question

Kuru B, Yilmaz S, Argin K, Noyan U. Enamel matrix derivative alone or in combination with a bioactive glass in wide intrabony defects. Clin Oral Investig. 2006 Sep;10(3):227-34. Epub 2006 May 16. PubMed PMID: 16703336.
Reason: Biomaterial different from autologous bone

Lam K, Sae-Lim V. The effect of Emdogain gel on periodontal healing in replanted monkeys' teeth. Oral Surg Oral Med Oral Pathol Oral Radiol Endod. 2004 Jan;97(1):100-7. PubMed PMID: 14716264.
Reason: Animal study

Lee CH, Hajibandeh J, Suzuki T, Fan A, Shang P, Mao JJ. Three-dimensional printed multiphase scaffolds for regeneration of periodontium complex. Tissue Eng Part A. 2014 Apr;20(7-8):1342-51. doi: 10.1089/ten.TEA.2013.0386. Epub 2014 Feb 6. PubMed PMID: 24295512; PubMed Central PMCID: PMC3993023.
Reason: Not related to the clinical question

Leknes KN, Andersen KM, Bøe OE, Skavland RJ, Albandar JM. Enamel matrix derivative versus bioactive ceramic filler in the treatment of intrabony defects: 12-month results. J Periodontol. 2009 Feb;80(2):219-27. doi: 10.1902/jop.2009.080236 . PubMed PMID: 19186961.
Reason: Autologous bone wasn't used

Lekovic V, Camargo PM, Weinlaender M, Kenney EB, Vasilic N. Combination use of bovine porous bone mineral, enamel matrix proteins, and a bioabsorbable membrane in intrabony periodontal defects in humans. J Periodontol. 2001 May;72(5):583-9. PubMed PMID: 11394392.
Reason: Biomaterial different from autologous bone

Lekovic V, Camargo PM, Weinlaender M, Nedic M, Aleksic Z, Kenney EB. A comparison between enamel matrix proteins used alone or in combination with bovine porous bone mineral in the treatment of intrabony periodontal defects in humans. J Periodontol. 2000 Jul;71(7):1110-6. PubMed PMID: 10960017.
Reason: Biomaterial different from autologous bone

Lekovic V, Camargo PM, Weinlaender M, Vasilic N, Djordjevic M, Kenney EB. The use of bovine porous bone mineral in combination with enamel matrix proteins or with an autologous fibrinogen/fibronectin system in the treatment of intrabony periodontal defects in humans. J Periodontol. 2001 Sep;72(9):1157-63. PubMed PMID: 11577946.
Reason: Biomaterial different from autologous bone

Leong CC, Zhou XD, Li JY, Li W, Zhang LL. Possibilities and potential roles of the functional peptides based on enamel matrix proteins in promoting the remineralization of initial enamel caries. Med Hypotheses. 2011 Mar;76(3):391-4. doi: 10.1016/j.mehy.2010.10.050. Epub 2010 Nov 23. PubMed PMID: 21106304.
Reason: Not related to the clinical question

Leung G, Jin L. A combined approach of enamel matrix derivative gel and autogenous bone grafts in treatment of intrabony periodontal defects. A case report. Prim Dent Care. 2003 Apr;10(2):41-3. PubMed PMID: 12736957.
Reason: Not a RCT

Levin I, Ashkenazi M, Schwartz-Arad D. [Preservation of alveolar bone of un-restorable traumatized maxillary incisors for future]. Refuat Hapeh Vehashinayim (1993). 2004 Jan;21(1):54-9, 101-2. Hebrew. PubMed PMID: 15065385.
Reason: Not related to the clinical question

Listl S, Tu YK, Faggion CM Jr. A cost-effectiveness evaluation of enamel matrix derivatives alone or in conjunction with regenerative devices in the treatment of periodontal intra-osseous defects. J Clin Periodontol. 2010 Oct;37(10):920-7. doi: 10.1111/j.1600-051X.2010.01611.x. PubMed PMID: 20727057
Reason: Autologous bone wasn't used

Li XY, Lin-li, Pan YP. [Enamel matrix proteins in the treatment of intrabony defects: A cochrane systematic review]. Shanghai Kou Qiang Yi Xue. 2009 Oct;18(5):454-60. Review. Chinese. PubMed PMID: 19907847.
Reason: Autologous bone wasn't used

Li W, Xiao L, Hu J. The use of enamel matrix derivative alone versus in combination with bone grafts to treat patients with periodontal intrabony defects: a meta-analysis. J Am Dent Assoc. 2012 Sep;143(9):e46-56. Review. PubMed PMID: 22942155.
Reason: Autologous bone wasn't used

Liening DA, Haupert M, Kartush J. Cochlear implantation and variation of facial nerve anatomy: a case report. Ear Nose Throat J. 1994 Sep;73(9):667-9. PubMed PMID: 7988396.
Reason: Not related to the clinical question

Lim HC, Lee JS, Jung UW, Choi SH. Bone Regenerative Potential of Enamel Matrix Protein in the Circumferential Defect Around a Dental Implant. Implant Dent. 2016 Apr;25(2):179-85. doi: 10.1097/ID.0000000000000383. PubMed PMID: 26836128.

Reason: Autologous bone effects weren't evaluated

Lin ZK, Shu R, Song ZC, Cheng L, Dong JC. [The effect of rhAm and EMPs on promoting differentiation of hBMSCs into osteoblasts]. Shanghai Kou Qiang Yi Xue. 2015 Aug;24(4):390-4. Chinese. PubMed PMID: 26383559.
Reason: Not related to the clinical question

Lingaraj K, Lim AY, Puhaindran ME, Kumar PV. Case report: the split flexor carpi ulnaris as a local muscle flap. Clin Orthop Relat Res. 2007 Feb;455:262-6. PubMed PMID: 16906121.
Reason: Not related to the clinical question

Liu F, Zhou ZF, An Y, Yu Y, Wu RX, Yin Y, Xue Y, Chen FM. Effects of cathepsin K on Emdogain-induced hard tissue formation by human periodontal ligament stem cells. J Tissue Eng Regen Med. 2017 Oct;11(10):2922-2934. doi: 10.1002/term.2195. Epub 2016 Jul 12. PubMed PMID: 27401615.
Reason: Not related to the clinical question

Liu Y, Duan D, Xin Y, Bai L, Li T, Li C, Xu Y. A review of the literature: antibiotic usage and its relevance to the infection in periodontal flaps. Acta Odontol Scand. 2017 May;75(4):288-293. doi: 10.1080/00016357.2017.1295165. Epub 2017 Mar 10. Review. PubMed PMID: 28281367.
Reason: Not related to the clinical question

Liu Y, Hu B, Zhou J, Li W, Liu Q, Song J. The Effect of Enamel Matrix Derivative Alone Versus in Combination with Alloplastic Materials to Treat Intrabony Defects: A Meta-analysis. Int J Periodontics Restorative Dent. 2017 Jul/Aug;37(4):e224-e233. doi: 10.11607/prd.2900. PubMed PMID: 28609503.
Reason: Biomaterials different from autologous bone

Losada M, González R, Garcia ÀP, Santos A, Nart J. Treatment of Non-Contained Infrabony Defects With Enamel Matrix Derivative Alone or in Combination With Biphasic Calcium Phosphate Bone Graft: A 12-Month Randomized Controlled Clinical Trial. J Periodontol. 2017 May;88(5):426-435. doi: 10.1902/jop.2016.160459. Epub 2016 Dec 13. PubMed PMID: 27958765.
Reason: Biomaterials different from autologous bone

Lukács L, Gera I. [The management of a single Miller-I type gingival recession at the maxillar incisor with single tunnel technique combined with enamel matrix derivative and connective tissue graft. A case report]. Fogorv Sz. 2011 Mar;104(1):19-26. Hungarian. PubMed PMID: 21789932.
Reason: Not related to the clinical question

Lyngstadaas SP, Lundberg E, Ekdahl H, Andersson C, Gestrelius S. Autocrine growth factors in human periodontal ligament cells cultured on enamel matrix derivative. J Clin Periodontol. 2001 Feb;28(2):181-8. PubMed PMID: 11168744.
Reason: Autologous bone effects weren't evaluated

Lyngstadaas SP, Wohlfahrt JC, Brookes SJ, Paine ML, Snead ML, Reseland JE. Enamel matrix proteins; old molecules for new applications. Orthod Craniofac Res. 2009 Aug;12(3):243-53. doi: 10.1111/j.1601-6343.2009.01459.x. Review. PubMed PMID: 19627527; PubMed Central PMCID: PMC2825346.
Reason: Autologous bone effects weren't evaluated

Maeda S, Ono Y, Nakamura K, Kuwahara T. Molar uprighting with extrusion for implant site bone regeneration and improvement of the periodontal environment. Int J Periodontics Restorative Dent. 2008 Aug;28(4):375-81. Erratum in: Int J Periodontics Restorative Dent. 2008 Oct;28(5):451. PubMed PMID: 18717376.
Reason: Not related to the clinical question

Majzoub Z, Bobbo M, Atiyeh F, Cordioli G. Two patterns of histologic healing in an intrabony defect following treatment with enamel matrix derivative: a human case report. Int J Periodontics Restorative Dent. 2005 Jun;25(3):283-94. PubMed PMID: 16001741.
Reason: Autologous bone wasn't used

Manor A. Periodontal regeneration with enamel matrix derivative--case reports. J Int Acad Periodontol. 2000 Apr;2(2):44-8. PubMed PMID: 12666960.
Reason: Autologous bone wasn't used

Mardas N, Kraehenmann M, Dard M. Regenerative wound healing in acute degree III mandibular defects in dogs. Quintessence Int. 2012 May;43(5):e48-59. PubMed PMID: 22536596.

Reason: Animal study

Mariotti A. Efficacy of chemical root surface modifiers in the treatment of periodontal disease. A systematic review. Ann Periodontol. 2003 Dec;8(1):205-26. Review. PubMed PMID: 14971255.
Reason: Not related to the clinical question

Mârţu S, Burlui V, Mocanu C, Forna N. [Periodontal regeneration with enamel derivate proteins (Emdogain)--clinical evaluation]. Rev Med Chir Soc Med Nat Iasi. 2000 Oct-Dec;104(4):147-51. Romanian. PubMed PMID: 12089944.
Reason: Autologous bone wasn't used

Masaeli R, Zandsalimi K, Lotfi Z, Tayebi L. Using Enamel Matrix Derivative to Improve Treatment Efficacy in Periodontal Furcation Defects. J Prosthodont. 2018 Oct;27(8):733-736. doi: 10.1111/jopr.12753. Epub 2018 Jan 11. Review. PubMed PMID: 29322600.
Reason: Autologous bone wasn't used

Matarasso M, Iorio-Siciliano V, Blasi A, Ramaglia L, Salvi GE, Sculean A. Enamel matrix derivative and bone grafts for periodontal regeneration of intrabony defects. A systematic review and meta-analysis. Clin Oral Investig. 2015 Sep;19(7):1581-93. doi: 10.1007/s00784-015-1491-7. Epub 2015 May 27. Review. PubMed PMID: 26008887.
Reason: Biomaterials different from autologous bone

Matias MA, Li H, Young WG, Bartold PM. Immunohistochemical localisation of extracellular matrix proteins in the periodontium during cementogenesis in the rat molar. Arch Oral Biol. 2003 Oct;48(10):709-16. PubMed PMID: 12971948.
Reason: Animal study

Matsumoto N, Minakami M, Hatakeyama J, Haruna C, Morotomi T, Izumi T, Anan H. Histologic evaluation of the effects of Emdogain gel on injured root apex in rats. J Endod. 2014 Dec;40(12):1989-94. doi: 10.1016/j.joen.2014.08.024. Epub 2014 Oct 7. PubMed PMID: 25305237.
Reason: Animal study

Matsuura M, Herr Y, Han KY, Lin WL, Genco RJ, Cho MI. Immunohistochemical expression of extracellular matrix components of normal and healing periodontal tissues in the beagle dog. J Periodontol. 1995 Jul;66(7):579-93. Erratum in: J Periodontol. 1995 Oct;66(10):905-914. J Periodontol 1995 Oct;66(10):905-14. PubMed PMID: 7562350.
Reason: Animal study

Mavropoulos A, Brodin P, Rösing CK, Aass AM, Aars H. Gingival blood flow in periodontitis patients before and after periodontal surgery assessed in smokers and non-smokers. J Periodontol. 2007 Sep;78(9):1774-82. PubMed PMID: 17760548.
Reason: Not related to the clinical question

McClain PK. Advances in regeneration: restoratively driven, periodontally enhanced. Compend Contin Educ Dent. 2011 Nov-Dec;32 Spec No 5:9-15. PubMed PMID: 22439256.
Reason: Not related to the clinical question

McGuire MK, Cochran DL. Evaluation of human recession defects treated with coronally advanced flaps and either enamel matrix derivative or connective tissue. Part 2: Histological evaluation. J Periodontol. 2003 Aug;74(8):1126-35. PubMed PMID: 14514225.
Reason: Not related to the clinical question

McGuire MK, Nunn M. Evaluation of human recession defects treated with coronally advanced flaps and either enamel matrix derivative or connective tissue. Part 1: Comparison of clinical parameters. J Periodontol. 2003 Aug;74(8):1110-25. PubMed PMID: 14514224.
Reason: Autologous bone wasn't used

McGuire MK, Scheyer ET, Schupbach P. A Prospective, Case-Controlled Study Evaluating the Use of Enamel Matrix Derivative on Human Buccal Recession Defects: A Human Histologic Examination. J Periodontol. 2016 Jun;87(6):645-53. doi: 10.1902/jop.2016.150459. Epub 2016 Feb 1. PubMed PMID: 26832834.
Reason: Not related to the clinical question

Mellonig JT. Enamel matrix derivative for periodontal reconstructive surgery: technique and clinical and histologic case report. Int J Periodontics Restorative Dent. 1999 Feb;19(1):8-19. PubMed PMID: 10379282.
Reason: Autologous bone wasn't used

Mellonig JT, Valderrama P, Gregory HJ, Cochran DL. Clinical and histologic evaluation of non-surgical periodontal therapy with enamel matrix derivative: a report of four cases. J Periodontol. 2009 Sep;80(9):1534-40. doi: 10.1902/jop.2009.090160. PubMed PMID: 19722806.
Reason: Autologous bone wasn't used

Mercado F, Hamlet S, Ivanovski S. Regenerative surgical therapy for peri-implantitis using deproteinized bovine bone mineral with 10% collagen, enamel matrix derivative and Doxycycline-A prospective 3-year cohort study. Clin Oral Implants Res. 2018 Jun;29(6):583-591. doi: 10.1111/clr.13256. Epub 2018 May 16. PubMed PMID: 29767434.
Reason: Biomaterial different from autologous bone

Messenger MP, Raif el M, Seedhom BB, Brookes SJ. The potential use of enamel matrix derivative for in situ anterior cruciate ligament tissue engineering: a translational in vitro investigation. Tissue Eng. 2007 Aug;13(8):2041-51. PubMed PMID: 17518724.
Reason: Not related to the clinical question

Meyle J, Gonzales JR, Bödeker RH, Hoffmann T, Richter S, Heinz B, Arjomand M, Reich E, Sculean A, Jepsen K, Jepsen S. A randomized clinical trial comparing enamel matrix derivative and membrane treatment of buccal class II furcation involvement in mandibular molars. Part II: secondary outcomes. J Periodontol. 2004 Sep;75(9):1188-95. PubMed PMID: 15515332.
Reason: Autologous bone wasn't used

Meyle J, Hoffmann T, Topoll H, Heinz B, Al-Machot E, Jervøe-Storm PM, Meiss C, Eickholz P, Jepsen S. A multi-centre randomized controlled clinical trial on the treatment of intra-bony defects with enamel matrix derivatives/synthetic bone graft or enamel matrix derivatives alone: results after 12 months. J Clin Periodontol. 2011 Jul;38(7):652-60. doi: 10.1111/j.1600-051X.2011.01726.x. Epub 2011 May 12. PubMed PMID: 21564156.
Reason: Biomaterial different from autologous bone

Miliauskaite A, Selimovic D, Hannig M. Successful management of aggressive periodontitis by regenerative therapy: a 3-year follow-up case report. J Periodontol. 2007 Oct;78(10):2043-50. PubMed PMID: 18062127.
Reason: Autologous bone wasn't used

Miliauskaite A, Selimovic D, Hassan M, Nagano F, Soell M, Sano H, Puriene A. Papilla preservation technique combined with Emdogain in the treatment of intrabony defects: a novel treatment regimen for chronic periodontitis. Stomatologija. 2008;10(1):22-6. PubMed PMID: 18493162.
Reason: Autologous bone wasn't used

Minabe M, Kodama T, Kogou T, Takeuchi K, Fushimi H, Sugiyama T, Mitarai E. A comparative study of combined treatment with a collagen membrane and enamel matrix proteins for the regeneration of intraosseous defects. Int J Periodontics Restorative Dent. 2002 Dec;22(6):595-605. PubMed PMID: 12516831.
Reason: Autologous bone wasn't used

Minsk L. The role of enamel matrix proteins in periodontal regeneration. Compend Contin Educ Dent. 2000 Mar;21(3):210-2, 214. PubMed PMID: 11199700.
Reason: Autologous bone wasn't used

Miron RJ, Bosshardt DD, Buser D, Zhang Y, Tugulu S, Gemperli A, Dard M, Caluseru OM, Chandad F, Sculean A. Comparison of the capacity of enamel matrix derivative gel and enamel matrix derivative in liquid formulation to adsorb to bone grafting materials. J Periodontol. 2015 Apr;86(4):578-87. doi: 10.1902/jop.2015.140538. Epub 2015 Jan 16. PubMed PMID: 25594536.
Reason: Autologous bone wasn't used

Miron RJ, Bosshardt DD, Zhang Y, Buser D, Sculean A. Gene array of primary human osteoblasts exposed to enamel matrix derivative in combination with a natural bone mineral. Clin Oral Investig. 2013 Mar;17(2):405-10. doi: 10.1007/s00784-012-0742-0. Epub 2012 May 3. PubMed PMID: 22552595.
Reason: Biomaterial different from autologous bone

Miron RJ, Wei L, Bosshardt DD, Buser D, Sculean A, Zhang Y. Effects of enamel matrix proteins in combination with a bovine-derived natural bone mineral for the repair of bone defects. Clin Oral Investig. 2014;18(2):471-8. doi: 10.1007/s00784-013-0992-5. Epub 2013 May 8. PubMed PMID: 23652357.
Reason: Biomaterial different from autologous bone

Miron RJ, Bosshardt DD, Gemperli AC, Dard M, Buser D, Gruber R, Sculean A. In vitro characterization of a synthetic calcium phosphate bone graft on periodontal ligament cell and osteoblast behavior and its combination with an enamel matrix derivative. Clin Oral Investig. 2014;18(2):443-51. doi: 10.1007/s00784-013-0977-4. Epub 2013 Apr 26. PubMed PMID: 23620149.
Reason: Biomaterial different from autologous bone

Miron RJ, Bosshardt DD, Hedbom E, Zhang Y, Haenni B, Buser D, Sculean A. Adsorption of enamel matrix proteins to a bovine-derived bone grafting material and its regulation of cell adhesion, proliferation, and differentiation. J Periodontol. 2012 Jul;83(7):936-47. doi: 10.1902/jop.2011.110480. Epub 2011 Dec 5. PubMed PMID: 22141360.
Reason: Biomaterial different from autologous bone

Miron RJ, Bosshardt DD, Laugisch O, Dard M, Gemperli AC, Buser D, Gruber R, Sculean A. In vitro evaluation of demineralized freeze-dried bone allograft in combination with enamel matrix derivative. J Periodontol. 2013 Nov;84(11):1646-54. doi: 10.1902/jop.2013.120574. Epub 2013 Jan 24. PubMed PMID: 23347347.
Reason: Biomaterial different from autologous bone

Miron RJ, Caluseru OM, Guillemette V, Zhang Y, Gemperli AC, Chandad F, Sculean A. Influence of enamel matrix derivative on cells at different maturation stages of differentiation. PLoS One. 2013 Aug 12;8(8):e71008. doi: 10.1371/journal.pone.0071008. eCollection 2013. PubMed PMID: 23951068; PubMed Central PMCID: PMC3741386.
Reason: Autologous bone wasn't used

Miron RJ, Chandad F, Buser D, Sculean A, Cochran DL, Zhang Y. Effect of Enamel Matrix Derivative Liquid on Osteoblast and Periodontal Ligament Cell Proliferation and Differentiation. J Periodontol. 2016 Jan;87(1):91-9. doi: 10.1902/jop.2015.150389. Epub 2015 Sep 3. PubMed PMID: 26334247.
Reason: Autologous bone wasn't used

Miron RJ, Fujioka-Kobayashi M, Buser D, Zhang Y, Bosshardt DD, Sculean A. Combination of Collagen Barrier Membrane with Enamel Matrix Derivative-Liquid Improves Osteoblast Adhesion and Differentiation. Int J Oral Maxillofac Implants. 2017 Jan/Feb;32(1):196-203. doi: 10.11607/jomi.5011. PubMed PMID: 28095524.
Reason: Autologous bone wasn't used

Miron RJ, Fujioka-Kobayashi M, Zhang Y, Caballé-Serrano J, Shirakata Y, Bosshardt DD, Buser D, Sculean A. Osteogain improves osteoblast adhesion, proliferation and differentiation on a bovine-derived natural bone mineral. Clin Oral Implants Res. 2017 Mar;28(3):327-333. doi: 10.1111/clr.12802. Epub 2016 Feb 26. PubMed PMID: 26919609.
Reason: Not related to the clinical question

Miron RJ, Guillemette V, Zhang Y, Chandad F, Sculean A. Enamel matrix derivative in combination with bone grafts: A review of the literature. Quintessence Int. 2014 Jun;45(6):475-87. doi: 10.3290/j.qi.a31541. Review. PubMed PMID: 24618572.
Reason: Biomaterial different from autologous bone

Miron RJ, Hedbom E, Ruggiero S, Bosshardt DD, Zhang Y, Mauth C, Gemperli AC, Iizuka T, Buser D, Sculean A. Premature osteoblast clustering by enamel matrix proteins induces osteoblast differentiation through up-regulation of connexin 43 and N-cadherin. PLoS One. 2011;6(8):e23375. doi: 10.1371/journal.pone.0023375. Epub 2011 Aug 15. PubMed PMID: 21858092; PubMed Central PMCID: PMC3156132.
Reason: Not related to the clinical question

Miron RJ, Oates CJ, Molenberg A, Dard M, Hamilton DW. The effect of enamel matrix proteins on the spreading, proliferation and differentiation of osteoblasts cultured on titanium surfaces. Biomaterials. 2010 Jan;31(3):449-60. doi: 10.1016/j.biomaterials.2009.09.075. Epub 2009 Oct 9. PubMed PMID: 19819013.
Reason: Not related to the clinical question

Miron RJ, Sculean A, Cochran DL, Froum S, Zucchelli G, Nemcovsky C, Donos N, Lyngstadaas SP, Deschner J, Dard M, Stavropoulos A, Zhang Y, Trombelli L, Kasaj A, Shirakata Y, Cortellini P, Tonetti M, Rasperini G, Jepsen S, Bosshardt DD. Twenty years of enamel matrix derivative: the past, the present and the future. J Clin Periodontol. 2016 Aug;43(8):668-83. doi: 10.1111/jcpe.12546. Epub 2016 May 28. Review. PubMed PMID: 26987551.
Reason: Autologous bone wasn't used

Miron RJ, Shuang Y, Sculean A, Buser D, Chandad F, Zhang Y. Gene array of PDL cells exposed to Osteogain in combination with a bone grafting material. Clin Oral Investig. 2016 Nov;20(8):2037-2043. Epub 2016 Jan 8. PubMed PMID: 26744181.
Reason: Not related to the clinical question

Miron RJ, Wei L, Yang S, Caluseru OM, Sculean A, Zhang Y. Effect of enamel matrix derivative on periodontal wound healing and regeneration in an osteoporotic model. J Periodontol. 2014 Nov;85(11):1603-11. doi: 10.1902/jop.2014.130745. Epub 2014 May 26. PubMed PMID: 24857323.
Reason: Not related to the clinical question

Mitani A, Takasu H, Horibe T, Furuta H, Nagasaka T, Aino M, Fukuda M, Fujimura T, Mogi M, Noguchi T. Five-year clinical results for treatment of intrabony defects with EMD, guided tissue regeneration and open-flap debridement: a case series. J Periodontal Res. 2015 Feb;50(1):123-30. doi: 10.1111/jre.12188. Epub 2014 May 12. PubMed PMID: 24815103.
Reason: Autologous bone wasn't used

Molina GO, Brentegani LG. Use of enamel matrix protein derivative before dental reimplantation: a histometric analysis. Implant Dent. 2005 Sep;14(3):267-73. PubMed PMID: 16160573.
Reason: Autologous bone wasn't used

Mombelli A, Brochut P, Plagnat D, Casagni F, Giannopoulou C. Enamel matrix proteins and systemic antibiotics as adjuncts to non-surgical periodontal treatment: clinical effects. J Clin Periodontol. 2005 Mar;32(3):225-30. PubMed PMID: 15766363.
Reason: Autologous bone wasn't used

Moreno Rodríguez JA, Ortiz Ruiz AJ, Caffesse RG. Periodontal reconstructive surgery of deep intraosseous defects using an apical approach. Non-incised papillae surgical approach (NIPSA): A retrospective cohort study. J Periodontol. 2018 Nov 12. doi: 10.1002/JPER.18-0405. [Epub ahead of print] PubMed PMID:30421495.
Reason: Not related to the clinical question

Moriyama T, Matsumoto S, Makiishi T. Root coverage technique with enamel matrix derivative. Bull Tokyo Dent Coll. 2009 May;50(2):97-104. PubMed PMID: 19815997.
Reason: Autologous bone wasn't used

Mounir MM, Matar MA, Lei Y, Snead ML. Recombinant Amelogenin Protein Induces Apical Closure and Pulp Regeneration in Open-apex, Nonvital Permanent Canine Teeth. J Endod. 2016 Mar;42(3):402-12. doi: 10.1016/j.joen.2015.11.003. Epub 2015 Dec 18. PubMed PMID: 26709200; PubMed Central PMCID: PMC4766029.
Reason: Not related to the clinical question

Mrozik KM, Gronthos S, Menicanin D, Marino V, Bartold PM. Effect of coating Straumann Bone Ceramic with Emdogain on mesenchymal stromal cell hard tissue formation. Clin Oral Investig. 2012 Jun;16(3):867-78. doi: 10.1007/s00784-011-0558-3. Epub 2011 May 17. PubMed PMID: 21584694.
Reason: Not related to the clinical question

Mueller VT, Welch K, Bratu DC, Wang HL. Early and late studies of EMD use in periodontal intrabony defects. J Periodontal Res. 2013 Feb;48(1):117-25. doi: 10.1111/j.1600-0765.2012.01510.x. Epub 2012 Aug 2. PubMed PMID: 22860751.
Reason: Autologous bone wasn't used

Munhoz EA, Ferreira Junior O, Yaedu RY, Granjeiro JM. Radiographic assessment of impacted mandibular third molar sockets filled with composite xenogenic bone graft. Dentomaxillofac Radiol. 2006 Sep;35(5):371-5. PubMed PMID: 16940486.
Reason: Not related to the clinical question

Mutaf M, Bulut O, Sunay M, Can A. Bilateral musculocutaneous unequal-Z procedure: a new technique for reconstruction of total lower-lip defects. Ann Plast Surg. 2008 Feb;60(2):162-8. doi: 10.1097/SAP.0b013e3180582533. PubMed PMID: 18216509.
Reason: Not related to the clinical question

Nakajima K, Yamaguchi T, Maki K. Surgical orthodontic treatment for a patient with advanced periodontal disease: evaluation with electromyography and 3-dimensional cone-beam computed tomography. Am J Orthod Dentofacial Orthop. 2009 Sep;136(3):450-9. doi: 10.1016/j.ajodo.2007.03.042. PubMed PMID: 19732680.

Reason: Not related to the clinical question

Nakayama Y, Takei-Obi M, Toyoshima-Matsumura I, Tsutamori M, Kato A, Okano C, Mezawa M, Ogata Y. Clinical usability of aspartate aminotransferase to evaluate the prognosis of periodontal regeneration therapies: prospective, longitudinal study. Odontology. 2018 Jul;106(3):306-315. doi: 10.1007/s10266-017-0328-z. Epub 2017 Dec 18. PubMed PMID: 29256042.
Reason: Not related to the clinical question

Nakayama Y, Yang L, Mezawa M, Araki S, Li Z, Wang Z, Sasaki Y, Takai H, Nakao S, Fukae M, Ogata Y. Effects of porcine 25 kDa amelogenin and its proteolytic derivatives on bone sialoprotein expression. J Periodontal Res. 2010 Oct;45(5):602-11. doi: 10.1111/j.1600-0765.2010.01272.x. Epub 2010 Jun 10. PubMed PMID: 20546115.
Reason: Not related to the clinical question

Nath SG, Raveendran R. An insight into the possibilities of fibroblast growth factor in periodontal regeneration. J Indian Soc Periodontol. 2014 May;18(3):289-92. doi: 10.4103/0972-124X.134560. Review. PubMed PMID: 25024539; PubMed Central PMCID: PMC4095618.
Reason: Not related to the clinical question

Neeley WW, Carnes DL, Cochran DL. Osteogenesis in an in vitro coculture of human periodontal ligament fibroblasts and human microvascular endothelial cells. J Periodontol. 2010 Jan;81(1):139-49. doi: 10.1902/jop.2009.090027. PubMed PMID: 20059426.
Reason: Not related to the clinical question

Nemcovsky CE, Beitlitum I. Combination Therapy for Reconstructive Periodontal Treatment in the Lower Anterior Area: Clinical Evaluation of a Case Series. Dent J (Basel). 2018 Oct 1;6(4). pii: E50. doi: 10.3390/dj6040050. PubMed PMID: 30275349; PubMed Central PMCID: PMC6313804.
Reason: Not related to the clinical question

Nemcovsky CE, Zahavi S, Moses O, Kebudi E, Artzi Z, Beny L, Weinreb M. Effect of enamel matrix protein derivative on healing of surgical supra-infrabony periodontal defects in the rat molar: a histomorphometric study. J Periodontol. 2006 Jun;77(6):996-1002. PubMed PMID: 16734574.
Reason: Animal study

Nemoto Y, Kubota T, Nohno K, Nezu A, Morozumi T, Yoshie H. Clinical and CBCT Evaluation of Combined Periodontal Regenerative Therapies Using Enamel Matrix Derivative and Deproteinized Bovine Bone Mineral With or Without Collagen Membrane. Int J Periodontics Restorative Dent. 2018 May/Jun;38(3):373-381. doi: 10.11607/prd.3288. PubMed PMID: 29641626.
Reason: Not related to the clinical question

Neves JS, Salmon CR, Omar NF, Narvaes EA, Gomes JR, Novaes PD. Immunolocalization of CSF-1, RANKL and OPG in the enamel-related periodontium of the rat incisor and their implications for alveolar bone remodeling. Arch Oral Biol. 2009 Jul;54(7):651-7. doi: 10.1016/j.archoralbio.2009.04.001. Epub 2009 May 5. PubMed PMID: 19419711.
Reason: Animal study

Nevins ML. Aesthetic and regenerative oral plastic surgery: clinical applications in tissue engineering. Dent Today. 2006 Oct;25(10):142, 144-6; quiz 146-7. PubMed PMID: 17058405.
Reason: Not related to the clinical question

Nevins ML, Camelo M, Schupbach P, Nevins M, Kim SW, Kim DM. Human buccal plate extraction socket regeneration with recombinant human platelet-derived growth factor BB or enamel matrix derivative. Int J Periodontics Restorative Dent. 2011 Sep-Oct;31(5):481-92. PubMed PMID: 21845243.
Reason: Autologous bone wasn't used

Nguyen TT, Mui B, Mehrabzadeh M, Chea Y, Chaudhry Z, Chaudhry K, Tran SD. Regeneration of tissues of the oral complex: current clinical trends and research advances. J Can Dent Assoc. 2013;79:d1. Review. PubMed PMID: 23522126.
Reason: Not related to the clinical question

Nickles K, Dannewitz B, Gallenbach K, Ramich T, Scharf S, Röllke L, Schacher B, Eickholz P. Long-Term Stability After Regenerative Treatment of Infrabony Defects: A Retrospective Case Series. J Periodontol. 2017 Jun;88(6):536-542. doi: 10.1902/jop.2017.160704. Epub 2017 Mar 3. PubMed PMID: 28398116.
Reason: Not related to the clinical question

Nikolopoulos S, Peteinaki E, Castanas E. Immunologic effects of emdogain in humans: one-year results. Int J Periodontics Restorative Dent. 2002 Jun;22(3):269-77. PubMed PMID: 12186349.

Reason: Not related to the clinical question
Ninomiya M, Kamata N, Fujimoto R, Ishimoto T, Suryono, Kido J, Nagayama M, Nagata T. Application of enamel matrix derivative in autotransplantation of an impacted maxillary premolar: a case report. J Periodontol. 2002 Mar;73(3):346-51. PubMed PMID: 11922266.
Reason: Not related to the clinical question

Nokhbehsaim M, Deschner B, Winter J, Bourauel C, Rath B, Jäger A, Jepsen S, Deschner J. Interactions of regenerative, inflammatory and biomechanical signals on bone morphogenetic protein-2 in periodontal ligament cells. J Periodontal Res. 2011 Jun;46(3):374-81. doi: 10.1111/j.1600-0765.2011.01357.x. Epub 2011 Mar 17. PubMed PMID: 21410703.
Reason: Not related to the clinical question

Nozawa T, Sugiyama T, Satoh T, Tanaka K, Enomoto H, Ito K. Connective tissue-bone onlay graft with enamel matrix derivative for treatment of gingival recession: a case report. Int J Periodontics Restorative Dent. 2002 Dec;22(6):559-65. PubMed PMID: 12516827.
Reason: Case report no autologous bone was used

Nuñez J, Sanz-Blasco S, Vignoletti F, Muñoz F, Caffesse RG, Sanz M, Villalobos C, Nuñez L. 17beta-estradiol promotes cementoblast proliferation and cementum formation in experimental periodontitis. J Periodontol. 2010 Jul;81(7):1064-74. doi: 10.1902/jop.2010.090678. PubMed PMID: 20214440.
Reason: Not related to the clinical question

Nuñez J, Sanz M, Hoz-Rodríguez L, Zeichner-David M, Arzate H. Human cementoblasts express enamel-associated molecules in vitro and in vivo. J Periodontal Res. 2010 Dec;45(6):809-14. doi: 10.1111/j.1600-0765.2010.01291.x. PubMed PMID: 20572915.
Reason: Not related to the clinical question

Ogata Y. Bone sialoprotein and its transcriptional regulatory mechanism. J Periodontal Res. 2008 Apr;43(2):127-35. doi: 10.1111/j.1600-0765.2007.01014.x. Review. PubMed PMID: 18302613.
Reason: Not related to the clinical question

Ogihara S, Tarnow DP. Efficacy of forced eruption/enamel matrix derivative with freeze-dried bone allograft or with demineralized freeze-dried bone allograft in infrabony defects: A randomized trial. Quintessence Int. 2015 Jun;46(6):481-90. doi: 10.3290/j.qi.a33936. PubMed PMID: 25918759.
Reason: Not related to the clinical question

Ogihara S, Tarnow DP. Efficacy of enamel matrix derivative with freeze-dried bone allograft or demineralized freeze-dried bone allograft in intrabony defects: a randomized trial. J Periodontol. 2014 Oct;85(10):1351-60. doi: 10.1902/jop.2014.130520. Epub 2014 Mar 24. PubMed PMID: 24660759.
Reason: Not related to the clinical question

Ogihara S, Wang HL. Periodontal regeneration with or without limited orthodontics for the treatment of 2- or 3-wall infrabony defects. J Periodontol. 2010 Dec;81(12):1734-42. doi: 10.1902/jop.2010.100127. Epub 2010 Jul 14. PubMed PMID: 20629545.
Reason: Not related to the clinical question

Okropiridze TV, Menabde GT, Gogilashvili KT, Margvelashvili VV. [Osteoplastic materials in the treatment of parodontosis]. Georgian Med News. 2008 Jul-Aug;(160-161):21-4. Russian. PubMed PMID: 18711232.
Reason: Not related to the clinical question

Okubo K, Kobayashi M, Takiguchi T, Takada T, Ohazama A, Okamatsu Y, Hasegawa K. Participation of endogenous IGF-I and TGF-beta 1 with enamel matrix derivative-stimulated cell growth in human periodontal ligament cells. J Periodontal Res. 2003 Feb;38(1):1-9. PubMed PMID: 12558931.
Reason: Not related to the clinical question

Okuda K, Miyazaki A, Momose M, Murata M, Nomura T, Kubota T, Wolff LF, Yoshie H. Levels of tissue inhibitor of metalloproteinases-1 and matrix metalloproteinases-1 and -8 in gingival crevicular fluid following treatment with enamel matrix derivative (EMDOGAIN). J Periodontal Res. 2001 Oct;36(5):309-16. PubMed PMID: 11585118.
Reason: Not related to the clinical question

Okuda K, Momose M, Miyazaki A, Murata M, Yokoyama S, Yonezawa Y, Wolff LF, Yoshie H. Enamel matrix derivative in the treatment of human intrabony osseous defects. J Periodontol. 2000 Dec;71(12):1821-8. PubMed PMID: 11156038.
Reason: Autologous bone wasn't used

Olivares-Navarrete R, Vesper K, Hyzy SL, Almaguer-Flores A, Boyan BD, Schwartz Z. Role of the N-terminal peptide of amelogenin on osteoblastic differentiation of human mesenchymal stem cells. Eur Cell Mater. 2014 Jul 14;28:1-10; discussion 10. PubMed PMID: 25017640.
Reason: Not related to the clinical question

Onodera H, Shibukawa Y, Sugito H, Ota M, Yamada S. Periodontal regeneration in intrabony defects after application of enamel matrix proteins with guided tissue regeneration: an experimental study in dogs. Biomed Res. 2005 Apr;26(2):69-77. PubMed PMID: 15889620.
Reason: Animal study not related to the clinical question

Oortgiesen DA, Yu N, Bronckers AL, Yang F, Walboomers XF, Jansen JA. A three-dimensional cell culture model to study the mechano-biological behavior in periodontal ligament regeneration. Tissue Eng Part C Methods. 2012 Feb;18(2):81-9. doi: 10.1089/ten.TEC.2011.0367. Epub 2012 Jan 4. PubMed PMID: 21913838; PubMed Central PMCID: PMC3262976.
Reason: Not related to the clinical question

Oortgiesen DA, Meijer GJ, Bronckers AL, Walboomers XF, Jansen JA. Regeneration of the periodontium using enamel matrix derivative in combination with an injectable bone cement. Clin Oral Investig. 2013 Mar;17(2):411-21. doi: 10.1007/s00784-012-0743-z. Epub 2012 May 3. PubMed PMID: 22552596; PubMed Central PMCID: PMC3579465.
Reason: Biomaterial different from autologous bone

Oortgiesen DA, Walboomers XF, Bronckers AL, Meijer GJ, Jansen JA. Periodontal regeneration using an injectable bone cement combined with BMP-2 or FGF-2. J Tissue Eng Regen Med. 2014 Mar;8(3):202-9. doi: 10.1002/term.1514. Epub 2012 May 2. PubMed PMID: 22552898.
Reason: Not related to the clinical question

Otsuka T, Kasai H, Yamaguchi K, Nishihara T. Enamel matrix derivative promotes osteoclast cell formation by RANKL production in mouse marrow cultures. J Dent. 2005 Oct;33(9):749-55. Epub 2005 Mar 31. PubMed PMID: 16199283.
Reason: Not related to the clinical question

Oringer RJ. Biological mediators for periodontal and bone regeneration. Compend Contin Educ Dent. 2002 Jun;23(6):501-4, 506-10, 512 passim; quiz 518. Review. PubMed PMID: 12789966.
Reason: Not related to the clinical question

O'Sullivan KL, Pap SA, Megerian CA, Li Y, Sheffler LR, Smith TW, Lawrence WT. Improved axon diameter and myelin sheath thickness in facial nerve cable grafts wrapped in temporoparietal fascial flaps. Ann Plast Surg. 1998 May;40(5):478-85. PubMed PMID: 9600431.
Reason: Not related to the clinical question

Ozcelik O, Cenk Haytac M, Seydaoglu G. Enamel matrix derivative and low-level laser therapy in the treatment of intra-bony defects: a randomized placebo-controlled clinical trial. J Clin Periodontol. 2008 Feb;35(2):147-56. Epub 2007 Dec 13. PubMed PMID: 18081859.
Reason: Autologous bone wasn't used

Oztürk S, Bayram Y, Möhür H, Deveci M, Sengezer M. Evaluation of late functional results of patients treated with free muscle flaps for heel defects caused by land-mine explosions. Plast Reconstr Surg. 2005 Dec;116(7):1926-36. PubMed PMID: 16327605.
Reason: Not related to the clinical question

Pagliaro U, Nieri M, Rotundo R, Cairo F, Carnevale G, Esposito M, Cortellini P, Pini-Prato G; Italian Society of Periodontology. Clinical guidelines of the Italian Society of Periodontology for the reconstructive surgical treatment of angular bony defects in periodontal patients. J Periodontol. 2008 Dec;79(12):2219-32. doi: 10.1902/jop.2008.080266 . PubMed PMID: 19053910.
Reason: Not related to the clinical question

Palioto DB, de O Macedo G, Queiroz AC, Taba M Jr, Souza SL, Grisi MF, Novaes

AB Jr. Enamel matrix derivative and transforming growth factor-beta1 in Class III furcation defects. A histomorphometric study in dogs. J Int Acad Periodontol. 2012 Jul;14(3):69-75. PubMed PMID: 22908536.
Reason: Not related to the clinical question

Paolantonio M. Combined periodontal regenerative technique in human intrabony defects by collagen membranes and anorganic bovine bone. A controlled clinical study. J Periodontol. 2002 Feb;73(2):158-66. PubMed PMID: 11895280.
Reason: Not related to the clinical question

Parashis A, Andronikaki-Faldami A, Tsiklakis K. Clinical and radiographic comparison of three regenerative procedures in the treatment of intrabony defects. Int J Periodontics Restorative Dent. 2004 Feb;24(1):81-90. PubMed PMID: 14984149.
Reason: No data about the use of EMD and AB in association

Parashis AO, Polychronopoulou A, Tsiklakis K, Tatakis DN. Enamel matrix derivative in intrabony defects: prognostic parameters of clinical and radiographic treatment outcomes. J Periodontol. 2012 Nov;83(11):1346-52. doi: 10.1902/jop.2012.110551. Epub 2012 Jan 16. PubMed PMID: 22248222.
Reason: Autologous bone wasn't used

Parashis A, Tsiklakis K. Clinical and radiographic findings following application of enamel matrix derivative in the treatment of intrabony defects. A series of case reports. J Clin Periodontol. 2000 Sep;27(9):705-13. PubMed PMID: 10983605.
Reason: Autologous bone wasn't used

Parashis AO, Tsiklakis K, Tatakis DN. EDTA gel root conditioning: lack of effect on clinical and radiographic outcomes of intrabony defect treatment with enamel matrix derivative. J Periodontol. 2006 Jan;77(1):103-10. PubMed PMID: 16579710.
Reason: Autologous bone wasn't used

Park JB, Matsuura M, Han KY, Norderyd O, Lin WL, Genco RJ, Cho MI. Periodontal regeneration in class III furcation defects of beagle dogs using guided tissue regenerative therapy with platelet-derived growth factor. J Periodontol. 1995 Jun;66(6):462-77. PubMed PMID: 7562336.
Reason: Animal study not related to the clinical question

Park CH, Oh JH, Jung HM, Choi Y, Rahman SU, Kim S, Kim TI, Shin HI, Lee YS, Yu FH, Baek JH, Ryoo HM, Woo KM. Effects of the incorporation of ε-aminocaproic acid/chitosan particles to fibrin on cementoblast differentiation and cementum regeneration. Acta Biomater. 2017 Oct 1;61:134-143. doi: 10.1016/j.actbio.2017.07.039. Epub 2017 Jul 29. PubMed PMID: 28764948.
Reason: Not related to the clinical question

Parodi R, Liuzzo G, Patrucco P, Brunel G, Santarelli GA, Birardi V, Gasparetto B. Use of Emdogain in the treatment of deep intrabony defects: 12-month clinical results. Histologic and radiographic evaluation. Int J Periodontics Restorative Dent. 2000 Dec;20(6):584-95. PubMed PMID: 11203595.
Reason: Autologous bone wasn't used

Parodi R, Santarelli GA, Gasparetto B. Treatment of intrabony pockets with Emdogain: results at 36 months. Int J Periodontics Restorative Dent. 2004 Feb;24(1):57-63. PubMed PMID: 14984146.
Reason: Autologous bone wasn't used

Parrish LC, Miyamoto T, Fong N, Mattson JS, Cerutis DR. Non-bioabsorbable vs. bioabsorbable membrane: assessment of their clinical efficacy in guided tissue regeneration technique. A systematic review. J Oral Sci. 2009 Sep;51(3):383-400. Review. PubMed PMID: 19776505.
Reason: Not related to the clinical question

Peres MF, Ribeiro ED, Casarin RC, Ruiz KG, Junior FH, Sallum EA, Casati MZ. Hydroxyapatite/β-tricalcium phosphate and enamel matrix derivative for treatment of proximal class II furcation defects: a randomized clinical trial. J Clin Periodontol. 2013 Mar;40(3):252-9. doi: 10.1111/jcpe.12054. PubMed PMID: 23379539.
Reason: Biomaterial different from autologous bone

Petinaki E, Nikolopoulos S, Castanas E. Low stimulation of peripheral lymphocytes, following in vitro application of Emdogain. J Clin Periodontol. 1998

Sep;25(9):715-20. PubMed PMID: 9763326.
Reason: Not related to the clinical question

Pietruska MD. A comparative study on the use of Bio-Oss and enamel matrix derivative (Emdogain) in the treatment of periodontal bone defects. Eur J Oral Sci. 2001 Jun;109(3):178-81. PubMed PMID: 11456348.
Reason: Biomaterial different from autologous bone

Pietruska M, Pietruski J, Nagy K, Brecx M, Arweiler NB, Sculean A. Four-year results following treatment of intrabony periodontal defects with an enamel matrix derivative alone or combined with a biphasic calcium phosphate. Clin Oral Investig. 2012 Aug;16(4):1191-7. doi: 10.1007/s00784-011-0611-2. Epub 2011 Sep 1. PubMed PMID: 21881869.
Reason: Biomaterial different from autologous bone

Pietruska MD, Pietruski JK, Stokowska W. Clinical and radiographic evaluation of periodontal therapy using enamel matrix derivative (Emdogain). Rocz Akad Med Bialymst. 2001;46:198-208. PubMed PMID: 11780564.
Reason: Autologous bone wasn't used

Pimentel SP, Sallum AW, Saldanha JB, Casati MZ, Nociti FH Jr, Sallum EA. Enamel matrix derivative versus guided tissue regeneration in the presence of nicotine: a histomorphometric study in dogs. J Clin Periodontol. 2006 Dec;33(12):900-7. Epub 2006 Sep 13. PubMed PMID: 16970622.
Reason: Autologous bone wasn't used

Plachokova AS, van den Dolder J, Jansen JA. The bone-regenerative properties of Emdogain adsorbed onto poly(D,L-lactic-coglycolic acid)/calcium phosphate composites in an ectopic and an orthotopic rat model. J Periodontal Res. 2008 Feb;43(1):55-63. doi: 10.1111/j.1600-0765.2007.00994.x. PubMed PMID: 18230107.
Reason: Not related to the clinical question

Poi WR, Carvalho RM, Panzarini SR, Sonoda CK, Manfrin TM, Rodrigues Tda S. Influence of enamel matrix derivative (Emdogain) and sodium fluoride on the healing process in delayed tooth replantation: histologic and histometric analysis in rats. Dent Traumatol. 2007 Feb;23(1):35-41. PubMed PMID: 17227379.
Pontoriero R, Wennström J, Lindhe J. The use of barrier membranes and enamel matrix proteins in the treatment of angular bone defects. A prospective controlled clinical study. J Clin Periodontol. 1999 Dec;26(12):833-40. PubMed PMID: 10599912.
Reason: Not related to the clinical question

Popowics T, Foster BL, Swanson EC, Fong H, Somerman MJ. Defining the roots of cementum formation. Cells Tissues Organs. 2005;181(3-4):248-57. PubMed PMID: 16612090.
Reason: Not related to the clinical question

Post M. Pectoralis major transfer for winging of the scapula. J Shoulder Elbow Surg. 1995 Jan-Feb;4(1 Pt 1):1-9. PubMed PMID: 7874558.
Reason: Not related to the clinical question

Prata CA, Lacerda SA, Brentegani LG. Autogenous bone graft associated with enamel matrix proteins in bone repair. Implant Dent. 2007 Dec;16(4):413-20. PubMed PMID: 18091170.
Reason: Animal study

Queiroz LA, Casarin RCV, Dabdoub SM, Tatakis DN, Sallum EA, Kumar PS. Furcation Therapy With Enamel Matrix Derivative: Effects on the Subgingival Microbiome. J Periodontol. 2017 Jul;88(7):617-625. doi: 10.1902/jop.2017.160542. Epub 2017 Mar 17. PubMed PMID: 28304211.
Reason: Autologous bone wasn't used

Queiroz LA, Santamaria MP, Casati MZ, Ruiz KS, Nociti F Jr, Sallum AW, Sallum EA. Enamel matrix protein derivative and/or synthetic bone substitute for the treatment of mandibular class II buccal furcation defects. A 12-month randomized clinical trial. Clin Oral Investig. 2016 Sep;20(7):1597-606. doi: 10.1007/s00784-015-1642-x. Epub 2015 Nov 10. PubMed PMID: 26556577.
Reason: Biomaterial different from autologous bone

Queiroz LA, Santamaria M, Casati M, Silverio K, Nociti-Junior F, Sallum E. Enamel matrix protein derivative plus synthetic bone substitute for the treatment of mandibular Class II furcation defects: a case series. Quintessence Int. 2015 Mar;46(3):199-205. doi: 10.3290/j.qi.a32988. PubMed PMID: 25386635.

Reason: Biomaterial different from autologous bone
Ragghianti Zangrando MS, Chambrone D, Pasin IM, Conde MC, Pannuti CM, de Lima LA. Two-year randomized clinical trial of enamel matrix derivative treated infrabony defects: radiographic analysis. BMC Oral Health. 2014 Dec 4;14:149. doi: 10.1186/1472-6831-14-149. PubMed PMID: 25475143; PubMed Central PMCID: PMC4266965.
Reason: Autologous bone wasn't used

Rasperini G, Acunzo R, Barnett A, Pagni G. The soft tissue wall technique for the regenerative treatment of non-contained infrabony defects: a case series. Int J Periodontics Restorative Dent. 2013 May-Jun;33(3):e79-87. doi: 10.11607/prd.1628. PubMed PMID: 23593632.
Reason: Not related to clinical question

Rasperini G, Ricci G, Silvestri M. Surgical technique for treatment of infrabony defects with enamel matrix derivative (Emdogain): 3 case reports. Int J Periodontics Restorative Dent. 1999 Dec;19(6):578-87. PubMed PMID: 10815596.
Reason: Autologous bone wasn't used

Rasperini G, Silvestri M, Ricci G. Long-term clinical observation of treatment of infrabony defects with enamel matrix derivative (Emdogain): surgical reentry. Int J Periodontics Restorative Dent. 2005 Apr;25(2):121-7. PubMed PMID: 15839588.
Reason: Autologous bone wasn't used

Rathva VJ. Enamel matrix protein derivatives: role in periodontal regeneration. Clin Cosmet Investig Dent. 2011 Dec 1;3:79-92. doi: 10.2147/CCIDEN.S25347. Print 2011. PubMed PMID: 23674918; PubMed Central PMCID: PMC3652362.
Reason: Autologous bone wasn't used

Regazzini PF, Novaes AB Jr, de Oliveira PT, Palioto DB, Taba M Jr, de Souza SL, Grisi MF. Comparative study of enamel matrix derivative with or without GTR in the treatment of class II furcation lesions in dogs. Int J Periodontics Restorative Dent. 2004 Oct;24(5):476-87. PubMed PMID: 15506029.
Reason: Animal study

Rethman MP. Treatment of a palatal-gingival groove using enamel matrix derivative. Compend Contin Educ Dent. 2001 Sep;22(9):792-7. PubMed PMID: 11692401.
Reason: Autologous bone wasn't used

Reynolds MA, Aichelmann-Reidy ME. Protein and peptide-based therapeutics in periodontal regeneration. J Evid Based Dent Pract. 2012 Sep;12(3 Suppl):118-26. doi: 10.1016/S1532-3382(12)70023-4. Review. PubMed PMID: 23040343.
Reason: Not related to clinical question

Reynolds MA, Kao RT, Camargo PM, Caton JG, Clem DS, Fiorellini JP, Geisinger ML, Mills MP, Nares S, Nevins ML. Periodontal regeneration - intrabony defects: a consensus report from the AAP Regeneration Workshop. J Periodontol. 2015 Feb;86(2 Suppl):S105-7. doi: 10.1902/jop.2015.140378. Epub 2014 Oct 15. PubMed PMID: 25315019.
Reason: Not related to clinical question

Ribeiro FV, Casarin RC, Júnior FH, Sallum EA, Casati MZ. The role of enamel matrix derivative protein in minimally invasive surgery in treating intrabony defects in single-rooted teeth: a randomized clinical trial. J Periodontol. 2011 Apr;82(4):522-32. doi: 10.1902/jop.2010.100454. Epub 2010 Nov 8. PubMed PMID: 21054224.
Reason: Autologous bone wasn't used

Ribeiro FV, Nociti Júnior FH, Sallum EA, Sallum AW, Casati MZ. Use of enamel matrix protein derivative with minimally invasive surgical approach in intra-bony periodontal defects: clinical and patient-centered outcomes. Braz Dent J. 2010 Jan;21(1):60-7. PubMed PMID: 20464323.
Reason: Autologous bone wasn't used

Rincon JC, Xiao Y, Young WG, Bartold PM. Production of osteopontin by cultured porcine epithelial cell rests of Malassez. J Periodontal Res. 2005 Oct;40(5):417-26. PubMed PMID: 16105095
Reason: Not related to the clinical question

Rincon JC, Xiao Y, Young WG, Bartold PM. Enhanced proliferation, attachment

and osteopontin expression by porcine periodontal cells exposed to Emdogain. Arch Oral Biol. 2005 Dec;50(12):1047-54. PubMed PMID: 16342405.
Reason: Not related to the clinical question

Rodrigues TL, Marchesan JT, Coletta RD, Novaes AB Jr, Grisi MF, Souza SL, Taba M Jr, Palioto DB. Effects of enamel matrix derivative and transforming growth factor-beta1 on human periodontal ligament fibroblasts. J Clin Periodontol. 2007 Jun;34(6):514-22. PubMed PMID: 17509092.
Reason: Not related to the clinical question

Röllke L, Schacher B, Wohlfeil M, Kim TS, Kaltschmitt J, Krieger J, Krigar DM, Reitmeir P, Eickholz P. Regenerative therapy of infrabony defects with or without systemic doxycycline. A randomized placebo-controlled trial. J Clin Periodontol. 2012 May;39(5):448-56. doi: 10.1111/j.1600-051X.2012.01861.x. Epub 2012 Mar 4. PubMed PMID: 22385260.
Reason: Not related to the clinical question

Rosen PS, Reynolds MA. A retrospective case series comparing the use of demineralized freeze-dried bone allograft and freeze-dried bone allograft combined with enamel matrix derivative for the treatment of advanced osseous lesions. J Periodontol. 2002 Aug;73(8):942-9. PubMed PMID: 12211505.
Reason: Biomaterials different from autologous bone

Rösing CK, Aass AM, Mavropoulos A, Gjermo P. Clinical and radiographic effects of enamel matrix derivative in the treatment of intrabony periodontal defects: a 12-month longitudinal placebo-controlled clinical trial in adult periodontitis patients. J Periodontol. 2005 Jan;76(1):129-33. PubMed PMID: 15830647.
Reason: Autologous bone wasn't used

Rossmann JA, McQuade MJ, Turunen DE. Retardation of epithelial migration in monkeys using a carbon dioxide laser: an animal study. J Periodontol. 1992 Nov;63(11):902-7. PubMed PMID: 1280679.
Reason: Animal study

Sakaguchi K, Katagiri W, Osugi M, Kawai T, Sugimura-Wakayama Y, Hibi H. Periodontal tissue regeneration using the cytokine cocktail mimicking secretomes in the conditioned media from human mesenchymal stem cells. Biochem Biophys Res Commun. 2017 Feb 26;484(1):100-106. doi: 10.1016/j.bbrc.2017.01.065. Epub 2017 Jan 16. PubMed PMID: 28104393.
Reason: Not related to the clinical question

Sakallioğlu U, Açikgöz G, Ayas B, Kirtiloğlu T, Sakallioğlu E. Healing of periodontal defects treated with enamel matrix proteins and root surface conditioning--an experimental study in dogs. Biomaterials. 2004 May;25(10):1831-40. PubMed PMID: 14738847.
Reason: Animal study

Saida H, Fukuba S, Miron R, Shirakata Y. Efficacy of flapless intentional replantation with enamel matrix derivative in the treatment of hopeless teeth associated with endodontic-periodontal lesions: A 2-year prospective case series. Quintessence Int. 2018;49(9):699-707. doi: 10.3290/j.qi.a40782. PubMed PMID: 30027173.
Reason: Not related to the clinical question

Saito A, Hayakawa H, Ota K, Fujinami K, Nikaido M, Makiishi T. Treatment of periodontal defects with enamel matrix derivative: clinical evaluation at early healing stages. Bull Tokyo Dent Coll. 2010;51(2):85-93. PubMed PMID: 20689239.
Reason: Autologous bone wasn't used

Saito K, Konishi I, Nishiguchi M, Hoshino T, Fujiwara T. Amelogenin binds to both heparan sulfate and bone morphogenetic protein 2 and pharmacologically suppresses the effect of noggin. Bone. 2008 Aug;43(2):371-6. doi: 10.1016/j.bone.2008.03.029. Epub 2008 Apr 18. PubMed PMID: 18515207.
Reason: Not related to the clinical question

Saito A, Nanbu Y, Nagahata T, Yamada S. Treatment of intrabony periodontal defects with enamel matrix derivative in private practice: a long-term retrospective study. Bull Tokyo Dent Coll. 2008 May;49(2):89-96. Erratum in: Bull Tokyo Dent Coll. 2008 Aug;49(3):129. PubMed PMID: 18776720.
Saito M, Tsuji T. Extracellular matrix administration as a potential therapeutic strategy for periodontal ligament regeneration. Expert Opin Biol Ther. 2012 Mar;12(3):299-309. doi: 10.1517/14712598.2012.655267. Epub 2012 Jan 26. Review. PubMed PMID: 22276595.
Reason: Not related to the clinical question

Sajjadian A, Song AY, Khorsandi CA, Deleyiannis FW, VanSwearingen JM, Henkelmann TC, Hui K, Manders EK. One-stage reanimation of the paralyzed face using the rectus abdominis neurovascular free flap. Plast Reconstr Surg. 2006 Apr 15;117(5):1553-9. PubMed PMID: 16641724.
Reason: Not related to the clinical question

Sallum EA, Casati MZ, Caffesse RG, Funis LP, Nociti Júnior FH, Sallum AW. Coronally positioned flap with or without enamel matrix protein derivative for the treatment of gingival recessions. Am J Dent. 2003 Oct;16(5):287-91. PubMed PMID: 14677605.
Reason: Autologous bone wasn't used

Sallum EA, Pimentel SP, Saldanha JB, Nogueira-Filho GR, Casati MZ, Nociti FH, Sallum AW. Enamel matrix derivative and guided tissue regeneration in the treatment of dehiscence-type defects: a histomorphometric study in dogs. J Periodontol. 2004 Oct;75(10):1357-63. PubMed PMID: 15562913.
Reason: Animal study

Sallum EA, Ribeiro FV, Ruiz KS, Sallum AW. Experimental and clinical studies on regenerative periodontal therapy. Periodontol 2000. 2019 Feb;79(1):22-55. doi: 10.1111/prd.12246. Review. PubMed PMID: 30892759.
Reason: Not related to the clinical question

Sandhu GK, Khinda PK, Gill AS, Kalra HS. Surgical re-entry evaluation of regenerative efficacy of bioactive Gengigel(®) and platelet-rich fibrin in the treatment of grade II furcation: A novel approach. Contemp Clin Dent. 2015 Oct-Dec;6(4):570-3. doi: 10.4103/0976-237X.169855. PubMed PMID: 26681869; PubMed Central PMCID: PMC4678562.
Reason: Not related to the clinical question

Sanz M, Jepsen K, Eickholz P, Jepsen S. Clinical concepts for regenerative therapy in furcations. Periodontol 2000. 2015 Jun;68(1):308-32. doi: 10.1111/prd.12081. PubMed PMID: 25867991.
Reason: Not related to the clinical question

Sanz M, Tonetti MS, Zabalegui I, Sicilia A, Blanco J, Rebelo H, Rasperini G, Merli M, Cortellini P, Suvan JE. Treatment of intrabony defects with enamel matrix proteins or barrier membranes: results from a multicenter practice-based clinical trial. J Periodontol. 2004 May;75(5):726-33. PubMed PMID: 15212355.
Reason: Not related to the clinical question

Scheyer ET, Velasquez-Plata D, Brunsvold MA, Lasho DJ, Mellonig JT. A clinical comparison of a bovine-derived xenograft used alone and in combination with enamel matrix derivative for the treatment of periodontal osseous defects in humans. J Periodontol. 2002 Apr;73(4):423-32. PubMed PMID: 11990444.
Reason: Biomaterial different from autologous bone

Schjøtt M, Andreasen JO. Emdogain does not prevent progressive root resorption after replantation of avulsed teeth: a clinical study. Dent Traumatol. 2005 Feb;21(1):46-50. PubMed PMID: 15660757.
Reason: Not related to the clinical question

Schmidlin PR, Hauri D, Krähenmann MA, Puhan MA, Attin T. [Residual pocket depth after periodontal regenerative procedures. Clinical relevance and interpretation of meta-analyses data]. Schweiz Monatsschr Zahnmed. 2009;119(3):224-31. Review. German. PubMed PMID: 19408526.
Reason: Not related to the clinical question

Schmidlin PR. Regenerative Treatment of a Cemental Tear Using Enamel Matrix Derivatives: a Ten-Year Follow-up. Open Dent J. 2012;6:148-52. doi: 10.2174/1874210601206010148. Epub 2012 Sep 20. PubMed PMID: 23056160; PubMed Central PMCID: PMC3465863.
Reason: Autologous bone wasn't used

Schröen O, Sahrmann P, Roos M, Attin T, Schmidlin PR. A survey on regenerative surgery performed by Swiss specialists in periodontology with special emphasis on the application of enamel matrix derivatives in infrabony defects. Schweiz Monatsschr Zahnmed. 2011;121(2):136-42. PubMed PMID: 21394687.
Reason: Not related to the clinical question

Schwarz F, Sculean A, Georg T, Becker J. Clinical evaluation of the Er:YAG laser in combination with an enamel matrix protein derivative for the treatment of intrabony periodontal defects: a pilot study. J Clin Periodontol. 2003

Nov;30(11):975-81. PubMed PMID: 14761120.
Reason: Not related to the clinical question

Sculean A, Allen EP. The Laterally Closed Tunnel for the Treatment of Deep Isolated Mandibular Recessions: Surgical Technique and a Report of 24 Cases. Int J Periodontics Restorative Dent. 2018 Jul/Aug;38(4):479-487. doi: 10.11607/prd.3680. PubMed PMID: 29889911.
Reason: Not related to the clinical question

Sculean A, Barbé G, Chiantella GC, Arweiler NB, Berakdar M, Brecx M. Clinical evaluation of an enamel matrix protein derivative combined with a bioactive glass for the treatment of intrabony periodontal defects in humans. J Periodontol. 2002 Apr;73(4):401-8. PubMed PMID: 11990441.
Reason: Biomaterial different from autologous bone

Sculean A, Berakdar M, Donos N, Auschill TM, Arweiler NB. The effect of postsurgical administration of a selective cyclo-oxygenase-2 inhibitor on the healing of intrabony defects following treatment with enamel matrix proteins. Clin Oral Investig. 2003 Jun;7(2):108-12. Epub 2003 Apr 25. PubMed PMID: 12720115.
Reason: Not related to the clinical question

Sculean A, Berakdar M, Windisch P, Remberger K, Donos N, Brecx M. Immunohistochemical investigation on the pattern of vimentin expression in regenerated and intact monkey and human periodontal ligament. Arch Oral Biol. 2003 Jan;48(1):77-86. PubMed PMID: 12615145.
Reason: Not related to the clinical question

Sculean A, Berakdar M, Pahl S, Windisch P, Brecx M, Reich E, Donos N. Patterns of cytokeratin expression in monkey and human periodontium following regenerative and conventional periodontal surgery. J Periodontal Res. 2001 Aug;36(4):260-8. PubMed PMID: 11519700.
Reason: Animal study

Sculean A, Berakdar M, Willershausen B, Arweiler NB, Becker J, Schwarz F. Effect of EDTA root conditioning on the healing of intrabony defects treated with an enamel matrix protein derivative. J Periodontol. 2006 Jul;77(7):1167-72. PubMed PMID: 16805678.
Reason: Not related to the clinical question

Sculean A, Blaes A, Arweiler N, Reich E, Donos N, Brecx M. The effect of postsurgical antibiotics on the healing of intrabony defects following treatment with enamel matrix proteins. J Periodontol. 2001 Feb;72(2):190-5. PubMed PMID: 11288792.
Reason: Not related to the clinical question

Sculean A, Chiantella GC, Arweiler NB, Becker J, Schwarz F, Stavropoulos A. Five-year clinical and histologic results following treatment of human intrabony defects with an enamel matrix derivative combined with a natural bone mineral. Int J Periodontics Restorative Dent. 2008 Apr;28(2):153-61. PubMed PMID: 18546811.
Reason: Biomaterial different from autologous bone

Sculean A, Chiantella GC, Miliauskaite A, Brecx M, Arweiler NB. Four-year results following treatment of intrabony periodontal defects with an enamel matrix protein derivative: a report of 46 cases. Int J Periodontics Restorative Dent. 2003 Aug;23(4):345-51. PubMed PMID: 12956478.
Reason: Autologous bone effect weren't evaluated

Sculean A, Chiantella GC, Windisch P, Donos N. Clinical and histologic evaluation of human intrabony defects treated with an enamel matrix protein derivative (Emdogain). Int J Periodontics Restorative Dent. 2000 Aug;20(4):374-81. PubMed PMID: 11203577.
Reason: Autologous bone wasn't used

Sculean A, Chiantella GC, Windisch P, Gera I, Reich E. Clinical evaluation of an enamel matrix protein derivative (Emdogain) combined with a bovine-derived xenograft (Bio-Oss) for the treatment of intrabony periodontal defects in humans. Int J Periodontics Restorative Dent. 2002 Jun;22(3):259-67. PubMed PMID: 12186348.
Reason: Biomaterial different from autologous bone

Sculean A, Cosgarea R, Stähli A, Katsaros C, Arweiler NB, Miron RJ, Deppe H. Treatment of multiple adjacent maxillary Miller Class I, II, and III gingival recessions with the modified coronally advanced tunnel, enamel matrix derivative,

and subepithelial connective tissue graft: A report of 12 cases. Quintessence Int. 2016;47(8):653-9. doi: 10.3290/j.qi.a36562. PubMed PMID: 27446995.
Reason: Not related to the clinical question

Sculean A, Cosgarea R, Stähli A, Katsaros C, Arweiler NB, Brecx M, Deppe H. The modified coronally advanced tunnel combined with an enamel matrix derivative and subepithelial connective tissue graft for the treatment of isolated mandibular Miller Class I and II gingival recessions: a report of 16 cases. Quintessence Int. 2014 Nov-Dec;45(10):829-35. doi: 10.3290/j.qi.a32636. PubMed PMID: 25191672.
Reason: Not related to the clinical question

Sculean A, Donos N, Blaes A, Lauermann M, Reich E, Brecx M. Comparison of enamel matrix proteins and bioabsorbable membranes in the treatment of intrabony periodontal defects. A split-mouth study. J Periodontol. 1999 Mar;70(3):255-62. PubMed PMID: 10225541.
Reason: Not related to the clinical question

Sculean A, Donos N, Brecx M, Karring T, Reich E. Healing of fenestration-type defects following treatment with guided tissue regeneration or enamel matrix proteins. An experimental study in monkeys. Clin Oral Investig. 2000 Mar;4(1):50-6. PubMed PMID: 11218516.
Reason: Animal study

Sculean A, Donos N, Brecx M, Reich E, Karring T. Treatment of intrabony defects with guided tissue regeneration and enamel-matrix-proteins. An experimental study in monkeys. J Clin Periodontol. 2000 Jul;27(7):466-72. PubMed PMID: 10914886.
Reason: Not related to the clinical question

Sculean A, Donos N, Miliauskaite A, Arweiler N, Brecx M. Treatment of intrabony defects with enamel matrix proteins or bioabsorbable membranes. A 4-year follow-up split-mouth study. J Periodontol. 2001 Dec;72(12):1695-701. PubMed PMID: 11811505.
Reason: Autologous bone wasn't used

Sculean A, Donos N, Reich E, Karring T, Brecx M. Regeneration of oxytalan fibres in different types of periodontal defects: a histological study in monkeys. J Periodontal Res. 1998 Nov;33(8):453-9. PubMed PMID: 9879518.
Reason: Animal study

Sculean A, Donos N, Schwarz F, Becker J, Brecx M, Arweiler NB. Five-year results following treatment of intrabony defects with enamel matrix proteins and guided tissue regeneration. J Clin Periodontol. 2004 Jul;31(7):545-9. PubMed PMID: 15191590.
Reason: Autologous bone wasn't used

Sculean A, Donos N, Windisch P, Brecx M, Gera I, Reich E, Karring T. Healing of human intrabony defects following treatment with enamel matrix proteins or guided tissue regeneration. J Periodontal Res. 1999 Aug;34(6):310-22. PubMed PMID: 10633886.
Reason: Autologous bone wasn't used

Sculean A, Junker R, Donos N, Berakdar M, Brecx M, Dünker N. Immunohistochemical evaluation of matrix molecules associated with wound healing following regenerative periodontal treatment in monkeys. Clin Oral Investig. 2002 Sep;6(3):175-82. Epub 2002 Jul 12. PubMed PMID: 12271352
Tonetti MS, Lang NP, Cortellini P, Suvan JE, Adriaens P, Dubravec D, Fonzar A, Fourmousis I, Mayfield L, Rossi R, Silvestri M, Tiedemann C, Topoll H, Vangsted T, Wallkamm B. Enamel matrix proteins in the regenerative therapy of deep intrabony defects. J Clin Periodontol. 2002 Apr;29(4):317-25. PubMed PMID: 11966929.
Reason: Animal study

Sculean A, Junker R, Donos N, Windisch P, Brecx M, Dünker N. Immunohistochemical evaluation of matrix molecules associated with wound healing following treatment with an enamel matrix protein derivative in humans. Clin Oral Investig. 2003 Sep;7(3):167-74. Epub 2003 Jun 21. PubMed PMID: 12827455.
Reason: Not related to the clinical question

Sculean A, Kiss A, Miliauskaite A, Schwarz F, Arweiler NB, Hannig M. Ten-year results following treatment of intra-bony defects with enamel matrix proteins and guided tissue regeneration. J Clin Periodontol. 2008

Sep;35(9):817-24. doi: 10.1111/j.1600-051X.2008.01295.x. Epub 2008 Jul 21. PubMed PMID: 18647201.
Reason: Autologous bone effect weren't evaluated

Sculean A, Nikolidakis D, Nikou G, Ivanovic A, Chapple IL, Stavropoulos A. Biomaterials for promoting periodontal regeneration in human intrabony defects: a systematic review. Periodontol 2000. 2015 Jun;68(1):182-216. doi: 10.1111/prd.12086. Review. PubMed PMID: 25867987.
Reason: Autologous bone wasn't used

Sculean A, Pietruska M, Arweiler NB, Auschill TM, Nemcovsky C. Four-year results of a prospective-controlled clinical study evaluating healing of intra-bony defects following treatment with an enamel matrix protein derivative alone or combined with a bioactive glass. J Clin Periodontol. 2007 Jun;34(6):507-13. Epub 2007 Apr 23. PubMed PMID: 17451415.
Reason: Biomaterial different from Autologous bone

Sculean A, Rathe F, Junker R, Becker J, Schwarz F, Arweiler N. [The use of Emdogain in periodontal and osseous regeneration]. Schweiz Monatsschr Zahnmed. 2007;117(6):598-606. Review. German. PubMed PMID: 17691421.
Reason: Autologous bone wasn't used

Sculean A, Reich E, Chiantella GC, Brecx M. Treatment of intrabony periodontal defects with an enamel matrix protein derivative (Emdogain): a report of 32 cases. Int J Periodontics Restorative Dent. 1999 Apr;19(2):157-63. PubMed PMID: 10635181.
Reason: Autologous bone wasn't used

Sculean A, Schwarz F, Becker J, Brecx M. The application of an enamel matrix protein derivative (Emdogain) in regenerative periodontal therapy: a review. Med Princ Pract. 2007;16(3):167-80. Review. PubMed PMID: 17409750.
Reason: Autologous bone wasn't used

Sculean A, Schwarz F, Berakdar M, Windisch P, Arweiler NB, Romanos GE. Healing of intrabony defects following surgical treatment with or without an Er:YAG laser. J Clin Periodontol. 2004 Aug;31(8):604-8. PubMed PMID: 15257735.
Reason: Not related to the clinical question

Sculean A, Schwarz F, Chiantella GC, Arweiler NB, Becker J. Nine-year results following treatment of intrabony periodontal defects with an enamel matrix derivative: report of 26 cases. Int J Periodontics Restorative Dent. 2007 Jun;27(3):221-9. PubMed PMID: 17694945.
Reason: Autologous bone wasn't used

Sculean A, Schwarz F, Miliauskaite A, Kiss A, Arweiler N, Becker J, Brecx M. Treatment of intrabony defects with an enamel matrix protein derivative or bioabsorbable membrane: an 8-year follow-up split-mouth study. J Periodontol. 2006 Nov;77(11):1879-86. PubMed PMID: 17076614.
Reason: Not related to the clinical question

Sculean A, Stavropoulos A, Berakdar M, Windisch P, Karring T, Brecx M. Formation of human cementum following different modalities of regenerative therapy. Clin Oral Investig. 2005 Mar;9(1):58-64. Epub 2005 Jan 6. PubMed PMID: 15635476.
Reason: Not related to the clinical question

Sculean A, Windisch P, Chiantella GC. Human histologic evaluation of an intrabony defect treated with enamel matrix derivative, xenograft, and GTR. Int J Periodontics Restorative Dent. 2004 Aug;24(4):326-33. PubMed PMID: 15446402.
Reason: Not related to the clinical question

Sculean A, Windisch P, Chiantella GC, Donos N, Brecx M, Reich E. Treatment of intrabony defects with enamel matrix proteins and guided tissue regeneration. A prospective controlled clinical study. J Clin Periodontol. 2001 May;28(5):397-403. PubMed PMID: 11350501.
Reason: Not related to the clinical question

Sculean A, Windisch P, Döri F, Keglevich T, Molnár B, Gera I. Emdogain in regenerative periodontal therapy. A review of the literature. Fogorv Sz. 2007 Oct;100(5):220-32, 211-9. Review. English, Hungarian. PubMed PMID: 18078142.
Reason: Autologous bone wasn't used

Sculean A, Windisch P, Keglevich T, Chiantella GC, Gera I, Donos N. Clinical

and histologic evaluation of human intrabony defects treated with an enamel matrix protein derivative combined with a bovine-derived xenograft. Int J Periodontics Restorative Dent. 2003 Feb;23(1):47-55. PubMed PMID: 12617368.
Reason: Biomaterial different from autologous bone

Sculean A, Windisch P, Keglevich T, Fabi B, Lundgren E, Lyngstadaas PS. Presence of an enamel matrix protein derivative on human teeth following periodontal surgery. Clin Oral Investig. 2002 Sep;6(3):183-7. Epub 2002 Aug 13. PubMed PMID: 12271353.
Reason: Not related to the clinical question

Sculean A, Windisch P, Keglevich T, Gera I. Clinical and histologic evaluation of an enamel matrix protein derivative combined with a bioactive glass for the treatment of intrabony periodontal defects in humans. Int J Periodontics Restorative Dent. 2005 Apr;25(2):139-47. PubMed PMID: 15839590.
Reason: Biomaterial different from autologous bone

Sculean A, Windisch P, Keglevich T, Gera I. Histologic evaluation of human intrabony defects following non-surgical periodontal therapy with and without application of an enamel matrix protein derivative. J Periodontol. 2003 Feb;74(2):153-60. PubMed PMID: 12666702.
Reason: Not related to the clinical question

Sculean A, Windisch P, Szendröi-Kiss D, Horváth A, Rosta P, Becker J, Gera I, Schwarz F. Clinical and histologic evaluation of an enamel matrix derivative combined with a biphasic calcium phosphate for the treatment of human intrabony periodontal defects. J Periodontol. 2008 Oct;79(10):1991-9. doi: 10.1902/jop.2008.080009 . PubMed PMID: 18834256.
Reason: Biomaterial different from autologous bone

Seshima F, Aoki H, Takeuchi T, Suzuki E, Irokawa D, Makino-Oi A, Sugito H, Tomita S, Saito A. Periodontal regenerative therapy with enamel matrix derivative in the treatment of intrabony defects: a prospective 2-year study. BMC Res Notes. 2017 Jul 6;10(1):256. doi: 10.1186/s13104-017-2572-2. PubMed PMID: 28683765; PubMed Central PMCID: PMC5501118.
Reason: Autologous bone wasn't used

Seshima F, Kigure T, Saito A. Periodontal Regenerative Therapy Using Enamel Matrix Derivative for Treatment of Generalized Severe Chronic Periodontitis: A 2-year Case Report. Bull Tokyo Dent Coll. 2019 Mar 15. doi: 10.2209/tdcpublication.2018-0026. [Epub ahead of print] PubMed PMID: 30880297.
Reason: Autologous bone wasn't used

Seshima F, Nishina M, Namba T, Saito A. Periodontal Regenerative Therapy in Patient with Chronic Periodontitis and Type 2 Diabetes Mellitus: A Case Report. Bull Tokyo Dent Coll. 2016;57(2):97-104. doi: 10.2209/tdcpublication.2015-0041. PubMed PMID: 27320299.
Reason: Not related to the clinical question

Shimizu E, Nakajima Y, Kato N, Nakayama Y, Saito R, Samoto H, Ogata Y. Regulation of rat bone sialoprotein gene transcription by enamel matrix derivative. J Periodontol. 2004 Feb;75(2):260-7. PubMed PMID: 15068114.
Reason: Animal study

Shimizu E, Saito R, Nakayama Y, Nakajima Y, Kato N, Takai H, Kim DS, Arai M, Simmer J, Ogata Y. Amelogenin stimulates bone sialoprotein (BSP) expression through fibroblast growth factor 2 response element and transforming growth factor-beta1 activation element in the promoter of the BSP gene. J Periodontol. 2005 Sep;76(9):1482-9. PubMed PMID: 16171436.
Reason: Not related to the clinical question

Shinohara H, Iwasawa M, Kitazawa T, Kushima H. Functional lip reconstruction with a radial forearm free flap combined with a masseter muscle transfer after wide total excision of the chin. Ann Plast Surg. 2000 Jul;45(1):71-3. PubMed PMID: 10917103.
Reason: Not related to the clinical question

Shirakata Y, Eliezer M, Nemcovsky CE, Weinreb M, Dard M, Sculean A, Bosshardt DD, Moses O. Periodontal healing after application of enamel matrix derivative in surgical supra/infrabony periodontal defects in rats with streptozotocin-induced diabetes. J Periodontal Res. 2014 Feb;49(1):93-101. doi: 10.1111/jre.12084. Epub 2013 Apr 24. PubMed PMID: 23611485.
Reason: Animal study

Shirakata Y, Miron RJ, Nakamura T, Sena K, Shinohara Y, Horai N, Bosshardt
DD, Noguchi K, Sculean A. Effects of EMD liquid (Osteogain) on periodontal
healing in class III furcation defects in monkeys. J Clin Periodontol. 2017
Mar;44(3):298-307. doi: 10.1111/jcpe.12663. Epub 2017 Feb 6. PubMed PMID:
27978604.
Reason: Animal study

Shirakata Y, Miron RJ, Shinohara Y, Nakamura T, Sena K, Horai N, Bosshardt
DD, Noguchi K, Sculean A. Healing of two-wall intra-bony defects treated with a
novel EMD-liquid-A pre-clinical study in monkeys. J Clin Periodontol. 2017
Dec;44(12):1264-1273. doi: 10.1111/jcpe.12825. Epub 2017 Nov 10. PubMed PMID:
28965367.
Reason: Animal study

Shirakata Y, Nakamura T, Shinohara Y, Nakamura-Hasegawa K, Hashiguchi C, Takeuchi N, Imafuji T, Sculean A, Noguchi K. Split-mouth evaluation of connective tissue graft with or without enamel matrix derivative for the treatment of isolated gingival recession defects in dogs. Clin Oral Investig. 2018 Dec 1. doi: 10.1007/s00784-018-2750-1. [Epub ahead of print] PubMed PMID: 30506228.
Reason: Animal study

Shirakata Y, Sculean A, Shinohara Y, Sena K, Takeuchi N, Bosshardt DD,
Noguchi K. Healing of localized gingival recessions treated with a coronally
advanced flap alone or combined with an enamel matrix derivative and a porcine
acellular dermal matrix: a preclinical study. Clin Oral Investig. 2016
Sep;20(7):1791-800. doi: 10.1007/s00784-015-1680-4. Epub 2015 Nov 27. PubMed
PMID: 26612398.
Reason: Not related to the clinical question

Shirakata Y, Takeuchi N, Yoshimoto T, Taniyama K, Noguchi K. Effects of
enamel matrix derivative and basic fibroblast growth factor with μ-tricalcium
phosphate on periodontal regeneration in one-wall intrabony defects: an
experimental study in dogs. Int J Periodontics Restorative Dent. 2013
Sep-Oct;33(5):641-9. doi: 10.11607/prd.0989. PubMed PMID: 23998160.
Shirakata Y, Taniyama K, Yoshimoto T, Miyamoto M, Takeuchi N, Matsuyama T,
Noguchi K. Regenerative effect of basic fibroblast growth factor on periodontal
healing in two-wall intrabony defects in dogs. J Clin Periodontol. 2010
Apr;37(4):374-81. doi: 10.1111/j.1600-051X.2010.01539.x. PubMed PMID: 20447261
Reason: Animal study

Shirakata Y, Yoshimoto T, Goto H, Yonamine Y, Kadomatsu H, Miyamoto M,
Nakamura T, Hayashi C, Izumi Y. Favorable periodontal healing of 1-wall infrabony
defects after application of calcium phosphate cement wall alone or in
combination with enamel matrix derivative: a pilot study with canine mandibles. J
Periodontol. 2007 May;78(5):889-98. PubMed PMID: 17470023.
Reason: Animal study

Shirakata Y, Yoshimoto T, Takeuchi N, Taniyama K, Noguchi K. Effects of EMD
in combination with bone swaging and calcium phosphate bone cement on periodontal
regeneration in one-wall intrabony defects in dogs. J Periodontal Res. 2013
Feb;48(1):37-43. doi: 10.1111/j.1600-0765.2012.01499.x. Epub 2012 Jul 8. PubMed
PMID: 22775137.
Reason: Animal study

Shu R, Liu Z, Ge L. [Influences of porcine enamel matrix proteins on
MC3T3-E1 osteoblast proliferation and differentiation]. Hua Xi Kou Qiang Yi Xue
Za Zhi. 2000 Aug;18(4):226-8. Chinese. PubMed PMID: 12539527.
Reason: Not related to the clinical question

Shujaa Addin A, Akizuki T, Matsuura T, Hoshi S, Ikawa T, Maruyama K, Ono W, Fukuba S, Izumi Y. Histological healing after nonsurgical periodontal treatment with enamel matrix derivatives in canine experimental periodontitis. Odontology. 2018 Jul;106(3):289-296. doi: 10.1007/s10266-018-0347-4. Epub 2018 Feb 10. PubMed PMID: 29429056.
Reason: Animal study

Siciliano VI, Andreuccetti G, Siciliano AI, Blasi A, Sculean A, Salvi GE.
Clinical outcomes after treatment of non-contained intrabony defects with enamel
matrix derivative or guided tissue regeneration: a 12-month randomized controlled
clinical trial. J Periodontol. 2011 Jan;82(1):62-71. doi:
10.1902/jop.2010.100144. Epub 2010 Sep 1. PubMed PMID: 20809859.
Reason: Autologous bone wasn't used

Silvestri M, Rasperini G, Euwe E. Enamel matrix derivative in treatment of
infrabony defects. Pract Periodontics Aesthet Dent. 1999 Jun-Jul;11(5):615-6,
618. PubMed PMID: 10635246.
Reason: Autologous bone wasn't used

Silvestri M, Rasperini G, Milani S. 120 infrabony defects treated with regenerative therapy: long-term results. J Periodontol. 2011 May;82(5):668-75. doi: 10.1902/jop.2010.100297. Epub 2010 Nov 16. PubMed PMID: 21080788.
Reason: Not related to the clinical question

Silvestri M, Ricci G, Rasperini G, Sartori S, Cattaneo V. Comparison of treatments of infrabony defects with enamel matrix derivative, guided tissue regeneration with a nonresorbable membrane and Widman modified flap. A pilot study. J Clin Periodontol. 2000 Aug;27(8):603-10. PubMed PMID: 10959787.
Silvestri M, Sartori S, Rasperini G, Ricci G, Rota C, Cattaneo V. Comparison of infrabony defects treated with enamel matrix derivative versus guided tissue regeneration with a nonresorbable membrane. J Clin Periodontol. 2003 May;30(5):386-93. PubMed PMID: 12716329.
Reason: Autologous bone wasn't used

Siqueira SJ, Ribeiro FV, Villalpando KT, Cirano FR, Pimentel SP. Maintenance periodontal therapy after systemic antibiotic and regenerative therapy of generalized aggressive periodontitis. A case report with 10-year follow-up. Dent Update. 2015 May;42(4):385-6, 389-90, 392-3. PubMed PMID: 26062264.
Reason: Not related to the clinical question

Sipos PM, Loos BG, Abbas F, Timmerman MF, van der Velden U. The combined use of enamel matrix proteins and a tetracycline-coated expanded polytetrafluoroethylene barrier membrane in the treatment of intra-osseous defects. J Clin Periodontol. 2005 Jul;32(7):765-72. PubMed PMID: 15966884.
Reason: Not related to the clinical question

Song AM, Shu R, Xie YF, Song ZC, Li HY, Liu XF, Zhang XL. A study of enamel matrix proteins on differentiation of porcine bone marrow stromal cells into cementoblasts. Cell Prolif. 2007 Jun;40(3):381-96. PubMed PMID: 17531082.
Reason: Not related to the clinical question

Song ZC, Shu R, Zhang XL. Cellular responses and expression profiling of human bone marrow stromal cells stimulated with enamel matrix proteins in vitro. Cell Prolif. 2010 Feb;43(1):84-94. doi: 10.1111/j.1365-2184.2009.00656.x. Epub 2009 Nov 17. PubMed PMID: 19922487.
Reason: Not related to the clinical question

Spahr A, Haegewald S, Tsoulfidou F, Rompola E, Heijl L, Bernimoulin JP, Ring C, Sander S, Haller B. Coverage of Miller class I and II recession defects using enamel matrix proteins versus coronally advanced flap technique: a 2-year report. J Periodontol. 2005 Nov;76(11):1871-80. PubMed PMID: 16274306.
Reason: Not related to the clinical question

St George G, Darbar U, Thomas G. Inflammatory external root resorption following surgical treatment for intra-bony defects: a report of two cases involving Emdogain and a review of the literature. J Clin Periodontol. 2006 Jun;33(6):449-54. Review. PubMed PMID: 16677335.
Reason: Not related to the clinical question

Suárez-López Del Amo F, Monje A, Padial-Molina M, Tang Z, Wang HL. Biologic Agents for Periodontal Regeneration and Implant Site Development. Biomed Res Int. 2015;2015:957518. doi: 10.1155/2015/957518. Epub 2015 Oct 5. Review. PubMed PMID: 26509173; PubMed Central PMCID: PMC4609805.
Reason: Not related to the clinical question

Sugai K, Sato S, Suzuki K, Ito K. Intentional reim plantation of a tooth with severe periodontal involvement using enamel matrix derivative in combination with guided tissue regeneration and bone grafting: a case report. Int J Periodontics Restorative Dent. 2008 Feb;28(1):89-94. PubMed PMID: 18351207.
Reason: Not related to the clinical question

Sugawara A, Sato S. Application of dedifferentiated fat cells for periodontal tissue regeneration. Hum Cell. 2014 Jan;27(1):12-21. doi: 10.1007/s13577-013-0075-6. Epub 2013 Sep 26. PubMed PMID: 24068600.
Reason: Not related to the clinical question

Suphanantachat S, Iwata T, Ishihara J, Yamato M, Okano T, Izumi Y. A role for c-Kit in the maintenance of undifferentiated human mesenchymal stromal cells. Biomaterials. 2014 Apr;35(11):3618-26. doi: 10.1016/j.biomaterials.2014.01.031. Epub 2014 Jan 24. PubMed PMID: 24462355.
Reason: Not related to the clinical question

Svensson Bonde J, Bulow L. One-step purification of recombinant human amelogenin and use of amelogenin as a fusion partner. PLoS One. 2012;7(3):e33269. doi: 10.1371/journal.pone.0033269. Epub 2012 Mar 19. PubMed PMID: 22442680; PubMed Central PMCID: PMC3307724.
Reason: Not related to the clinical question

Swedish Council on Health Technology Assessment. Chronic Periodontitis – Prevention, Diagnosis and Treatment: A Systematic Review [Internet]. Stockholm: Swedish Council on Health Technology Assessment (SBU); 2004 Oct. Available from http://www.ncbi.nlm.nih.gov/books/NBK447960/
PubMed PMID: 28876734.
Reason: Not related to the clinical question

Szatmári P, Gera I. [Treatment of localized intrabony periodontal defects with enamel matrix derivative (Emdogain). Case series]. Fogorv Sz. 2014 Mar;107(1):15-28. Hungarian. PubMed PMID: 24812749.
Reason: Autologous bone wasn't used

Takayanagi K, Osawa G, Nakaya H, Cochran DL, Kamoi K, Oates TW. Effects of enamel matrix derivative on bone-related mRNA expression in human periodontal ligament cells in vitro. J Periodontol. 2006 May;77(5):891-8. PubMed PMID: 16671883.
Reason: Not related to the clinical question

Takeda K, Mizutani K, Matsuura T, Kido D, Mikami R, Noda M, Buranasin P, Sasaki Y, Izumi Y. Periodontal regenerative effect of enamel matrix derivative in diabetes. PLoS One. 2018 Nov 15;13(11):e0207201. doi: 10.1371/journal.pone.0207201. eCollection 2018. PubMed PMID: 30439990; PubMed Central PMCID: PMC6237339.
Reason: Patients with systemic diseases, Autologous bone wasn't used

Taniguchi Y, Aoki A, Sakai K, Mizutani K, Meinzer W, Izumi Y. A Novel Surgical Procedure for Er:YAG Laser-Assisted Periodontal Regenerative Therapy: Case Series. Int J Periodontics Restorative Dent. 2016 Jul-Aug;36(4):507-15. doi: 10.11607/prd.2515. PubMed PMID: 27333008.
Reason: Not related to the clinical question

Tartakovsky Y, Goldstein A, Goldstein M. Radiographic outcomes following treatment of intrabony defects by freeze-dried bone allograft combined with enamel matrix derivative: A retrospective study. Quintessence Int. 2015 Oct;46(9):773-80. doi: 10.3290/j.qi.a34457. PubMed PMID: 26159211.
Reason: Biomaterial different from autologous bone

Taylor AL, Haze-Filderman A, Blumenfeld A, Shay B, Dafni L, Rosenfeld E, Leiser Y, Fermon E, Gruenbaum-Cohen Y, Deutsch D. High yield of biologically active recombinant human amelogenin using the baculovirus expression system. Protein Expr Purif. 2006 Jan;45(1):43-53. Epub 2005 Jun 21. PubMed PMID: 16055347.
Reason: Not related to the clinical question

Terada C, Komasa S, Kusumoto T, Kawazoe T, Okazaki J. Effect of Amelogenin Coating of a Nano-Modified Titanium Surface on Bioactivity. Int J Mol Sci. 2018 Apr 24;19(5). pii: E1274. doi: 10.3390/ijms19051274. PubMed PMID: 29695118; PubMed Central PMCID: PMC5983616.
Reason: Not related to the clinical question

Thalmair T, Fickl S, Bolz W, Wachtel H. The double split flap: a surgical approach for regenerative treatment of interproximal defects. J Clin Periodontol. 2009 Oct;36(10):877-81. doi: 10.1111/j.1600-051X.2009.01461.x. Epub 2009 Aug 3. PubMed PMID: 19663999.
Reason: Not related to the clinical question

Thesleff I, Tummers M. Stem cells and tissue engineering: prospects for regenerating tissues in dental practice. Med Princ Pract. 2003;12 Suppl 1:43-50. Review. PubMed PMID: 12707500.
Reason: Not related to the clinical question

Tobita M, Mizuno H. Adipose-derived stem cells and periodontal tissue engineering. Int J Oral Maxillofac Implants. 2013 Nov-Dec;28(6):e487-93. doi: 10.11607/jomi.te29. Review. PubMed PMID: 24278946.
Reason: Not related to the clinical question

Tokajuk G, Pawińska M, Kedra BA. The clinical and radiological assessment of periodontal bone loss treatment using Emdogain. Adv Med Sci. 2006;51 Suppl 1:227-9. PubMed PMID: 17458097.
Reason: Not related to the clinical question

Tonetti MS, Fourmousis I, Suvan J, Cortellini P, Brägger U, Lang NP; European Research Group on Periodontology (ERGOPERIO). Healing, post-operative morbidity and patient perception of outcomes following regenerative therapy of deep intrabony defects. J Clin Periodontol. 2004 Dec;31(12):1092-8. PubMed PMID: 15560811.
Reason: Not related to the clinical question

Trejo PM, Weltman RL. Favorable periodontal regenerative outcomes from teeth with presurgical mobility: a retrospective study. J Periodontol. 2004 Nov;75(11):1532-8. PubMed PMID: 15633331.
Reason: Not related to the clinical question

Trieger N. An oral surgeon's evaluation of Emdogain. N Y State Dent J. 2003 Nov;69(9):31-3. PubMed PMID: 14702763.
Reason: Autologous bone effects weren't evaluated

Trikka D, Vassilopoulos S. Periodontal Regeneration with Enamel Matrix Derivative in the Management of Generalized Aggressive Periodontitis: A Case Report with 11-Year Follow-up and Literature Review. J Int Soc Prev Community Dent. 2019 Jan-Feb;9(1):13-20. doi: 10.4103/jispcd.JISPCD_119_18. Epub 2019 Feb 14. Review. PubMed PMID: 30923688; PubMed Central PMCID: PMC6402248.
Reason: Autologous bone wasn't used

Troiano G, Laino L, Zhurakivska K, Cicciù M, Lo Muzio L, Lo Russo L. Addition of enamel matrix derivatives to bone substitutes for the treatment of intrabony defects: A systematic review, meta-analysis and trial sequential analysis. J Clin Periodontol. 2017 Jul;44(7):729-738. doi: 10.1111/jcpe.12742. Epub 2017 Jun 23. Review. PubMed PMID: 28477366.
Reason: Autologous bone wasn't used

Trombelli L, Annunziata M, Belardo S, Farina R, Scabbia A, Guida L. Autogenous bone graft in conjunction with enamel matrix derivative in the treatment of deep periodontal intra-osseous defects: a report of 13 consecutively treated patients. J Clin Periodontol. 2006 Jan;33(1):69-75. PubMed PMID: 16367859.
Reason: Case series

Trombelli L. Which reconstructive procedures are effective for treating the periodontal intraosseous defect? Periodontol 2000. 2005;37:88-105. PubMed PMID: 15655027.
Reason: Not related to the clinical question

Trombelli L, Bottega S, Zucchelli G. Supracrestal soft tissue preservation with enamel matrix proteins in treatment of deep intrabony defects. J Clin Periodontol. 2002 May;29(5):433-9. PubMed PMID: 12060426.
Reason: Autologous bone wasn't used

Trombelli L, Farina R. Clinical outcomes with bioactive agents alone or in combination with grafting or guided tissue regeneration. J Clin Periodontol. 2008 Sep;35(8 Suppl):117-35. doi: 10.1111/j.1600-051X.2008.01265.x. Review. PubMed PMID: 18724846.
Reason: Autologous bone wasn't used

Trombelli L, Farina R, Minenna L, Toselli L, Simonelli A. Regenerative Periodontal Treatment with the Single Flap Approach in Smokers and Nonsmokers. Int J Periodontics Restorative Dent. 2018 Jul/Aug;38(4):e59-e67. doi: 10.11607/prd.3615. PubMed PMID: 29889915.
Reason: Not related to the clinical question

Trombelli L, Scabbia A, Wikesjö UM, Calura G. Fibrin glue application in conjunction with tetracycline root conditioning and coronally positioned flap procedure in the treatment of human gingival recession defects. J Clin Periodontol. 1996 Sep;23(9):861-7. PubMed PMID: 8891938.
Reason: Not related to the clinical question

Trombelli L, Simonelli A, Minenna L, Rasperini G, Farina R. Effect of a Connective Tissue Graft in Combination With a Single Flap Approach in the Regenerative Treatment of Intraosseous Defects ([Formula: see text]). J Periodontol. 2017 Apr;88(4):348-356. doi: 10.1902/jop.2016.160471. Epub 2016 Dec 13. PubMed PMID: 27958767.
Reason: Not related to the clinical question

Trombelli L, Simonelli A, Minenna L, Rasperini G, Farina R. Effect of a Connective Tissue Graft in Combination With a Single Flap Approach in the Regenerative Treatment of Intraosseous Defects. J Periodontol. 2017 Apr;88(4):348-356. doi: 10.1902/jop.2016.160471. PubMed PMID: 29537650.
Reason: Not related to the clinical question

Tsitoura E, Tucker R, Suvan J, Laurell L, Cortellini P, Tonetti M. Baseline radiographic defect angle of the intrabony defect as a prognostic indicator in regenerative periodontal surgery with enamel matrix derivative. J Clin Periodontol. 2004 Aug;31(8):643-7. PubMed PMID: 15257742
Reason: Autologous bone wasn't used

Tu YK, Needleman I, Chambrone L, Lu HK, Faggion CM Jr. A Bayesian network meta-analysis on comparisons of enamel matrix derivatives, guided tissue regeneration and their combination therapies. J Clin Periodontol. 2012 Mar;39(3):303-14. PubMed PMID: 22393565.
Reason: Autologous bone wasn't used

Tu YK, Tugnait A, Clerehugh V. Is there a temporal trend in the reported treatment efficacy of periodontal regeneration? A meta-analysis of randomized-controlled trials. J Clin Periodontol. 2008 Feb;35(2):139-46. Epub 2007 Dec 13. PubMed PMID: 18081861.
Reason: Not related to the clinical question

Tu YK, Woolston A, Faggion CM Jr. Do bone grafts or barrier membranes provide additional treatment effects for infrabony lesions treated with enamel matrix derivatives? A network meta-analysis of randomized-controlled trials. J Clin Periodontol. 2010 Jan;37(1):59-79. doi: 10.1111/j.1600-051X.2009.01499.x. Epub 2009 Dec 1. PubMed PMID: 19958442.
Reason: Autologous bone wasn't used

Vandana KL, Shah K, Prakash S. Clinical and radiographic evaluation of Emdogain as a regenerative material in the treatment of interproximal vertical defects in chronic and aggressive periodontitis patients. Int J Periodontics Restorative Dent. 2004 Apr;24(2):185-91. PubMed PMID: 15119889.
Reason: Autologous bone wasn't used

Van Swol RL, Ellinger R, Pfeifer J, Barton NE, Blumenthal N. Collagen membrane barrier therapy to guide regeneration in Class II furcations in humans. J Periodontol. 1993 Jul;64(7):622-9. PubMed PMID: 8366412.
Reason: Not related to the clinical question

Velasquez-Plata D, Scheyer ET, Mellonig JT. Clinical comparison of an enamel matrix derivative used alone or in combination with a bovine-derived xenograft for the treatment of periodontal osseous defects in humans. J Periodontol. 2002 Apr;73(4):433-40. Erratum in: J Periodontol 2002 Jun;73(6):684. PubMed PMID: 11990445.
Reason: Biomaterial different from autologous bone

Venezia E, Goldstein M, Boyan BD, Schwartz Z. The use of enamel matrix derivative in the treatment of periodontal defects: a literature review and meta-analysis. Crit Rev Oral Biol Med. 2004 Nov 1;15(6):382-402. Review. PubMed PMID: 15574680.
Reason: Autologous bone wasn't used

Venezia E, Goldstein M, Schwartz Z. [The use of enamel matrix derivative in periodontal therapy]. Refuat Hapeh Vehashinayim (1993). 2002 Jul;19(3):19-34, 88. Review. Hebrew. PubMed PMID: 12242763.
Reason: Autologous bone wasn't used

Verardi S. The use of a membrane and/or a bone graft may not improve the effects of enamel matrix derivatives in infrabony defects. J Evid Based Dent Pract. 2012 Sep;12(3 Suppl):127-8. doi: 10.1016/S1532-3382(12)70024-6. PubMed PMID: 23253838.
Reason: Autologous bone effects weren't evaluated

Wachtel H, Schenk G, Böhm S, Weng D, Zuhr O, Hürzeler MB. Microsurgical access flap and enamel matrix derivative for the treatment of periodontal intrabony defects: a controlled clinical study. J Clin Periodontol. 2003 Jun;30(6):496-504. PubMed PMID: 12795787.
Reason: Autologous bone wasn't used

Walker CG, Ito Y, Dangaria S, Luan X, Diekwisch TG. RANKL, osteopontin, and osteoclast homeostasis in a hyperocclusion mouse model. Eur J Oral Sci. 2008 Aug;116(4):312-8. doi: 10.1111/j.1600-0722.2008.00545.x. PubMed PMID: 18705798; PubMed Central PMCID: PMC2597431.
Reason: Not related to the clinical question

Wang HL, Greenwell H, Fiorellini J, Giannobile W, Offenbacher S, Salkin L,

Townsend C, Sheridan P, Genco RJ; Research, Science and Therapy Committee. Periodontal regeneration. J Periodontol. 2005 Sep;76(9):1601-22. Review. PubMed PMID: 16171453.
Reason: Not related to the clinical question

Wang XJ, Huang H, Yang F, Xia LG, Zhang WJ, Jiang XQ, Zhang FQ. Ectopic study of tissue-engineered bone complex with enamel matrix proteins, bone marrow stromal cells in porous calcium phosphate cement scaffolds, in nude mice. Cell Prolif. 2011 Jun;44(3):274-82. doi: 10.1111/j.1365-2184.2011.00750.x. PubMed PMID: 21535268.
Reason: Biomaterial different from autologous bone

Wang Y, Zhao Y, Ge L. Effects of the enamel matrix derivative on the proliferation and odontogenic differentiation of human dental pulp cells. J Dent. 2014 Jan;42(1):53-9. doi: 10.1016/j.jdent.2013.10.020. Epub 2013 Nov 15. PubMed PMID: 24246686.
Reason: Not related to the clinical question

Watanabe K, Kikuchi M, Okumura M, Kadosawa T, Fujinaga T. Efficacy of enamel matrix protein applied to spontaneous periodontal disease in two dogs. J Vet Med Sci. 2003 Sep;65(9):1007-10. PubMed PMID: 14532695.
Reason: Animal study

Watanabe K, Kikuchi M, Okumura M, Kadosawa T, Fujinaga T. Efficacy of enamel matrix proteins on apical periodontal regeneration after experimental apicoectomy in dogs. J Vet Med Sci. 2001 Aug;63(8):889-94. PubMed PMID: 11558545.
Reason: Not related to the clinical question

Weishaupt P, Bernimoulin JP, Trackman P, Hägewald S. Stimulation of osteoblasts with Emdogain increases the expression of specific mineralization markers. Oral Surg Oral Med Oral Pathol Oral Radiol Endod. 2008 Aug;106(2):304-8. doi: 10.1016/j.tripleo.2008.02.033. Epub 2008 Jun 11. PubMed PMID: 18547835.
Reason: Autologous bone wasn't used

Wikesjö UM, Bogle GC, Nilvéus RE. Periodontal repair in dogs: effect of a composite graft protocol on healing in supraalveolar periodontal defects. J Periodontol. 1992 Feb;63(2):107-13. PubMed PMID: 1313105.
Reason: Animal study

Windisch P, Sculean A, Klein F, Tóth V, Eickholz P, István G. [Comparative analysis of the sensitivity and accuracy of clinical, radiographic and histometric measurements in assessing periodontal attachment levels]. Fogorv Sz. 2002 Jun;95(3):93-8. Hungarian. PubMed PMID: 12141193.
Reason: Not related to the clinical question

Windisch P, Sculean A, Klein F, Tóth V, Gera I, Reich E, Eickholz P. Comparison of clinical, radiographic, and histometric measurements following treatment with guided tissue regeneration or enamel matrix proteins in human periodontal defects. J Periodontol. 2002 Apr;73(4):409-17. PubMed PMID: 11990442.
Reason: Not related to the clinical question

Wolnerman JS, Bergman Z, Zini A, Mizrahi B, Vered Y, Domb AJ, Mann J. [A new gel for topical use in treating severe periodontal disease--clinical observations]. Refuat Hapeh Vehashinayim (1993). 2004 Apr;21(2):72-7, 99. Hebrew. PubMed PMID: 15503549.
Reason: Not related to the clinical question

Wu SM, Chiu HC, Chin YT, Lin HY, Chiang CY, Tu HP, Fu MM, Fu E. Effects of enamel matrix derivative on the proliferation and osteogenic differentiation of human gingival mesenchymal stem cells. Stem Cell Res Ther. 2014 Apr 16;5(2):52. doi: 10.1186/scrt441. PubMed PMID: 24739572; PubMed Central PMCID: PMC4076631.
Reason: Not related to the clinical question

Xu L, Yang Z, Jin F, Duan Y, Jin Y. Characterization of rat apical tissues in different root development stage. Connect Tissue Res. 2011 Oct;52(5):393-400. doi: 10.3109/03008207.2010.544429. Epub 2011 Mar 15. PubMed PMID: 21405979.
Reason: Animal study

Xu ZF, Bai S, Zhang ZQ, Duan WY, Wang ZQ, Sun CF. A critical assessment of the fibula flap donor site. Head Neck. 2017 Feb;39(2):279-287. doi: 10.1002/hed.24581. Epub 2016 Sep 12. PubMed PMID: 27617706.
Reason: Not related to the clinical question

Yamamoto S, Masuda H, Shibukawa Y, Yamada S. Combination of bovine-derived xenografts and enamel matrix derivative in the treatment of intrabony periodontal defects in dogs. Int J Periodontics Restorative Dent. 2007 Oct;27(5):471-9. PubMed PMID: 17990444.
Reason: Biomaterial different from autologous bone

Yamamoto Y, Sugihara T, Furuta Y, Fukuda S. Functional reconstruction of the tongue and deglutition muscles following extensive resection of tongue cancer. Plast Reconstr Surg. 1998 Sep;102(4):993-8; discussion 999-1000. PubMed PMID: 9734414.
Reason: Not related to the clinical question

Yan XZ, Rathe F, Gilissen C, van der Zande M, Veltman J, Junker R, Yang F, Jansen JA, Walboomers XF. The effect of enamel matrix derivative (Emdogain®) on gene expression profiles of human primary alveolar bone cells. J Tissue Eng Regen Med. 2014 Jun;8(6):463-72. doi: 10.1002/term.1545. Epub 2012 Jun 11. PubMed PMID: 22689476.
Reason: Not related to the clinical question

Yang S, Lan L, Miron RJ, Wei L, Zhang M, Zhang Y. Variability in Particle Degradation of Four Commonly Employed Dental Bone Grafts. Clin Implant Dent Relat Res. 2015 Oct;17(5):996-1003. doi: 10.1111/cid.12196. Epub 2014 Jan 3. PubMed PMID: 24393521.
Reason: Not related to the clinical question

Yilmaz S, Kuru B, Altuna-Kiraç E. Enamel matrix proteins in the treatment of periodontal sites with horizontal type of bone loss. J Clin Periodontol. 2003 Mar;30(3):197-206. PubMed PMID: 12631177.
Reason: Autologous bone wasn't used

Yin X, Li Y, Li J, Li P, Liu Y, Wen J, Luan Q. Generation and periodontal differentiation of human gingival fibroblasts-derived integration-free induced pluripotent stem cells. Biochem Biophys Res Commun. 2016 May 6;473(3):726-32. doi: 10.1016/j.bbrc.2015.10.012. Epub 2015 Oct 9. PubMed PMID: 26456649.
Reason: Not related to the clinical question

Yoleri L, Mavioğlu H. Total tongue reconstruction with free functional gracilis muscle transplantation: a technical note and review of the literature. Ann Plast Surg. 2000 Aug;45(2):181-6. Review. PubMed PMID: 10949348.

Yoneda S. [The effects of enamel matrix derivative (EMD) on osteoblastic cells]. Kokubyo Gakkai Zasshi. 2002 Sep;69(3):207-14. Japanese. PubMed PMID: 12400176.
Reason: Not related to the clinical question

Yoneda S, Itoh D, Kuroda S, Kondo H, Umezawa A, Ohya K, Ohyama T, Kasugai S. The effects of enamel matrix derivative (EMD) on osteoblastic cells in culture and bone regeneration in a rat skull defect. J Periodontal Res. 2003 Jun;38(3):333-42. PubMed PMID: 12753373.
Reason: Animal study

Yukna RA, Mellonig JT. Histologic evaluation of periodontal healing in humans following regenerative therapy with enamel matrix derivative. A 10-case series. J Periodontol. 2000 May;71(5):752-9. PubMed PMID: 10872956.
Reason: Not related to the clinical question

Zanatta FB, de Souza FG, Pinto TM, Antoniazzi RP, Rösing CK. Do the clinical effects of enamel matrix derivatives in infrabony defects decrease overtime? A systematic review and meta-analysis. Braz Dent J. 2013 Sep-Oct;24(5):446-55. doi: 10.1590/0103-6440201302192. Review. PubMed PMID: 24474283.
Reason: Not related to the clinical question

Zeichner-David M. Regeneration of periodontal tissues: cementogenesis revisited. Periodontol 2000. 2006;41:196-217. Review. PubMed PMID: 16686935.
Reason: Not related to the clinical question

Zeichner-David M, Chen LS, Hsu Z, Reyna J, Caton J, Bringas P. Amelogenin and ameloblastin show growth-factor like activity in periodontal ligament cells. Eur J Oral Sci. 2006 May;114 Suppl 1:244-53; discussion 254-6, 381-2. PubMed PMID: 16674693.
Reason: Not related to the clinical question

Zeldich E, Koren R, Nemcovsky C, Weinreb M. Enamel matrix derivative

stimulates human gingival fibroblast proliferation via ERK. J Dent Res. 2007 Jan;86(1):41-6. PubMed PMID: 17189461.
Reason: Not related to the clinical question

Zetterström O, Andersson C, Eriksson L, Fredriksson A, Friskopp J, Heden G, Jansson B, Lundgren T, Nilveus R, Olsson A, Renvert S, Salonen L, Sjöström L, Winell A, Ostgren A, Gestrelius S. Clinical safety of enamel matrix derivative (EMDOGAIN) in the treatment of periodontal defects. J Clin Periodontol. 1997 Sep;24(9 Pt 2):697-704. PubMed PMID: 9310875.
Reason: Not related to the clinical question

Zhang YF. [Bioinductive biomaterials for periodontal regeneration]. Zhonghua Kou Qiang Yi Xue Za Zhi. 2017 Oct 9;52(10):615-619. doi: 10.3760/cma.j.issn.1002-0098.2017.10.007. Review. Chinese. PubMed PMID: 29972935.
Reason: Autologous bone effect weren't evaluated

Zhang Y, Jing D, Buser D, Sculean A, Chandad F, Miron RJ. Bone grafting material in combination with Osteogain for bone repair: a rat histomorphometric study. Clin Oral Investig. 2016 Apr;20(3):589-95. doi: 10.1007/s00784-015-1532-2. Epub 2015 Jul 15. PubMed PMID: 26174082.
Zhang FQ, Meng HX, Han J, Liu KN. [Effects of emdogain on human periodontal ligament cells in vitro]. Beijing Da Xue Xue Bao Yi Xue Ban. 2012 Feb 18;44(1):6-10. Chinese. PubMed PMID: 22353891.
Reason: Animal study

Zhou S, Sun C, Huang S, Wu X, Zhao Y, Pan C, Wang H, Liu J, Li Q, Kou Y. Efficacy of Adjunctive Bioactive Materials in the Treatment of Periodontal Intrabony Defects: A Systematic Review and Meta-Analysis. Biomed Res Int. 2018 May 27;2018:8670832. doi: 10.1155/2018/8670832. eCollection 2018. PubMed PMID: 29977919; PubMed Central PMCID: PMC5994283.
Reason: Autologous bone effect weren't evaluated

Zilm PS, Bartold PM. Proteomic identification of proteinase inhibitors in the porcine enamel matrix derivative, EMD(®). J Periodontal Res. 2011 Feb;46(1):111-7. doi: 10.1111/j.1600-0765.2010.01320.x. Epub 2010 Nov 26. PubMed PMID: 21108643.
Reason: Not related to the clinical question

Zucchelli G, Amore C, Montebugnoli L, De Sanctis M. Enamel matrix proteins and bovine porous bone mineral in the treatment of intrabony defects: a comparative controlled clinical trial. J Periodontol. 2003 Dec;74(12):1725-35. PubMed PMID: 14974812.
Reason: Biomaterial different from autologous bone

Zucchelli G, Bernardi F, Montebugnoli L, De SM. Enamel matrix proteins and guided tissue regeneration with titanium-reinforced expanded polytetrafluoroethylene membranes in the treatment of infrabony defects: a comparative controlled clinical trial. J Periodontol. 2002 Jan;73(1):3-12. PubMed PMID: 11846197.
Reason: Not related to the clinical question

Zucchelli G. Long-term maintenance of an apparently hopeless tooth: a case report. Eur J Esthet Dent. 2007 Winter;2(4):390-404. PubMed PMID: 19655507.
Reason: Not related to the clinical question

Zucchelli G, De Sanctis M. A novel approach to minimizing gingival recession in the treatment of vertical bony defects. J Periodontol. 2008 Mar;79(3):567-74. doi: 10.1902/jop.2008.070315 . PubMed PMID: 18315442.
Reason: Not related to the clinical question

Zucchelli G, Mazzotti C, Tirone F, Mele M, Bellone P, Mounssif I. The connective tissue graft wall technique and enamel matrix derivative to improve root coverage and clinical attachment levels in Miller Class IV gingival recession. Int J Periodontics Restorative Dent. 2014 Sep-Oct;34(5):601-9. PubMed PMID: 25171030.
Reason: Autologous bone wasn't used

Zucchelli G, Mele M, Checchi L. The papilla amplification flap for the treatment of a localized periodontal defect associated with a palatal groove. J Periodontol. 2006 Oct;77(10):1788-96. PubMed PMID: 17032124.
Reason: Not related to the clinical question

Zucchelli G, Mounssif I, Marzadori M, Mazzotti C, Felice P, Stefanini M. Connective Tissue Graft Wall Technique and Enamel Matrix Derivative for the Treatment of Infrabony Defects: Case Reports. Int J Periodontics Restorative Dent. 2017 Sep/Oct;37(5):673-681. doi: 10.11607/prd.3083. PubMed PMID: 28817131.

Reason: Not related to the clinical question

.
